# A novel approach to risk exposure and epigenetics—the use of multidimensional context to gain insights into the early origins of cardiometabolic and neurocognitive health

**DOI:** 10.1186/s12916-023-03168-z

**Published:** 2023-11-27

**Authors:** Jane W. Y. Ng, Janine F. Felix, David M. Olson

**Affiliations:** 1 Department of Pediatrics, Cummings School of Medicine, University of Calgary, 28 Oki Drive NW, Calgary, AB T3B 6A8 Canada; 2 The Generation F Study Group, Erasmus MC University Medical Center Rotterdam, Postbus, 2040, 3000 CA Rotterdam, The Netherlands; 3 Department of Pediatrics, Erasmus MC University Medical Center Rotterdam, Rotterdam, The Netherlands; 4 Departments of Obstetrics and Gynecology, Physiology, and Pediatrics, Faculty of Medicine and Dentistry, University of Alberta, 220 HMRC, Edmonton, AB T6G2S2 Canada

**Keywords:** Epigenetics, Maternal health, Child health, ALSPAC, Generation R Study, Noncommunicable diseases, Developmental origins of health and disease, Health determinants, Smoking, Multivariate analysis

## Abstract

**Background:**

Each mother–child dyad represents a unique combination of genetic and environmental factors. This constellation of variables impacts the expression of countless genes. Numerous studies have uncovered changes in DNA methylation (DNAm), a form of epigenetic regulation, in offspring related to maternal risk factors. How these changes work together to link maternal-child risks to childhood cardiometabolic and neurocognitive traits remains unknown. This question is a key research priority as such traits predispose to future non-communicable diseases (NCDs). We propose viewing risk and the genome through a multidimensional lens to identify *common* DNAm patterns shared among *diverse* risk profiles.

**Methods:**

We identified multifactorial Maternal Risk Profiles (MRPs) generated from population-based data (*n* = 15,454, Avon Longitudinal Study of Parents and Children (ALSPAC)). Using cord blood HumanMethylation450 BeadChip data, we identified genome-wide patterns of DNAm that co-vary with these MRPs. We tested the prospective relation of these DNAm patterns (*n* = 914) to future outcomes using decision tree analysis. We then tested the reproducibility of these patterns in (1) DNAm data at age 7 and 17 years within the same cohort (*n* = 973 and 974, respectively) and (2) cord DNAm in an independent cohort, the Generation R Study (*n* = 686).

**Results:**

We identified twenty MRP-related DNAm patterns at birth in ALSPAC. Four were prospectively related to cardiometabolic and/or neurocognitive childhood outcomes. These patterns were replicated in DNAm data from blood collected at later ages. Three of these patterns were externally validated in cord DNAm data in Generation R. Compared to previous literature, DNAm patterns exhibited novel spatial distribution across the genome that intersects with chromatin functional and tissue-specific signatures.

**Conclusions:**

To our knowledge, we are the first to leverage multifactorial population-wide data to detect patterns of variability in DNAm. This context-based approach decreases biases stemming from overreliance on specific samples or variables. We discovered molecular patterns demonstrating prospective and replicable relations to complex traits. Moreover, results suggest that patterns harbour a genome-wide organisation specific to chromatin regulation and target tissues. These preliminary findings warrant further investigation to better reflect the reality of human context in molecular studies of NCDs.

**Graphical Abstract:**

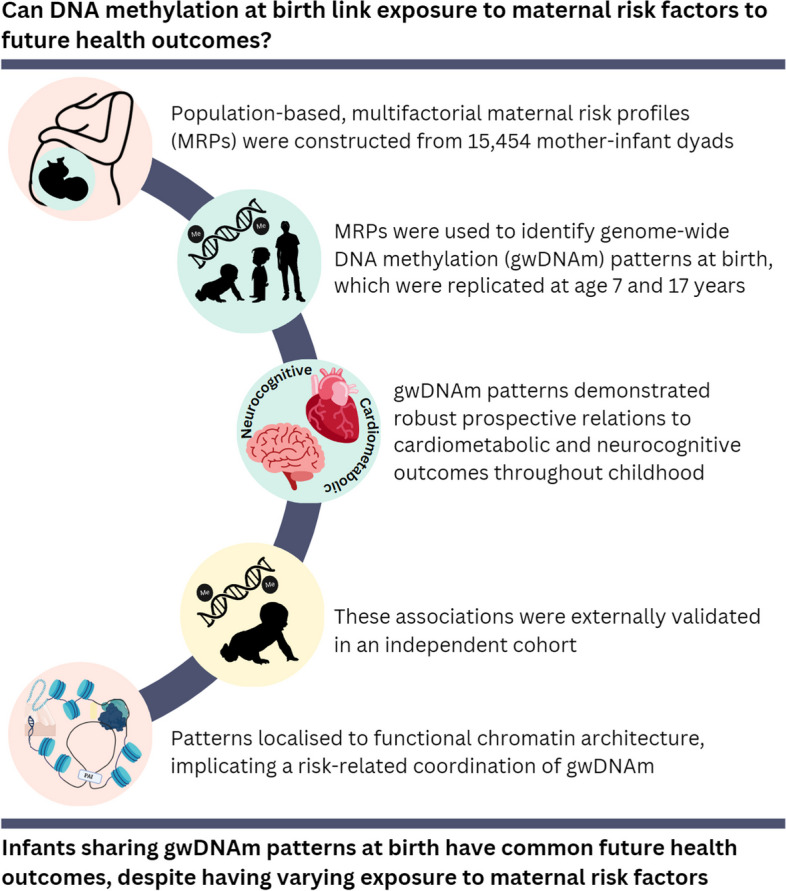

**Supplementary Information:**

The online version contains supplementary material available at 10.1186/s12916-023-03168-z.

## Background

Identifying the impact of maternal risk factors on foetal health and child outcomes is challenging because each mother–child dyad represents a unique combination of genetic, environmental, and random chance effects. This complexity accounts for why identical twins demonstrate differences in health outcomes or why foetal alcohol exposure can have a broad spectrum of features rather than a single phenotype. Moreover, the range and complexity of maternal risk factors that impact the foetus go beyond direct in utero effects and can include intergenerational effects [1].

Regardless of the sources or types of risk, research suggests that such multifactorial risk exposures set off a ripple effect across the human genome, impacting the regulation of a multitude of biological pathways [2]. It is believed that risk exposure leads to disease in offspring due to “foetal programming”, a concept arising from the developmental origins of disease hypothesis (DOHaD) [3]. Epigenetic modifications of the genome are mechanisms through which risk exposure may dysregulate foetal programming [4, 5]. These non-genetic molecular changes to the genome modify the DNA’s physical 3D organisation and its interaction with nuclear machinery [6, 7]. The readiness of genes to be expressed is thus altered. Epigenetic modifications can work together to change the gene expression patterns of the entire cell. The best-studied epigenetic mechanism in humans is DNA methylation (DNAm). DNAm describes the addition of a methyl group to a cytosine base located upstream of a guanine base in a DNA sequence, which together is known as a CpG site.

Most research attempts to pinpoint single or neighbouring clusters of CpG sites associated with a given risk or outcome, such as smoking or birth weight. Researchers now venture to connect these points across multiple sites on different chromosomes. For instance, Elliott and colleagues (2014) generated a smoking methylation score in adults that combined 183 CpG sites to distinguish never and former smokers from current smokers. This methylation score separated these two categories with 100% sensitivity and 97% specificity in a male population of European descent. When multiple sites are linked*,* theoretical and empirical evidence suggests that DNAm can provide insight into an individual’s past experiences.

Epigenetic analyses that focus on discrete risk exposures and molecular sites create models that are easier to understand and analyse, but these models do not reflect the clinical reality of non-communicable diseases (NCDs). Each person experiences a unique array of risks whose genomic targets are interactive and multidimensional in nature. Hence, we propose to view risk exposure and the genome as interconnected parts of an engine that drives the trajectory of health. This proof-of-concept study tests this idea of integrating context into statistical models using basic multidimensional methods.

To begin, we asked how to address the challenge of examining the phenomenon of unique risk exposures in each individual across a population. We decided to develop a maternal risk profile (MRP) centred around the maternal behaviour of smoking while pregnant. This health behaviour is a telling marker of stress among physical, mental, and social determinants of disease [8]. Smoking during pregnancy is often linked to other lifestyle risk factors (e.g. drug and/or alcohol abuse) and stressors (e.g. lower socioeconomic status [9]). It also has known direct effects on the foetal environment and physiology [10]. Lastly, it is a common foetal exposure linked to a broad range of NCDs in later life [11–16]. By anchoring foetal risk to the multifactorial phenomenon of maternal smoking, we hope to capture a risk profile that intersects health domains as would occur in clinical reality.

From a physiological perspective, exposure to MRP may behave like other stresses and have a normative range within the general population. Therefore, we used multiple variables related to maternal smoking in previous literature to generate a population-based MRP. This reduces the chance of drawing conclusions that only pertain to a subset of the population.

Next, we considered how to view the molecular components. A multivariate model of associated DNAm changes may be particularly applicable in the study of NCDs. These diseases are etiologically distinct from processes like cancers that typically can be traced to single molecular events. NCDs tend to arise from many small and dynamic cellular changes that lead to an overall shift in the cellular and physiological state of the whole person. These multiple small changes reflect the interplay of genes, environment, and random chance.

**Fig. 1:**
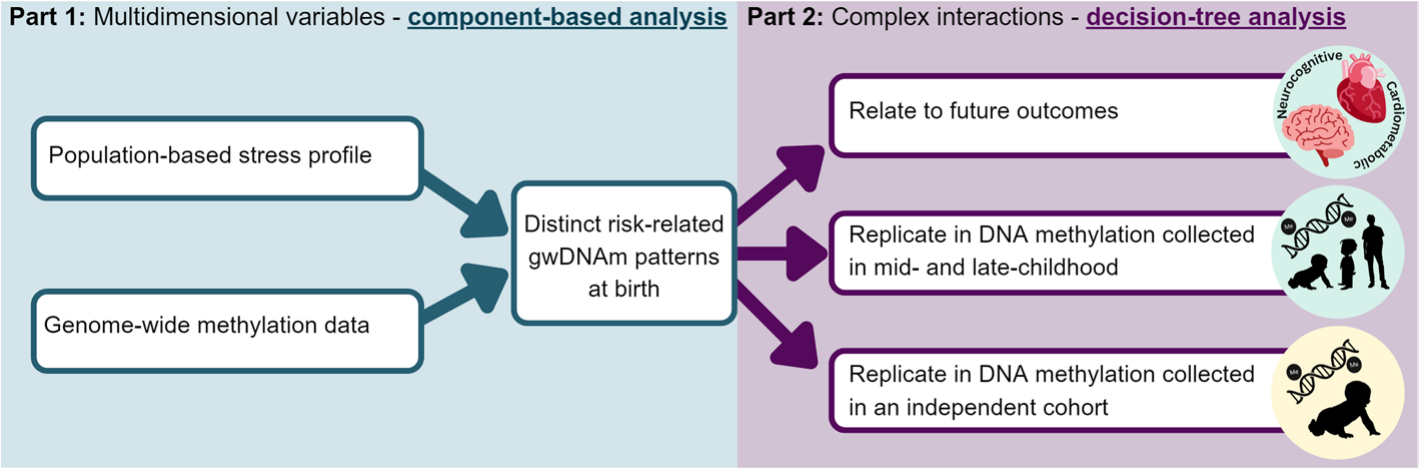
Analysis workflow schematic. Part 1 (aqua) involves component-based analysis to integrate two multidimensional data sets, risk and DNAm. We obtain a new set of variables representing variability in methylation at birth that reflect risk exposure, which we call genome-wide DNAm patterns (gwDNAm patterns). Part 2 (purple) involves observing the interaction of variables and covariates that could lead to a given outcome using decision tree analysis. This is directly compared to relations to outcomes that could arise through random chance alone. In this way, this analysis can inform us of robust relations between heterogeneous risks and complex traits

We theorise that coordinated shifts in genome-wide DNAm (gwDNAm) could be the common mechanistic link between individuals who encounter different maternal influences yet share similar phenotypic traits. We tested this by seeking recurring patterns of gwDNAm in cord blood that relate to population-based MRPs. In this way, gwDNAm patterns represent population-normalised molecular signatures of risk exposure. We investigated how these gwDNAm patterns may relate to the development of NCDs by observing their relation to anthropometric and neurodevelopmental child outcomes. We repeated this analysis over time as children aged and in an independent cohort. We outline our analysis workflow in Fig. 1.

## Methods

### Discovery cohort—ALSPAC

#### Participants

Model discovery was conducted in the population-based ALSPAC longitudinal cohort. This initial cohort collected data on 14,541 pregnancies and 14,676 foetuses in the former county of Avon, United Kingdom (UK) with expected delivery dates from 1 April 1991 to 31 December 1992. This cohort has been extensively described [17, 18] with specific details regarding variables pertinent to our analysis available in Additional file 1.

#### Exposure variables: maternal risk profiles

To develop a multidimensional risk profile from the viewpoint of foetal programming, we apply a context-based approach that uses population-based data to integrate multiple MRP-related variables. The normative values for blood pressure in children exist as continuous ranges that are based on sex, age, and height percentiles. Similarly, we regard to foetal risk exposure as a continuous and interdependent variable. The risk factors traverse gestational (e.g. maternal smoking in pregnancy, pregnancy factors, foetal health and growth, etc.), family (e.g. smoking in the father, grandparents, other household members, etc.), and social effects (e.g. education, vocation, etc.). These data were gathered from maternal self-reports and linked clinical records.

#### Outcomes: child physical and mental development

We selected outcomes based on their relevance to maternal smoking exposure (Table 1) as identified in previous literature [14, 19–21]. All cardiometabolic outcome values were converted to internal* z*-scores (sex-specific) using data from the entire ALSPAC cohort unless otherwise specified. To control for the impact of early weight and length accrual on later life physical outcomes, individual infant growth trajectories calculated for this cohort using multilevel modelling were included in relevant models [22]. At age 18, study children were sent “fair processing” materials describing ALSPAC’s intended use of their health and administrative records, and they were given clear means to consent or object via a written form. Data were not extracted for participants who objected or who were not sent fair processing materials.

**Table 1:** Methods for measuring child outcomes

		Methods
Neurocognitive	Development	• Child development was measured using parental report at 6, 18, and 30 months using a questionnaire adapted from the Denver Developmental Screening Test—II [23]
	Academic Performance	• UK Department of Education scores from standard assessment tests linked to ALSPAC subjects for ages 5–7, 8–11, and 12–14 years
	Cognitive Development	• Researcher-administered Wechsler Intelligence Scale for Children-III UK at age 4 and 8 years
Cardiometabolic	Body Composition	• Dual-energy x-ray absorptiometry measured fat, lean, and bone mass at 9, 11, and 13 years
	Anthropometric/Blood Pressure	• Weight, waist circumference, and blood pressure were measured throughout infancy and in 1–2-year intervals from ages 7 to 13 years

#### DNA methylation data

ARIES is a sub-study of ALSPAC child-mother pairs with available DNAm data. Blood samples were collected at three time points: birth (cord blood) and at ages 7 and 17 years [24]. At later ages, DNA was extracted from either the buffy coat or whole blood samples. DNAm data measurements were performed using the Illumina Infinium HumanMethylation450 BeadChip (450K BeadChip). The technical details of data normalisation, filtering unreliable or low variance probes, and managing batch effect, blood cell type composition, and confounders are available in Additional file 1. This left 185,466 CpG sites remaining. DNAm beta values were used in all analyses.

### Validation cohort—Generation R Study

The Generation R Study (GenR) is a population-based prospective pregnancy cohort study. It included 9778 women and their children born between April 2002 and January 2006. We used cord blood 450K BeadChip DNAm data from a subgroup consisting of 969 children of European descent described in detail [25]. In total, 686 children had complete data for these analyses. Besides detailed pregnancy data, this cohort collected substantial offspring data, including anthropometric data at birth, in infancy, and at ages 6, 10, and 13 years.

### Statistical analysis

In part one of the study (see Fig. 1, aqua), we needed to link the multifactorial effect of risk to thousands of DNAm sites across the genome. We employed component-based analysis, a widely used technique to analyse “big data” where the number of subjects is far exceeded by the number of data points. Component analysis makes “big data” more manageable by representing numerous variables using a far smaller number of variables, usually 100- to 1000-fold fewer. How variables select a single variable to represent them varies, but it remains that using fewer representative variables can increase study power. Also, it can attenuate false-positive results driven by the reliance on single points of data which can suffer from known and unknown sources of error and bias [26]. Bias arising from genetic ancestry- or cohort-specific characteristics at individual sites of DNAm is particularly challenging in this field [5]. Component-based analysis also allowed us to leverage population-based data to assign a unique risk profile to each child that is “normalised” in the context of the whole population. In other words, the MRP positions each child relative to other children.

Much like the clinical observation of a patient, component-based analysis enables us to view prenatal risk exposure as multifactorial and existing on a continuous normal distribution. Since we link gwDNAm patterns to MRPs, inferences about DNAm are made relative to the whole population. This diminishes the risk of drawing statistically flashy results that only exist in the subset of individuals with DNAm data.

In part two of the study (Fig. 1, purple), we aim to model the reality of complex interactions between multiple biological inputs that lead to a given phenotype. We used decision tree-based analysis, a family of methods that gained popularity in medicine during the COVID-19 epidemic as a resource allocation tool to identify patients most likely to suffer serious morbidity or mortality. Decision tree-based methods complement a clinician’s intuitive integration of numerous patient features to draw a clinical conclusion. The goal is to discover patient features that are relevant to the patient’s outcome, regardless of their effect size. The decision tree structure enables it to detect non-linear and complex interactive effects that may reveal novel yet relevant relations. This ability helps to account for “atypical” individuals, where a feature that typically predicts one outcome is modified by other patient characteristics and leads to an alternate outcome.

Random forests (RFs) are a type of decision tree analysis that generates thousands of trees through an algorithm that randomly shuffles data values. The collation of these results is akin to the consensus of thousands of doctors who have each encountered thousands of unique patients. This inherent property of RF diminishes overfitting to a specific subset of a population. Further details on Parts 1 and 2 of our analysis are discussed below and in Additional file 1. All statistical analyses were performed using R software (version 4.0.0) [27]. Multiple testing corrections and power calculations are unsuitable for this study design since we are not conducting multiple independent tests or seeking differences between groups.

#### Part 1: Component-based analysis—connecting risk to molecular context using multidimensional data (Fig. 1, aqua)

We used component-based analysis to extract MRP-related DNAm patterns in three steps. We summarise these steps (1-A, 1-B, and 1-C) below.

##### Step 1-A: Generating population-based MRPs using factor analysis

We used the Factor Analysis for Mixed Data (FAMD) method, a principal component-based technique, as described in the *FactoMineR* R-package [28]. We refer to these mathematical representations of MRPs as “dimensions”, as the package authors did. We performed model-based missing value imputation with the built-in *imputeFAMD* function. The best-fit model included eight variables: birth weight, maternal smoking during pregnancy, household smoking, maternal grandfather’s history of smoking, maternal partner’s smoking history, maternal grandmother’s history of smoking, maternal history of smoking, and grandmother’s smoking while pregnant with mother, hereafter referred to as MRP-related variables (Table 2). The details regarding sampling adequacy, variance captured, and variable contributions are available in Additional file 1.

**Table 2:** Descriptive statistics by maternal smoking in pregnancy classification

	**Non-smoker**	**Non-sustained**	**Sustained**	**Unknown**	**Total**	***p* value**
**Total**	*N* = 8754	*N* = 963	*N* = 3713	*N* = 1781	*N* = 15,211	
**Sex**						0.022
N-Miss	22	1	7	487	517	
Male	4460 (51.1%)	476 (49.5%)	1987 (53.6%)	650 (50.2%)	7573 (51.5%)	
Female	4272 (48.9%)	486 (50.5%)	1719 (46.4%)	644 (49.8%)	7121 (48.5%)	
**Ethnicity**						< 0.001
N-Miss	165	21	122	683	991	
Caucasian	8166 (95.1%)	891 (94.6%)	3369 (93.8%)	981 (89.3%)	13,407 (94.3%)	
Other	423 (4.9%)	51 (5.4%)	222 (6.2%)	117 (10.7%)	813 (5.7%)	
**Gestational age**						< 0.001
N-Miss	2	0	1	589	592	
Mean (SD)	39.3 (2.4)	39.5 (2.1)	39.2 (2.5)	28.0 (13.8)	38.4 (5.5)	
Range	9.0—44.0	18.0—47.0	10.0—46.0	4.0—45.0	4.0—47.0	
**Birth weight, internal *z*-score**						< 0.001
N-Miss	162	12	80	793	1047	
Mean (SD)	0.0 (0.5)	0.1 (0.5)	-0.1 (0.5)	-0.1 (0.5)	0.0 (0.5)	
Range	-4.4	-3.9	-3.8	-3.8	-4.4	
**Birth length, internal *z*-score**						< 0.001
N-Miss	245	26	132	813	1216	
Mean (SD)	0.1 (1.6)	0.4 (1.6)	-0.3 (1.6)	-0.1 (1.4)	0.0 (1.6)	
Range	-14.8	-12	-13.7	-9.9	-14.8	
**Gestational weight gain**						< 0.001
N-Miss	1937	235	1148	1608	4928	
Over	1771 (26.0%)	277 (38.0%)	714 (27.8%)	48 (27.7%)	2810 (27.3%)	
Recommended	2749 (40.3%)	278 (38.2%)	912 (35.6%)	66 (38.2%)	4005 (38.9%)	
Under	2297 (33.7%)	173 (23.8%)	939 (36.6%)	59 (34.1%)	3468 (33.7%)	
**Maternal grandmother—ever smoked**						
N-Miss	455	29	232	1781	2497	
False	4280 (51.6%)	562 (60.2%)	2373 (68.2%)	0	7215 (56.7%)	
True	4019 (48.4%)	372 (39.8%)	1108 (31.8%)	0	5499 (43.3%)	
**Maternal grandmother—smoked while pregnant**						
N-Miss	496	32	245	1781	2554	
Don’t know	1178 (14.3%)	152 (16.3%)	514 (14.8%)	0	1844 (14.6%)	
False	5543 (67.1%)	610 (65.5%)	1709 (49.3%)	0	7862 (62.1%)	
True	1537 (18.6%)	169 (18.2%)	1245 (35.9%)	0	2951 (23.3%)	
**Maternal grandfather—ever smoked**						
N-Miss	297	37	396	1781	2511	
False	6383 (75.5%)	482 (52.1%)	1064 (32.1%)	0	7929 (62.4%)	
True	2074 (24.5%)	444 (47.9%)	2253 (67.9%)	0	4771 (37.6%)	
**Mother’s partner—smoked while pregnant**						
N-Miss	290	12	195	1781	2278	
False	8125 (96.0%)	863 (90.7%)	3032 (86.2%)	0	12,020 (92.9%)	
True	339 (4.0%)	88 (9.3%)	486 (13.8%)	0	913 (7.1%)	
**Others who smoke in household**						
N-Miss	554	45	342	1781	2722	
False	5992 (73.1%)	731 (79.6%)	2799 (83.0%)	0	9522 (76.2%)	
True	2208 (26.9%)	187 (20.4%)	572 (17.0%)	0	2967 (23.8%)	
**Maternal education level at time of pregnancy**						< 0.001
N-Miss	531	76	508	1534	2649	
Non-degree	7359 (89.5%)	763 (86.0%)	2464 (76.9%)	192 (77.7%)	10,778 (85.8%)	
Other	864 (10.5%)	124 (14.0%)	741 (23.1%)	55 (22.3%)	1784 (14.2%)	
**Maternal financial concerns**						< 0.001
N-Miss	755	105	618	1581	3059	
No strain	7445 (93.1%)	777 (90.6%)	2558 (82.6%)	163 (81.5%)	10,943 (90.1%)	
Other	554 (6.9%)	81 (9.4%)	537 (17.4%)	37 (18.5%)	1209 (9.9%)	
**Maternal psychopathology**						< 0.001
N-Miss	246	41	185	1580	2052	
Denies	6749 (79.3%)	644 (69.8%)	2188 (62.0%)	141 (70.1%)	9722 (73.9%)	
Other	1759 (20.7%)	278 (30.2%)	1340 (38.0%)	60 (29.9%)	3437 (26.1%)	
**Maternal substance use in pregnancy**						< 0.001
N-Miss	34	0	1	1403	1438	
Denies	8365 (95.9%)	914 (94.9%)	3366 (90.7%)	377 (99.7%)	13,022 (94.5%)	
Other	355 (4.1%)	49 (5.1%)	346 (9.3%)	1 (0.3%)	751 (5.5%)	
**Neighbourhood quality, ascending quality**						< 0.001
N-Miss	624	57	366	1170	2217	
4	387 (4.8%)	53 (5.8%)	250 (7.5%)	59 (9.7%)	749 (5.8%)	
5	331 (4.1%)	58 (6.4%)	251 (7.5%)	41 (6.7%)	681 (5.2%)	
6	617 (7.6%)	85 (9.4%)	359 (10.7%)	59 (9.7%)	1120 (8.6%)	
7	849 (10.4%)	83 (9.2%)	438 (13.1%)	64 (10.5%)	1434 (11.0%)	
8	1102 (13.6%)	113 (12.5%)	461 (13.8%)	92 (15.1%)	1768 (13.6%)	
9	1670 (20.5%)	200 (22.1%)	589 (17.6%)	90 (14.7%)	2549 (19.6%)	
10	1993 (24.5%)	185 (20.4%)	498 (14.9%)	104 (17.0%)	2780 (21.4%)	
11	872 (10.7%)	87 (9.6%)	247 (7.4%)	55 (9.0%)	1261 (9.7%)	
Other	309 (3.8%)	42 (4.6%)	254 (7.6%)	47 (7.7%)	652 (5.0%)	
**Maternal social status**						< 0.001
N-Miss	1822	243	1380	1651	5096	
2	2330 (33.6%)	203 (28.2%)	611 (26.2%)	36 (27.7%)	3180 (31.4%)	
3	3009 (43.4%)	318 (44.2%)	947 (40.6%)	52 (40.0%)	4326 (42.8%)	
Other	1593 (23.0%)	199 (27.6%)	775 (33.2%)	42 (32.3%)	2609 (25.8%)	
**Paternal social status**						< 0.001
N-Miss	1218	184	1136	1634	4172	
1	1053 (14.0%)	44 (5.6%)	100 (3.9%)	8 (5.4%)	1205 (10.9%)	
2	2790 (37.0%)	253 (32.5%)	662 (25.7%)	44 (29.9%)	3749 (34.0%)	
3	858 (11.4%)	101 (13.0%)	230 (8.9%)	10 (6.8%)	1199 (10.9%)	
4	2047 (27.2%)	268 (34.4%)	1089 (42.3%)	60 (40.8%)	3464 (31.4%)	
5	620 (8.2%)	86 (11.0%)	354 (13.7%)	18 (12.2%)	1078 (9.8%)	
Other	168 (2.2%)	27 (3.5%)	142 (5.5%)	7 (4.8%)	344 (3.1%)	

Columns 1–5 represent maternal smoking categories. Reported *p-*value: Continuous variables—ANOVA, categorical—chi-squared. “Miss”—missing data. Social status was derived from reported occupation according to the UK Registrar General’s classification. From 1 to 3, the occupations refer to manual unskilled, semi-skilled manual, and skilled occupations; 4 refers to skilled non-manual occupations; 5 refers to managerial and technical occupations; 6 refers to professional occupations; and 65 refers to armed forces. Education—“Degree” refers to having a university degree at the time of index pregnancy

##### Step 1-B: Training models of genome-wide patterns of DNAm related to MRPs using partial least squares (PLS) analysis

The MRPs served as “bait” to capture relevant DNAm patterns in cord blood using the *sPLS* function in the *sgPLS* R-package. The objective of PLS modelling is to predict MRP with DNAm data (depicted as Y and X, Additional file 2, Figure S1, inset). The PLS output represents sources of variability within the DNAm data using a small number of components that best represent MRPs. Components are extracted such that they are uncorrelated. In this paper, we refer to “components” as the mathematical representation of the molecular variation we refer to as gwDNAm patterns.

##### Step 1-C: Testing gwDNAm patterns on different data sets

The PLS weightings among CpG sites trained from our cord blood model were the “template” to test the same patterns in different DNAm data sets while blinded to their corresponding MRP data. We used the *predict* function of the *sgPLS* package to test the cord blood model on peripheral blood at ages 7 and 17 years in ARIES and perform external validation using cord blood from GenR.

#### Part 2: Tree-based analysis—finding robust relations between variables and outcomes in complex data structures (Fig. 1, purple)

The RF method can accommodate two important considerations: first, non-normal residual distributions found in omic and risk exposure data that violate traditional statistical assumptions and, second, indirect and/or non-linear relations between predictors and outcomes. RF uses an ensemble of decision trees derived from bootstrapped samples of the data set and random variable selection to optimise model stability and attenuate overfitting [29]. RF ranks the relevance of competing variables in predicting outcomes by assigning an importance score (calculated as the mean decrease in accuracy) [29]. Alluding to our earlier analogy about decision tree analysis, suppose those thousands of doctors “rated” how relevant a variable was to predict an outcome. The importance score represents a collation of these ratings, with a higher score representing a variable that was more helpful in deciding the outcome.

To ensure the robust selection of variables, we empirically evaluate what threshold of importance is considered relevant to predicting an outcome using a two-step RF strategy, [30] which is described briefly below.

##### Step 2-A: Identifying relevant predictors of outcomes using the Boruta algorithm

We used the Boruta algorithm (from R-package of the same name) to select all variables relevant to an outcome [31]. This is well-suited to our study, which aims to understand the mechanisms underlying a process. Instead of using prediction metrics, Boruta selects relevant variables by testing if their relation to the outcome exceeds that which could occur by chance. This is performed by generating shadow variables, which are shuffled copies of real variables such that their values no longer have any correlation with the outcome. Using a binomial test (*p* < 0.05), the model can estimate the likelihood a real variable will exceed the importance of a shadow variable. Thus, shadow variables introduce additional randomness to the model, making it more robust to random chance or correlations that can lead to false positives. We included the following previously associated covariates in all models: sex, maternal educational level, paternal social class, and in models using DNAm data, blood cell type proportions [32–34].

##### Step 2-B: Assessing the performance of trained RF models using Boruta-selected variables

We calculated model metrics using the *caret* R-package [35]. We calculated the performance metrics of the model using Boruta-selected variables (implemented in the *caret* R-package, [35] ntree = 500). We conducted re-sampling using fivefold cross-validation with 3 repeats and considered a combination of model performance metrics, namely mean squared error (MSE), mean absolute error, and coefficient of determination (*R*^2^). In this way, we compared the predictive performance of models using DNAm data, MRPs, and MRP-related variables. We further tested the performance of models using DNAm data from peripheral blood and GenR cord blood.

#### Confounder analysis

All models included sex, maternal education, and paternal social status as covariates. We selected the latter two variables among other social confounders after evaluation of multicollinearity and data loss due to missingness (see Additional file 1: Evaluating confounder effects). To understand the impact of confounders specifically on gwDNAm, we also performed an additional analysis using another component-based technique, singular value decomposition (SVD). SVD has been applied to identify covariation with confounding variables in DNAm data using R-package ChAMP [36]. Models of cardiometabolic outcomes included growth variables given the correlation between earlier and later time points in child developmental trajectories.

#### Mapping gwDNAm patterns to chromatin function and regulatory features

We performed enrichment-based analysis to evaluate the molecular relevance of gwDNAm patterns based on publicly available data as previously described (Additional file 1: Enrichment-based analysis—finding molecular relevance).

## Results

### Characteristics of discovery cohort

Among ALSPAC subjects, 15,454 newborns had at least one data item collected at birth. The construction of MRPs included data from these newborns and their mothers. DNAm in ARIES was measured from cord blood at birth and around ages 7 (mean 7.5, standard deviation (SD) 0.1) and 17 (mean 17.1, SD 1) years in 914, 973, and 974 children, respectively. Previous studies comparing the baseline characteristics of families in the ALSPAC versus the ARIES datasets found that the ARIES mothers were older, reported less maternal smoking, attained a higher educational level, and were more likely employed in non-manual labour [24, 37]. Characteristics of MRP-related variables are tabulated in Table 2.

### Population-based maternal risk profiles and gwDNAm patterns

We performed factor analysis to represent the MRP-related variables as a composite score (see the “Methods” section—Part 1, Step 1-A). Based on model metrics, the top five dimensions were selected (scree plot in Additional file 2, Figure S1). Each dimension is a mathematical representation of the MRPs and describes a varying degree of contribution from all constituent MRP-related variables (see Table 2). Thus, children in the entire ALSPAC data set were assigned a score for each dimension, giving each individual a unique five-part profile of risk exposure.

**Fig. 2:**
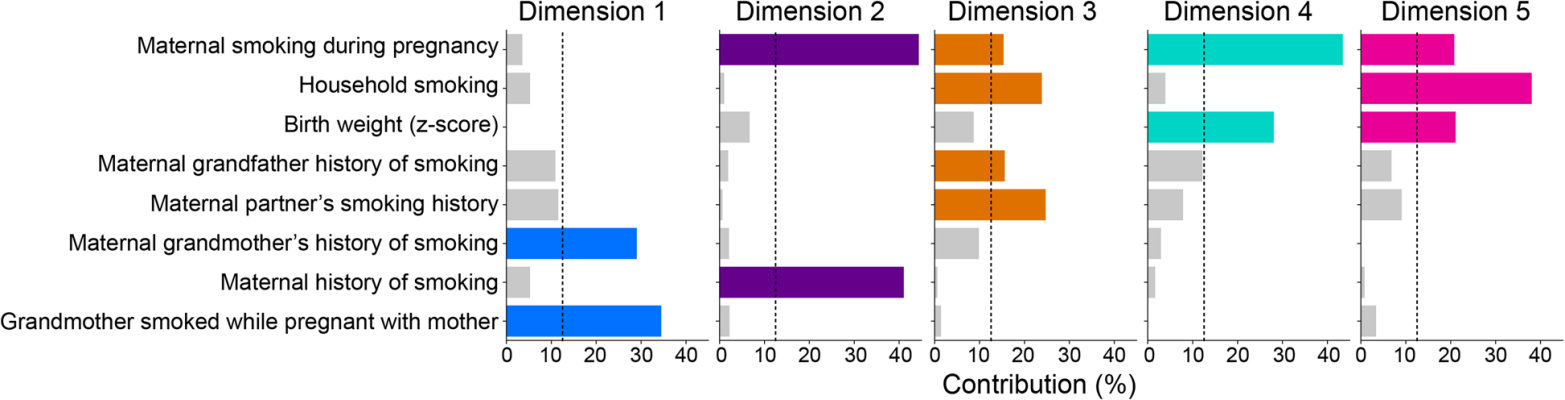
Bar graph of the relative contribution of variables (x-axis) to maternal risk profile construction. While each risk profile has varying contributions from all eight risk-related variables, certain variables dominate the construction of a dimension. The bars represent the relative contribution of each variable to a given dimension. Contributions that exceed the grey vertical line (calculated as in [20]) belong to variables that are considered the most representative of a dimension (see Additional file 1). Colours show variables that exceed this threshold and will be used to refer to each dimension in subsequent figures

Each of the first two dimensions captured nearly 20% of total data variability. We can evaluate the risk represented by each dimension by viewing its most prominent contributory variables (Fig. 2). For example, Dimension 1 primarily captures the infant’s grandmaternal data, i.e. the grandmother’s smoking history and whether the grandmother smoked while pregnant with the mother. In contrast, Dimension 2 captures similar information for the infant’s mother.

While we refer to the MRP representations as “dimensions”, we use the term “components” to refer to gwDNAm patterns generated by the PLS method (see the “Methods” section—Part 1, Step 1-B). Each component mathematically represents a consistent pattern of DNAm at specific genomic sites that are related to MRP variability among subjects. PLS does not seek to account for the most variability in data but the most shared variance between two sets of data. In this way, PLS assigns component scores to each person that indicates how alike or unlike his/her DNAm variability is to that given component pattern, which in turn is related to MRP. Thus, every subject has a unique identity of gwDNAm that is related to his/her risk exposure. In Additional file 3, we provide the full list of CpGs most representative of each gwDNAm pattern.

### gwDNAm patterns in the context of confounders and population-based MRPs

Inherent in the technical measurement of biology is the overlap of variability between the variable of interest and confounders (see the “Methods” section—Confounder analysis). To identify the degree of overlap, we evaluated the correlation between components with confounders (Additional file 2: Figures S2-S5). There was strong evidence of relation to sex with Components 1 to 3, 5, 6, and 10. None showed strong relations to maternal education or paternal social status. The strongest correlations to cell type proportions were between Components 1 and 3 with granulocytes and CD4 cells and Component 6 with nucleated red blood cells.

**Fig. 3:**
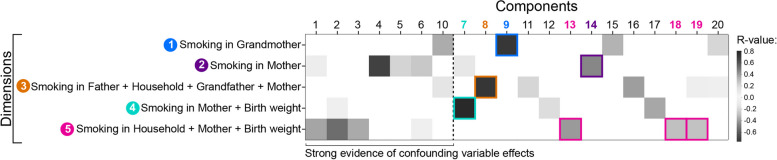
Correlation matrix of DNAm components (methylation patterns) and dimensions (MRPs). The mathematical representations of the MRPs are “dimensions”, and the gwDNAm patterns are “components”. Components 1–6 and 10 are strongly associated with confounders. We are unable to separate the biological variability of interest from that due to strong confounding in these components; thus, they will be dismissed. (As the sign of the correlation coefficient does not change the interpretation, it has been omitted for figure clarity)

Next, we show the relation between MRPs and gwDNAm patterns in Fig. 3. We observed that Dimension 1 (grandmaternal smoking features) correlated most with the DNAm patterns represented by Component 9. Dimension 2 (maternal smoking features) was most related to Component 14. Similarly, we observed the relation between Dimension 3 (smoking in mother/father/grandfather/other household members and infant birth weight) to Component 8, Dimension 4 (maternal smoking and birth weight) to Component 7, and Dimension 5 (smoking in mother/other household members and birth weight) to Components 13, 18, and 19. For the sake of brevity and interpretability, we will focus on these components and exclude discussion of confounder-related components (Components 1–6 and 10) in the “Results” section moving forward.

### gwDNA patterns at birth perform comparably to risk variables to predict outcomes

In Step 2-A, we evaluate if gwDNAm patterns relate to child outcomes compared to the risk variables from which they were derived. For each outcome, we compared three models using *R*^2^ and MSE: Model 1—individual MRP-related variables, Model 2—MRP dimensions, and Model 3—MRP-based gwDNAm patterns.

Each model included confounders (see the “Methods” section—Confounder analysis) and shadow variables. Shadow variables are representations of predictor-outcome relations due to random chance (see Additional file 1: Tree-based analysis). Model 3 also includes cell count proportion estimates.

**Fig. 4:**
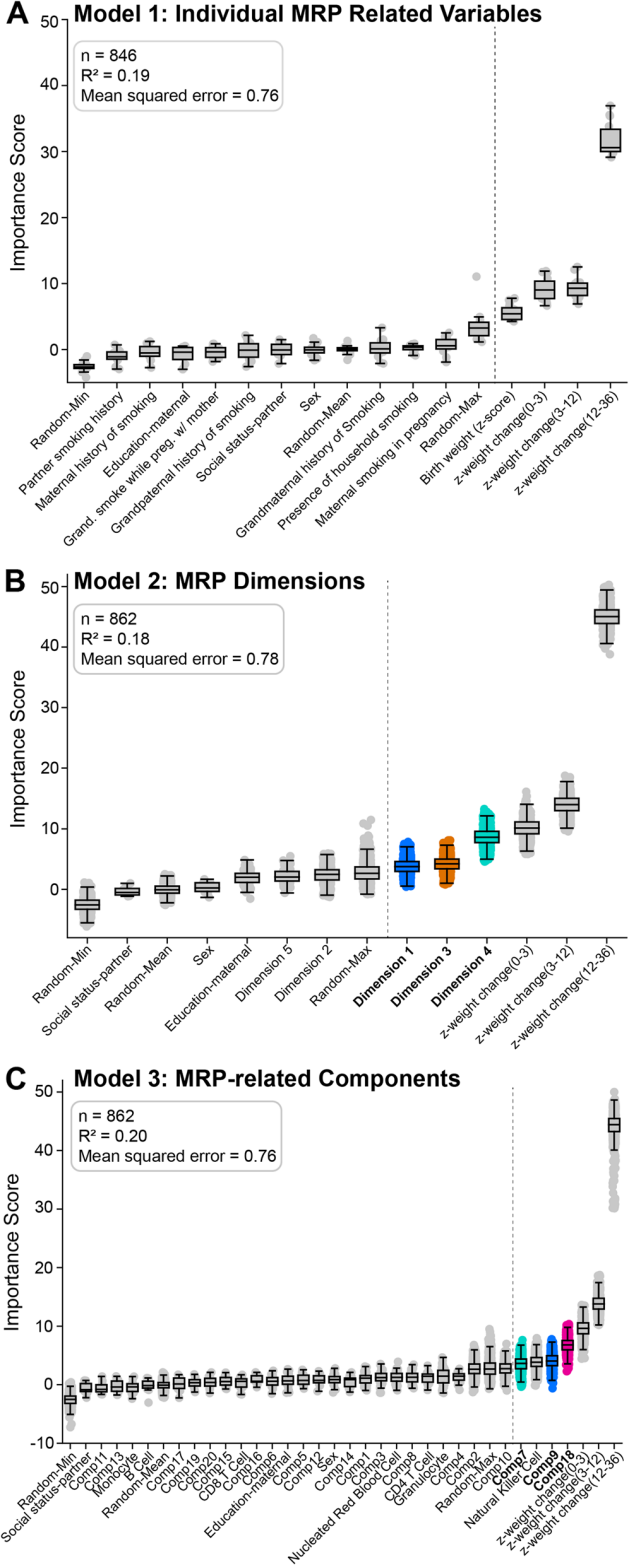
Comparison of models using DNAm patterns versus clinical variables using RF. Boxplot of importance scores (*y*-axis) of variables (*x*-axis) predicting waist circumference at age 10 using three different models (panels **A**–**C**).** A** Model 1 using individual MRP-related variables. **B** Model 2 using MRP dimensions from factor analysis. **C** Model 3 using gwDNAm patterns in cord blood. The *x*-axis includes additional control variables of estimated blood cell type proportions. **Inset**: Sample size and performance metrics for each model. Boxes appearing to the right of the grey vertical line (i.e. after the maximum randomised shadow variable) indicate relevant variables. The box and whiskers display the distribution of permutation values and cannot be used to tell statistical significance [31]. Blue—Dimension 1 (smoking in grandmother), orange—Dimension 3 (smoking in father + household + grandfather + mother), green—Dimension 4 (smoking in mother + birth weight), pink—Dimension 5 (smoking in household + mother + birth weight)

Figure 4 shows these models for waist circumference (internal* z*-score) at age 10. Early growth rates (i.e. before age 36 months) were the top-ranked variables in all three models. In Model 1, we observed that smoking-related variables were not ranked as relevant. This was consistent with traditional linear models (for example, the linear effect of maternal smoking for this outcome was *β* =  − 0.09, *p* = 0.58). This was the general observation across various outcomes, except for two academic performance measures (see Additional file 2: Table S3). Model 1 always had a smaller sample size, largely due to missing self-reported maternal smoking data*.*

We observed that Dimension 4 and Component 7 (green boxes), as well as Dimension 1 and Component 9 (blue boxes), were selected in Models 2 and 3, respectively. These Dimension/Component pairs are correlated in Fig. 3. While their shared variability may explain the selection of these pairs, we also observed that Dimension 3 was selected in Model 2, but its correlate, Component 8, was not selected in Model 3. Component 18 was selected, but not Dimension 5. This could reflect differences in Dimension/Component correlations. However, it also suggests that the molecular patterns may hold variability that is distinct from the risk profile from which they were generated, thus changing the association to the same phenotype.

In Step 2-B, we compared the performances between Models 1, 2, and 3. The differences between the three models were minor (Fig. 4—inset), supporting the notion that the DNAm components model (Model 3) comparably captures the relation to subject outcomes.

### gwDNAm patterns at birth relate to future cardiometabolic outcomes

We first review results from Model 3 using DNAm data collected at birth from cord blood. Component 9 was one of the most consistently relevant components among cardiometabolic outcomes (Additional file 2: Figs. S6-S9). This component was selected in the model of waist circumference at age 10 (Fig. 4C) and in models of blood pressure and lean mass at various ages. It was not related to weight at any age. Component 7 was related to lean mass, waist circumference, and weight at multiple ages. Component 18 demonstrated similar results for waist circumference, weight, blood pressure, and fat mass. Components 8 and 14 were related to a few outcomes at certain ages.

### gwDNAm patterns at birth relate to future neurocognitive outcomes

We observed that Components 9, 18, and 19 were related to parent-rated development at 6 months. Components 11, 13, and 19 were related to academic performance at various ages from about age 5 to 14. Component 19 was also related to intelligence assessed at 8 years (Additional file 2: Figures S10-S12, respectively). Neurocognitive outcomes generally had more missing data than cardiometabolic ones. For instance, 87% of subjects were missing data on intelligence measured at 4 years. Models for this outcome were very poor and did not generate stable predictors. Nearly all models selected paternal social status, which is consistent with other reports in this data set [38, 39]. This was also observed in Model 1. This consistency among certain variables is much like the behaviour of early growth variables in the cardiometabolic models.

### Sensitivity analysis

Like all RF algorithms, Boruta does not accept missing values. Discarding incomplete observations has two important effects. It diminishes the sample size, which decreases the power to detect relevant variables. It also alters outlier and data noise effects on model specification. Thus, we performed a sensitivity analysis of RF models using DNAm data to compare three models: (1) Raw—using all variables and only complete (i.e. no missing data) observations; (2) Boruta—using only Boruta selected variables and only complete observations; and (3) Imputed—using all variables and all observations (i.e. missing data was imputed). As an example, Additional file 2: Table S1 shows how these three models compared using waist circumference at age 10. We observed little difference in error rates between the models among outcomes. Data for other outcomes is available upon request.

### gwDNAm patterns generalise over time and across populations

We examined whether DNAm patterns could be observed in children as they aged and in another cohort. Previous literature has shown that complex trait-related DNAm variability in cord blood has small effect sizes in the 1–2% range [40], a level that can easily be dwarfed by physiological, stochastic, and dataset-specific variability over time [41, 42]. We reasoned that if DNAm patterns are not biologically related to future phenotypes, then there would be little to no detectable relation between the two in different DNAm datasets. We tested the DNAm patterns trained on ARIES cord blood samples on DNAm data collected from (a) blood samples from ARIES subjects at ages 7 and 17 years and (b) cord blood samples from GenR.

#### Testing in different data within the ARIES cohort—peripheral blood samples in later childhood

**Fig. 5:**
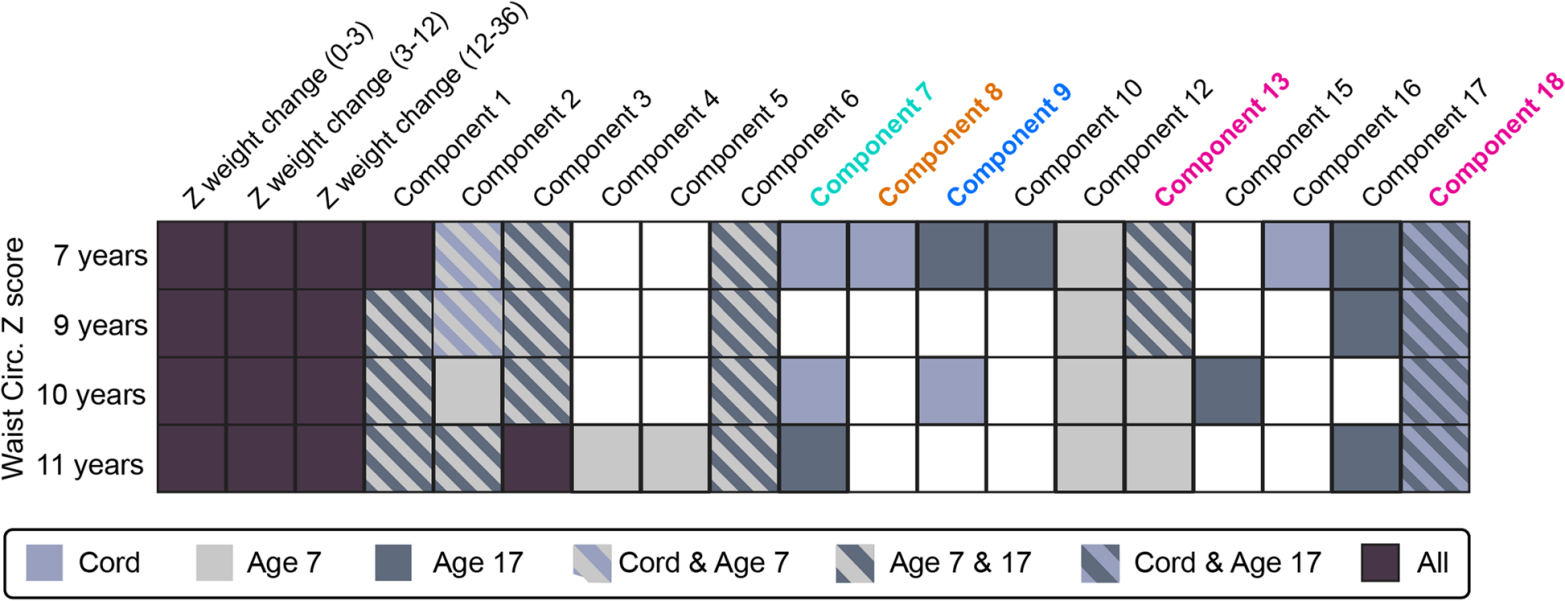
Summary of DNA methylation components relevant to waist circumference (*z*-score by sex) measured at ages 7, 9, 10, and 11 years (rows). Only components ranked as relevant at least once for this outcome are included. The legend shows the ages at which DNA methylation data was collected. The coloured text of component axis labels corresponds to the related dimension, as seen in Fig. 2. The results of Components 1–6 and 10 are not discussed as they demonstrate strong relations with confounders. The boxes with hashes indicate components that demonstrated the same relation to phenotype in DNAm data collected at more than one age. In other words, these hashed boxes indicate relations that persisted over time. Looking down the columns, we see that half the components that were related to waist circumference measured at a younger age would continue this association to age 11 years (i.e. coloured boxes are stacked vertically together). If the relation between DNAm components and future phenotype were due to chance or technical artefacts, this consistency as the phenotypic and molecular clocks ticked would be less likely. Results for other outcomes are in Additional file 2

The performance of models using DNAm data at older ages was similar compared to those using cord blood (Additional file 2: Figure S13 and Table S2). A subset of patterns persisted in their association with outcomes. For example, waist circumference was related to Component 18 in both cord blood and blood at age 17 years (Fig. 5). Blood pressure was related to Components 9 and 18 in blood samples at all three ages (Additional file 2: Figure S6). Similarly, Components 11, 13, and 19 were related to school performance at older ages (Additional file 2: Figure S10).

Some associations with outcomes only appeared in DNAm in later childhood. For example, fat mass measured at ages 9, 11, and 13 years were all related to Component 9 but only in blood collected at age 7 (Additional file 2: Figure S7). Samples sizes for DNAm data in later childhood were larger as approximately 60 “new” subjects were added [24, 43] compared to the cord blood data. This gives us the advantage of having more testing data than training data and more power to detect associations.

We also point out that testing the relation to outcomes that precede DNAm data collection cannot support “prediction” in the same way as our findings from cord blood. Because most neurodevelopmental outcomes available for this study were measured in early childhood, only school performance has data collected prospectively or concurrently in relation to DNAm data collection. It is possible that a neurodevelopmental trait is reinforcing or generating the DNAm pattern, i.e. reversal causality. For example, the Denver developmental screen was last collected at 30 months of age, but the relation of Component 11 to these outcomes was only observed at ages 7 and 17.

#### External validation in GenR cohort—cord blood samples

**Fig. 6:**
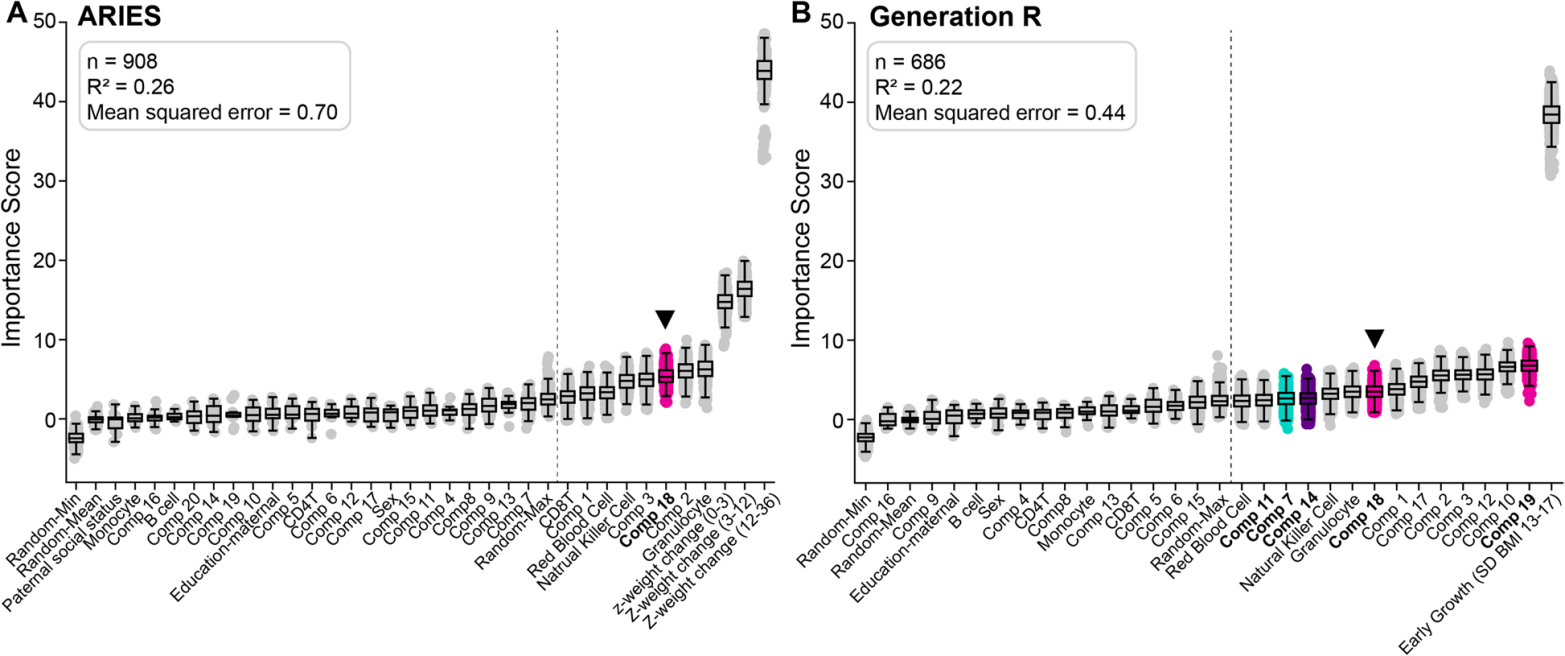
External validation of DNAm patterns. Boxplots of importance scores (*y*-axis) of variables (*x*-axis) predicting body mass index (BMI) in early childhood in the discovery cohort (ARIES) (**A**) versus independent replication cohort (Generation R) (**B**). Component 18 was selected in both cohorts. The replication cohort in **B** also ranked three other DNAm components that frequently showed relations to various other cardiometabolic outcomes (bold *x*-axis labels, Components 7, 11, and 19). Boxes appearing to the right of the grey vertical line (i.e. after the maximum randomised shadow variable) indicate relevant variables. The box and whiskers show the distribution of permutation values and cannot be used to tell statistical significance [31]. Purple—Dimension 2 (smoking in mother), green—Dimension 4 (smoking in mother + birth weight), pink—Dimension 5 (smoking in mother + household + birth weight). **Inset**: Sample size and performance metrics for each model. **A** Discovery Model from ARIES data using MRP DNAm patterns from cord blood to predict BMI (*z*-score by sex) at age 7 years. The *x*-axis includes the same control variables as in model 3 of Fig. 4C. **B** Validation Model from GenR data using MRP DNAm patterns in cord blood samples to predict BMI (*z*-score by sex) at age 6 years. The *x*-axis includes estimated cell type counts, sex, maternal education, and early growth (represented by BMI *z*-score measured between 13 and 17 months of age)

We performed validation using 686 cord blood DNAm samples from GenR to predict body mass index (BMI) at age 6 years (Fig. 6). We compared this model to that from ARIES cord blood data and BMI at age 7 years. As in ARIES, early growth and components related to Dimension 4 (MRP representing smoking in the mother and household members and birth weight) were selected as relevant predictors in the GenR model (Fig. 6, data points in pink, Components 18 and 19). Components 7 and 11 (axis labels in bold in Fig. 6) were also selected. These components were relevant to several cardiometabolic outcomes in ARIES (see Fig. 3C for waist circumference and Additional file 2: Figure S6-S9 for weight, blood pressure, and fat and lean mass).

The GenR model had comparable performance metrics compared to other cardiometabolic outcome models using ARIES data (see Fig. 6 (inset) and Additional file 2: Table S2).

### gwDNAm patterns have molecular features that enable them to perform together as a functional unit

We took a more detailed look at the genomic localisation of gwDNAm patterns. PLS enables the empirical selection of DNAm sites that most consistently and strongly represent MRPs (described and schematically depicted in Additional file 1: Figure S3). This selection process adheres to our objective to view risk-related covariation in methylation as a coordinated regulatory event with *interdependence* between sites across the genome. This contrasts with *individually* testing associations between risk exposure and methylation (either as single or clusters of sites) in thousands of post hoc multiple hypothesis tests, such as performed in an epigenome-wide association study (EWAS).

#### gwDNAm patterns are positioned preferentially in regions of open chromatin and gene regulatory sites

**Fig. 7:**
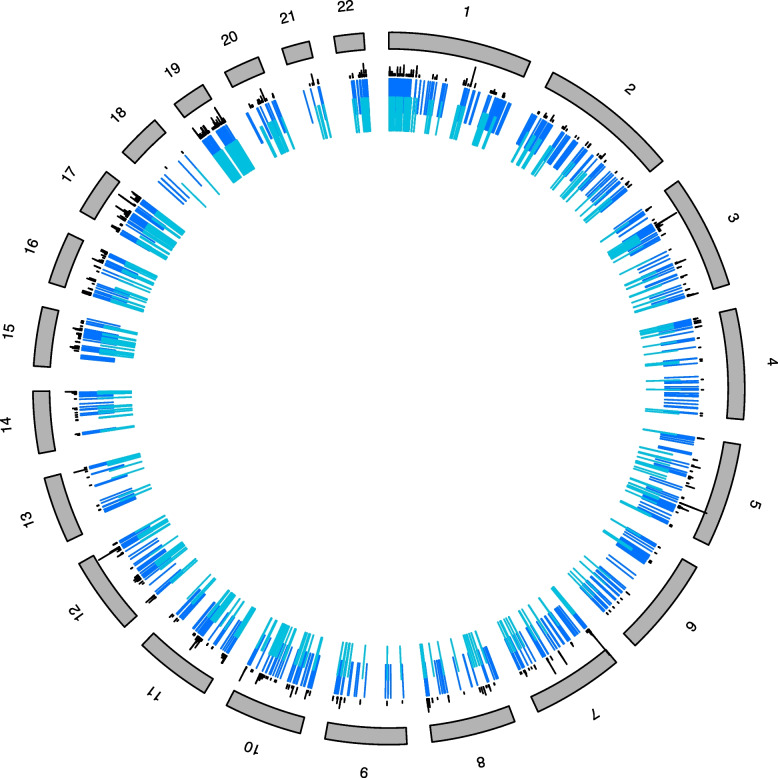
Genomic organisation of DNAm sites representative of a DNAm pattern within the chromatin environment. We show Component 9 (related to Dimension 1—grandmaternal smoking) as an example. The perimeter ring shows the ideogram of the human autosomes with chromosome numbers. Unlike differentially methylated regions, which typically describe contiguous locations, genomic sites representing gwDNAm patterns (dark blue lines) are spread across a given chromosome and across a cell’s chromosomes. This is consistent with the finding that methylation related to complex traits is a genome-wide event. It is also in keeping with the interaction of non-contiguous domains of DNA by their physical proximation in 3D space through chromatin looping structures and promoter-anchored interaction sites (black lines). Regulatory mechanisms tend to occupy open chromatin areas. Such areas are marked by DNase I hypersensitivity sites (light blue lines). The three different coloured lines appear to populate together, much like how cities would concentrate the machinery required to “trade” information regarding foetal exposures across the genome, changing the global fate of the cell

We tested whether gwDNAm patterns co-localise with sites involved in the functional regulation of chromatin output. We used two features: (1) DNase I hypersensitivity sites (DHSs) and (2) promoter-anchored chromatin interaction (PAI) and chromatin loop sites. DNAm locations that spatially intersect with these two features are positioned in a 3D chromatin environment that is open and conformationally poised to interact with gene regulatory factors. Sites representative of gwDNAm patterns were enriched for DHSs and PAI/loop features (permutation *p* < 0.01 for both, Additional file 2: Figure S14). Figure 7 visualises the non-random organisation of DNAm sites (dark blue lines) within a gwDNAm pattern across the chromosomes in intersection with these 3D chromatin features (light blue and black lines).

#### gwDNAm patterns have comparatively distinct overlap with molecular features and confounding variables

##### gwDNAm patterns demonstrate affinity for sites of transcription factor binding

To assess the functional relevance of EWAS candidates, most studies will test whether DNAm candidates are related to a gene product (usually from a coding gene that is linked to a certain mRNA, protein, or biological function like “inflammation”). Given that the 450K BeadChip was designed to interrogate DNAm at well-annotated genes, enrichment in such annotations would be unsurprising. We wondered whether the enrichments observed were simply due to the bias of the 450K BeadChip for certain genes, particularly genes that could demonstrate smoking-related changes but may have no biological effect on future health.

We challenged this query by testing whether genomic sites of a given gwDNAm pattern would still show greater than expected enrichment when directly compared to candidates identified by the largest-to-date meta-analysis of the association between sustained maternal smoking and cord blood DNAm (meta-EWAS) using the 450K BeadChip [44]. This study analysed associations across 13 different cohorts (*n* = 6685). The study team identified 2965 CpGs after correcting for multiple hypothesis testing, and 89% of these were associated with gene expression levels.

We used the XSTREME (meme-suite.org) analysis tool to evaluate enrichment in genomic sequences, called motifs, that exhibit affinity for binding to transcription factors. An enrichment is identified if the interrogated sites (in our case, sites representing a given gwDNAm pattern) can generate the same or more motifs of equal width and number of occurrences as the background sites (meaning meta-EWAS sites) based on log-likelihood ratios [45]. Most of the top 5 ranked motifs were novel (Additional file 2: Figure S15-S17). These novel motifs provide “an unbiased view of the in vivo DNA-binding propensities” [45] that represent possible novel or under-characterised regulators in referenced databases. Discarding the overlap with meta-EWAS candidates that were both large in number and closely associated with gene expression, sites representative of gwDNAm patterns were still localised to novel areas with an affinity for transcription regulation. In other words, the functional implications embedded within the physical distribution of DNAm patterns across the genome surpass what would be expected by chance or microarray-based bias.

##### gwDNAm patterns are enriched in tissue-specific molecular features

**Fig. 8:**
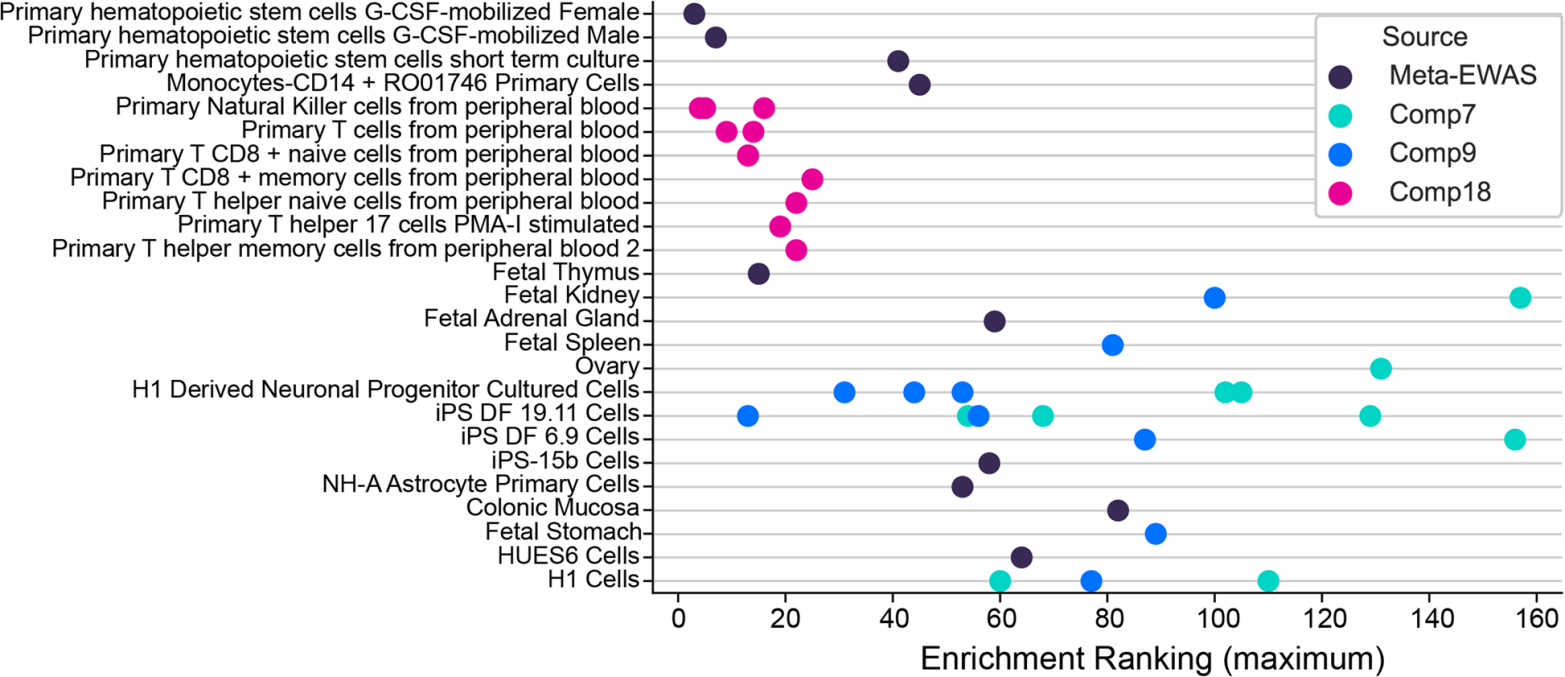
Chromatin mark enrichment analysis using LOLA. *X*-axis: Enrichment rank where lower numbers indicate higher rank and thus stronger evidence of enrichment. Colours represent the source of DNAm candidates. Tissue/cell cultures demonstrating a specific chromatin mark are labelled on the* y*-axis. Cells sharing embryonic lineage are grouped together where applicable. Sites from a given DNAm pattern (i.e. same colour circles) tend to have two or more clusters with common lineages, as represented by circles falling on the same or nearby horizontal lines. In contrast, meta-EWAS candidates [44] represented by dark purple circles appear scattered across unrelated lineages. Granulocyte colony-stimulating factor, G-CSF; phorbol myristate acetate–ionomycin, PMA-I; human embryonic stem cell line, H1; induced pluripotent stem line derived from foreskin fibroblasts, iPS DF; human embryonic stem cell line, HUES

We used locus overlap enrichment analysis (LOLA) and the curated LOLA database to determine whether gwDNAm patterns localised to tissue-specific chromatin marks [46]. gwDNAm patterns were enriched (*q*-value < 0.05) by chromatin marks specific to certain cell lineages, often with multiple enrichment clusters falling on the same or embryologically-related tissues (Fig. 8). For instance, Component 18 is dominated by high-ranking clusters specific to white blood cells. Component 9 is characterised by high-ranking clusters around two main panels: neuronal progenitor and iPS DF 19.11 (induced pluripotent stem cells derived from foreskin fibroblasts) cells, both arising from the neuroectodermal cell lineage. A similar enrichment in this cell lineage was observed in Component 19 (data not shown). In contrast, the meta-EWAS candidates harboured chromatin marks that were widely spread across cell lineages with no high-ranked clusters on specific cell lineages or marks from the same cell panels. These findings were consistent when using another tissue-specific molecular marker, DHSs [47] (Additional file 2: Figure S18).

##### gwDNAm patterns have distinct relations to confounders compared to previous candidates of foetal exposure to smoking

We first examined the overlap of our DNAm component candidates to the 28 maternal smoking-related CpG sites in cord blood identified in the same ARIES dataset by Richmond et al. [48]. Seven of our DNAm components overlapped with at least one of their hits (Additional file 2: Figure S20). Three of these components were related to confounders (Components 1 and 4 are related to sex, while Component 6 is related to sex and cell count). All overlaps had an associated target gene except for one (cg04598670).

**Fig. 9:**
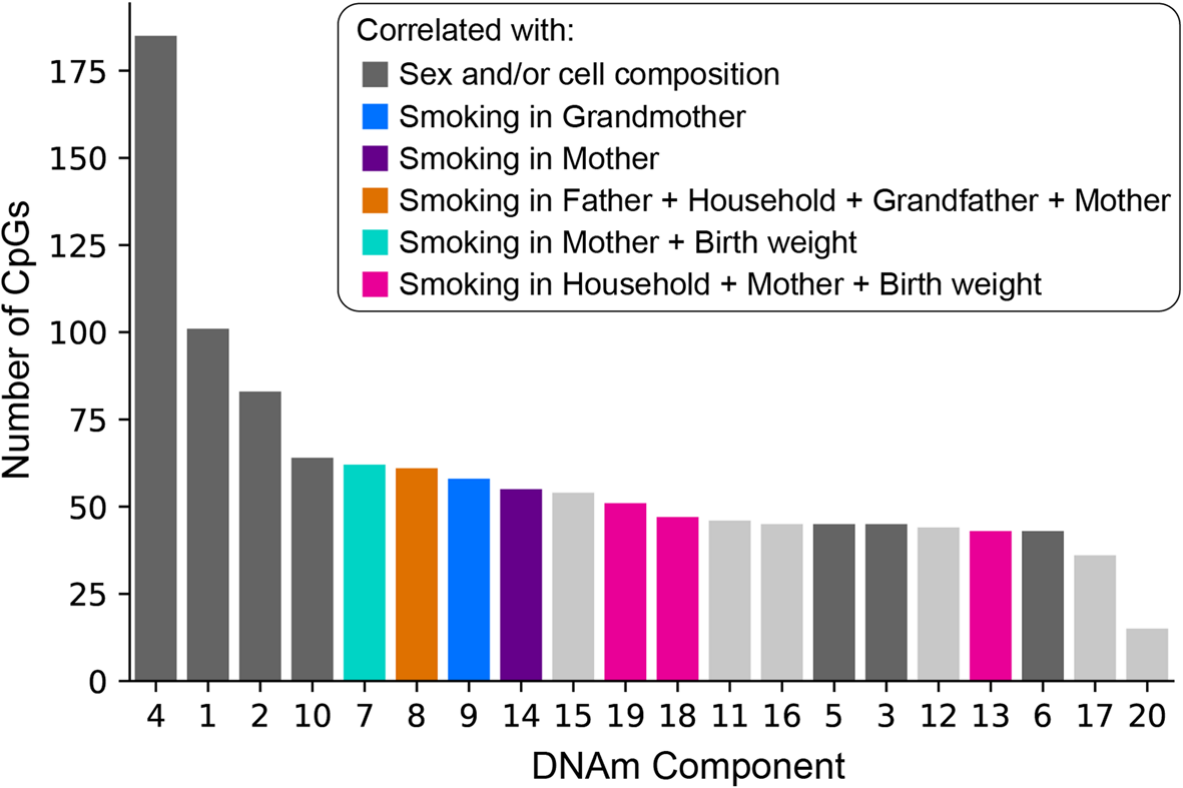
Overlap of gwDNA methylation patterns with meta-EWAS candidates [44]. Histogram shows the number of EWAS candidates that match sites within DNAm components (patterns). The dark grey bars highlight seven DNAm components that we dismissed because they relate strongly with the confounders of sex and/or cell composition. Components related to confounders were also found to be frequently related to health outcomes. Among 1183 overlaps, 566 overlapped with these components (dark grey bars), 749 represented unique CpG sites, and 474 localised to unique genes. A large proportion of gwDNAm pattern representative sites “consistent” with this EWAS were related to Component 4. Recall that this component was related to subject sex and was the most strongly correlated component with the maternal smoking-related MRP, Dimension 2

Confounding from covariates like sex and cell count affect DNAm data distributions differently and can be cohort-specific [49, 50]. Most of the EWAS hits discovered by Richmond et al*.* overlap with EWAS hits in other cohorts [4, 51–53]. Therefore, we looked at the meta-EWAS to examine if this was specific to the ARIES dataset. Forty-eight percent (566/1183) of overlapping sites coincided with components related to sex and/or cell count confounders (Fig. 9).

## Discussion

The rise in NCD prevalence and the resources needed to treat their lifelong complications are draining to health systems and societies. Current detection methods require an adequate degree of cellular damage to indicate entry into the diseased state. The most cost-effective means of stemming this global crisis is detection that is generalisable to the whole population yet targets intervention before disease onset. We believe the key to achieving this feat is by considering the complex and context-specific nature of NCDs.

In this study, we adopted a view of the early origins of NCDs where (1) foetal risk exposure is a multidimensional and population-normalised entity and (2) gene regulation is a transformer of cell fate that requires numerous coordinated changes across the genome. We believe this is the first identification of patterns of DNAm underlying population-based risk profiles. We employed component-based analysis and model-based imputation to lessen the loss and/or distortion of information due to reliance on single points of data from subjects or variables alike. Importantly, this can reduce the impact of error and/or bias from subpopulation-specific characteristics [26, 54, 55].

As a proof-of-concept, we used MRPs that modelled the risks surrounding why and how mothers smoke while pregnant. We used this constellation of risks to reveal how early experiences influence the root of health during foetal development, branching into tissues and organs as phenotypic offshoots after birth, as hypothesised in DOHaD theory [56–58]. This may account for how NCDs associated with prenatal smoke exposure have a wide breadth of effects, such as respiratory, cardiometabolic, cognitive, and psychological morbidities [10, 11, 13, 15]. Pleiotropy is when a single gene contributes to more than one unrelated phenotypic trait, and it occurs frequently in NCDs [59, 60]. If an isolated genomic area can exhibit such pleiotropy, then this may also be true of epigenetic differences related to exposures [59, 61, 62]. Accordingly, we anticipated that gwDNAm patterns would be linked to both physical and neurodevelopmental outcomes.

Population-based gwDNAm patterns can help capture biological heterogeneity that comes from the interaction of genetic, environmental, and random effects. Decision tree-based analysis has the ability to account for these interactions, revealing relations that are difficult to detect using traditional linear models. This facilitates a more realistic view of interactive, robust, and prospective relations between gwDNAm patterns and future cardiometabolic and neurocognitive outcomes. We tested these models in DNAm data from mid- and late childhood and in an independent cohort. It was remarkable to observe the persistent association of DNAm to child phenotypes regardless of data influences such as developmental maturation, technical artefacts, and data set-specific variability [40].

Previous perinatal DNAm studies that have attempted a multifactorial approach focused on maternal psychosocial stress [63–68]. A study by Laubach et al. used a composite score of socioeconomic status using maternal factors collected in Project Viva, a multi-ethnic pregnancy cohort started in 1999 [65]. In an EWAS using the 450K BeadChip, they identified three CpGs in cord blood that were associated with low versus high socioeconomic scores. Only one was replicated at age 3, and none were replicated at age 7 years. We are unaware of any attempt to link these findings to phenotypes. A recent study by Provenzi et al. used the average score on six retrospective questions related to maternal stress due to COVID-19 infection [67]. These researchers only evaluated the methylation status of the *SLC6A4* gene. They first found an association between COVID-19-related stress and methylation and, in a subsequent model, found the latter associated with only one out of three measured factors of infant behaviour. Most other studies use retrospective data or cannot capture foetal effects because of risk assessment or tissue collection at later ages (for a comprehensive review, see [69]).

Our aim to view each subject using a multidimensional and unique perspective could be described as “self-defeating” if judged by traditional statistical metrics. For instance, the PLS methodology seeks shared information between two datasets and does not maximise the variability explained. This directly undermines *R*^2^ and related metrics. Moreover, by avoiding the assumption that individuals fit into homogeneous categories, we cannot enhance the reported differential effect between groups. The non-linear and interactive effects detected by decision tree analysis are difficult to translate into straightforward results of causal, mediating, or dose–response effects, which are more familiar to clinicians, patients, and researchers [70].

DNAm data is also expensive and more invasive than routine history and physical measurements. However, we believe the value of gwDNAm patterns lies in how they may connect to real-life biological context. For example, the genome-wide distribution of these patterns intersects with various features that are integral to interactions with regulatory elements (e.g. locations associated with DHS, PAI, and transcription factor binding) and the 3D topology of chromatin (e.g. chromatin loop domains). This suggests that gwDNAm patterns have a spatial organisation linked to the functional units of cell fate decisions [71–74]. This is critical to operationalise one-dimensional measures of methylation into realistic reflections of the multidimensional and interactive nature of mechanisms underlying complex traits. Data supports that these chromatin features are key players in exposure-mediated shifts in gene expression and overall cell function [75]. This may also add to the potential forecasting ability of DNAm, given that the 3D re-organisation of chromatin can precede changes in gene expression [6, 73, 76].

It is possible that genotype-dependent methylation is responsible for our findings. However, we note that each gwDNAm pattern uses the magnitude and directionality of methylation at a given site *relative* to all other CpG sites across the genome to uncover consistent and coordinated patterns among individuals. Thus, DNAm sites within a given pattern are inherently related by design. Genotype-dependent methylation must act in magnitude and direction synchronously within gwDNAm patterns to give consistent false positive associations. If this biased the extraction of MRP-related gwDNAm patterns, then this same bias must also align itself to relate to outcomes across data sets spanning time, populations, and cardiometabolic and neurocognitive phenotypes. If that were the case, we reason that there should be far more consistent relations between differential methylation and child outcomes in the current literature [40].

CpGs representing a gwDNAm pattern are also conceptually different from differential DNAm sites identified in EWAS research because the latter have no innate relational organisation. This connectivity of sites *within* patterns reveals differences *between* patterns. We speculate these differences may underlie distinct processes such as cell- or stimuli-specific biological signalling. This was suggested by the degree of specificity in DNAm patterns, such as for certain tissues (Fig. 8) and for sources of variability due to blood cell type composition and sex (Fig. 9—dark bars). For example, eight meta-EWAS sites that annotate to *CYP1A1* overlapped with Component 4 (which is related to sex and the maternal smoking-related MRP, Dimension 2) and with Components 9 and 11 (which were related to child outcomes). A similar scenario was observed for sites annotated to *GFI1* and *AHRR* genes*.* One interpretation of these observations is that DNAm at these sites is confounded and should be discarded. However, we speculate that methylation (like for *CYP1A1*) is relevant to clinical outcomes depending on their interactions with genetic and epigenetic mechanisms at other sites that will vary in each individual. As such, there are instances where differential methylation at these same sites may be driven by “bystanders”, such as sex or cell composition that may covary or even interact with the exposure but do not lie at the biological root underpinning the phenotype. In other words, there may be specificity in the function of DNA methylation based on a broad scope of clinical and, thus, molecular contexts.

Previous literature has identified thousands of maternal smoking-related DNAm sites, many with theoretically plausible links to biological phenotypes. Compared to these (as represented by meta-EWAS data in our analysis), gwDNAm patterns were implicated in novel genomic sites (Fig. 9) and transcription networks (Additional file 2: Figures S15-S17) with a greater proportion of sites found in CpG poor or non-genic regions [77] (Additional file 3). Earlier studies have focused on candidates located around well-annotated protein-coding genes and biological pathways. This leads to a gene-centric bias, which is potentially harmful given that it may neglect lesser-known but potentially important mechanisms [78]. Mounting evidence suggests that the non-coding genome has a major role in human disease [79–81].

A disease-based bias currently exists, as the vast majority of gene annotations originate from repositories collected for cancers, rare diseases, or diseases mainly relevant to populations of European ancestry [82]. These potential biases are barriers to addressing the bench-to-bedside gap [5, 10, 78] as well as social disparities [83] in NCD research. As well, we are unaware of any epigenetic studies that have used a multidimensional, population-based measure of risk related to smoking to model foetal programming. Thus, our findings may promote the investigation of genomic regions previously neglected by this literature.

We anticipate that a context-based approach may complement traditional research findings by helping to explain why some DNAm sites that have received intense research focus (and garnered statistical “significance”) have provided no clinical traction in improving the management of NCDs [5, 78]. The intent of our study was not to “label” a child with his/her future outcomes. Like other NCD “biomarkers” in current literature (e.g. serotonin transporter gene polymorphisms (*SLC6A4*) and mental health disorders [84]), our work supports the notion that future outcomes are a result of many pathways where a biological factor may play different roles of varying importance depending on context.

Instead of interpreting these findings to suggest any degree of determinism conferred by life exposures, we believe they demonstrate the potential to uncover common molecular pathways underlying diverse vulnerabilities to risk. This capability will help the scientific and medical communities to foster resilience on a population scale. Further, our aim in exploring a context-based approach was to find a means to realistically model the intersection between genetic, environmental, and random chance effects that underlie maternal-foetal health. The uncovered models of gwDNAm patterns demonstrate reproducibility and specificity. As well, they probe novel interactions and functions among networks of DNAm sites that may offer insight into pathways to future health. We believe such efforts better harmonise science and bedside medicine, shifting the field *towards* context-based design and *away* from the use of discrete categories that invite stereotype-based labels.

### Limitations

We used DNAm data from blood cells. The extent of extrapolation of these findings to target tissues such as adipose and brain cells remains unknown, even though there are clear links between immune function in cardiometabolic and neurocognitive diseases [85, 86]. Though we attempted to attenuate subpopulation-based bias, it remains that the populations under study, microarray used, and various annotation resources are biased to represent primarily Western European-descent individuals. To evaluate generalisability, we will need to replicate and compare these results to those obtained from a mixed-descent population (after re-training and testing the methods). We used basic, open-source R language implementation tools in consideration of accessibility to all users and theoretic fit with our a priori hypotheses. More sophisticated methods (e.g. non-negative tensor factorisation or various artificial intelligence-based algorithms) exist that can better deal with the “high noise to low signal” data conditions and manage simultaneous learning and modelling between risk exposure data, DNAm data, and phenotypic outcomes. We hope this work will stimulate statistical methods in epigenetic and other omic studies that can better characterise and benchmark context-based representations of risks and outcomes. We were unable to compute patterns of change on a genome-wide scale despite having repeated measures of DNAm data due to the multiplicative computational cost of such a large data set. This is an area where techniques from fields like functional neuro- and cardiac-physiology [87] and biometry applications on smart devices [88] may be helpful in dealing with immense, repeated measures of data. We speculate such an advancement would improve model generalisability by increasing precision in characterising heterogeneity, enhancing the utility of limited human biological samples. Last, tree-based models can be difficult to interpret compared to main-effect models. As these methods continue to gain popularity in many fields outside of informatics, future models will have improved interpretability [89, 90].

## Conclusions

Our findings are preliminary, but they join a growing body of research arguing for the exploration of *coordinated* patterns of molecular signals—signals that can funnel a wide diversity of risk profiles to a number of shared clinically relevant traits [91–93]. The gwDNAm patterns in this study provide a population-normalised score akin to how clinical tests have normative reference ranges. In addition to providing population-based context, the patterns demonstrate novel molecular context with remarkable colocation among genomic features critical to chromatin function. We believe our work represents a proof-of-concept for a context-based approach in the study of complex entities like the maternal-foetal dyad. If DNAm patterns discovered in blood can link population heterogeneity and molecular interactivity to future traits, this will enhance generalisability while individualising early risk management and avoiding invasive sampling of target tissues.

### Supplementary Information

**Additional file 1. **Methodological details [94–142].** Figure S1. **Bisulphite-converted DNA plate-related components obtained using normFact R function (spatiotemporal independent component analysis, alpha = 0) on cord ARIES DNAm data. **Figure S2.** Correlation matrix of maternal risk profile variables. **Figure S3. **Graphical representation of the decomposition of data into two sparser matrices.

**Additional file 2. **Supplementary results [143–149].** Figure S1. **Scree plot of factor analysis of maternal smoking profiles data.** Figure S2. **Heat map of confounder variables represented by top 20 singular value decomposition (SVD) components. **Figure S3. **Correlation matrix of DNAm components and sex-related singular value decomposition principal components. **Figure S4. **Correlation matrix of DNAm components and singular value decomposition principal components related to social confounders, V13 and V19.** Figure S5. **Correlation matrix of gwDNAm patterns and estimated cell count proportions. **Figure S6. **Blood pressure (z-score by sex), systolic and diastolic.** Figure S7. **Fat mass (z-score by sex) obtained through dual-energy x-ray absorptiometry (DEXA) scanning. **Figure S8. **Lean mass (z-score by sex) obtained through DEXA scanning. **Figure S9. **Weight (z-score by sex). **Figure S10. **Denver Developmental Screening Test – II performance (parental report).** Figure S11. **School performance as assessed on the UK Department of Education scores from standard assessment tests linked to ALSPAC subjects for ages 5-7, 8-11, and 12-14 years. **Figure S12. **Weschler Intelligence Scale for Children-III (WISC) performance at age 8 years. **Table S1.** Random forest metrics – a comparison of three models using cord blood DNAm components and waist circumference as the outcome. **Figure S13. **Replication of gwDNAm patterns at birth– Model testing in peripheral blood at age 7 and age 17. **Table S2. **Performance metrics comparing models in DNAm data (Model 3) at birth and mid- and late childhood in ARIES. **Table S3. **Performance metrics of Model 1 (risk-related variables) in ARIES. **Figure S14. **Component 9 overlaps with DNase I hypersensitivity sites (DHSs) sites more than randomly expected in the genome. **Figure S15. **Component 7 versus control group: meta-EWAS (44). **Figure S16.** Component 19 versus control group: meta-EWAS (44).** Figure S17. **Component 18 versus control group: meta-EWAS (44).** Figure S18.** Locus overlap enrichment analysis (LOEA) using tissue-clustered DHSs (47) (available from LOLA core database) for meta-EWAS and Components 7, 9 and 18.** Figure S19. **Locus overlap enrichment analysis (LOLA) using chromatin marks (available from LOLA Roadmap database) for Component 4. **Figure S20. **All CpG sites overlapping between EWAS candidates from Richmond (48) and each DNAm component.

**Additional file 3. **List of CpGs representative of gwDNAm Components 1-20.

## Data Availability

The data that support the findings of this study are available from the ALSPAC and Generation R executives, but restrictions apply to the availability of these data, which were used under licence for the current study and so are not publicly available. The ALSPAC study website (http://www.bristol.ac.uk/alspac/researchers/our-data/) contains details of all the data that are available through a fully searchable data dictionary and variable search tool. Data from Generation R are available upon reasonable request (generationr@erasmusmc.nl) and are subject to local and national regulations.

## Abbreviations

450K BeadChip: HumanMethylation450 BeadChip
AHRR: Aryl-hydrocarbon receptors repressor gene
ALSPAC: Avon Longitudinal Study of Parents and Children
ARIES: Accessible Resource for Integrated Epigenomics Studies
BMI: Body mass index
NCD: Non-fatal, non-communicable common complex disease
CpG: Cytosine-phosphate-guanine (dinucleotide)
CYP1A1: Cytochrome P450 family 1 member A1 gene
DHS: DNase I hypersensitivity site
DNAm: DNA methylation
EWAS: Epigenome-wide association study
EPIC: Illumina HumanMethylationEPIC BeadChip
FTO: Fat mass and obesity-associated gene
G-CSF: Granulocyte colony-stimulating factor
GFI1: Growth factor independent 1 transcriptional repressor gene
H1: Human embryonic stem cell line
HUES: Human embryonic stem cell line
iPS DF: Induced pluripotent stem line derived from foreskin fibroblasts
LOLA: Locus overlap enrichment analysis
Meta-EWAS: Meta-analysis of the association between sustained maternal smoking and cord blood DNAm, specifically the largest study to date conducted by (44).
MSE: Mean Squared Error
MRP: Maternal risk profiles
PAI: Promoter-anchored chromatin interaction
PCA: Principal component analysis
PMA-I: Phorbol myristate acetate – ionomycin
PLS: Partial least squares
R^2^: Coefficient of determination
RF: Random forest
SD: Standard deviation
