# Supplementary material for: A novel approach to risk exposure and epigenetics—the use of multidimensional context to gain insights into the early origins of cardiometabolic and neurocognitive health
Source: BMC Med. 2023 Nov 27;21:466. doi: 10.1186/s12916-023-03168-z (PMC10683259; doi:10.1186/s12916-023-03168-z)
Supplement: Supplementary file 1 — Additional file 1. Methodological details [94–142]. Figure S1. Bisulphite-converted DNA plate-related components obtained using normFact R function (spatiotemporal independent component analysis, alpha = 0) on cord ARIES DNAm data. Figure S2. Correlation matrix of maternal risk profile variables. Figure S3. Graphical representation of the decomposition of data into two sparser matrices. [file 12916_2023_3168_MOESM1_ESM.docx]

Additional File 1: Methodological Details

**Contents**

[Abbreviations 3](#_Toc148968454)

[A. Exposure variables: Maternal risk profile (MRP)-context 4](#_Toc148968455)

[B. Outcome variables: Child physical and mental development 5](#_Toc148968456)

[C. DNA methylation - ARIES 5](#_Toc148968457)

[i) Laboratory processing, quality control and normalisation 5](#_Toc148968458)

[ii) Batch control 7](#_Toc148968459)

[iii) Cell composition 9](#_Toc148968460)

[Validation cohort – the Generation R Study 9](#_Toc148968461)

[1. Component-based analysis - Describing clinical and molecular context using multidimensional data 10](#_Toc148968462)

[*Step 1-A: Generating population-based maternal risk profiles* 10](#_Toc148968463)

[A. Measures of sampling adequacy for factorisation 11](#_Toc148968464)

[B. Variable contribution 12](#_Toc148968465)

[*Step 1-B: Training models of genome-wide patterns of DNA methylation related to maternal risk profiles* 12](#_Toc148968466)

[*Step 1-C: Testing DNAm patterns on different data sets* 13](#_Toc148968467)

[*Computing resources* 14](#_Toc148968468)

[2. Tree-based analysis - Finding robust relations between variables and outcomes in complex data structures 14](#_Toc148968469)

[*Step 2-A: Select variables using Boruta:* 15](#_Toc148968470)

[*Step 2-B: Run RF model with Boruta-selected variables* 16](#_Toc148968471)

[*Model stability* 16](#_Toc148968472)

[*Computing resources* 17](#_Toc148968473)

[*Missing data and outliers* 17](#_Toc148968474)

[*Evaluating confounder effects* 18](#_Toc148968475)

[Enrichment-based analysis – Finding molecular relevance 18](#_Toc148968476)

# Abbreviations

| ALSPAC | Avon Longitudinal Study of Parents and Children |
| --- | --- |
| ARIES | Accessible Resource for Integrated Epigenomics Studies |
| BCD | Bisulphite-converted DNA |
| CD8T | cytotoxic T lymphocyte cell |
| CD4T | helper T lymphocyte cell |
| 450K BeadChip | Illumina HumanMethylation450 BeadChip |
| cg | Prefix for Illumina Infinium ® CpG loci identification |
| CpG | Cytosine-phosphate-guanine (dinucleotide) |
| CPU | Central processing unit |
| DHS | DNase I hypersensitivity site |
| DNAm | DNA methylation |
| EWAS | Epigenome-wide association study |
| EPIC | Illumina HumanMethylationEPIC BeadChip |
| FAMD | Factor analysis of mixed data |
| Gran | granulocyte cell |
| ICA | Independent component analysis |
| kb | Kilobases (referring to DNA base pairs) |
| MI | Multiple imputation |
| MRP | Maternal risk profile |
| MSE | Mean squared error |
| MSEP | Mean squared error of prediction |
| NK | Natural killer cells |
| PAI | Promoter-anchored chromatin interaction |
| PCA | Principal component analysis |
| PLS | Partial least squares |
| R^2^ | Coefficient of determination |
| RBC | nucleated red blood cell (only applicable to cord blood) |
| RF | Random forest |
| SNP | Single nucleotide polymorphism |
| SVD | Singular value decomposition |
| WISC | Wechsler Intelligence Scale for Children-III ^UK^ |

**Discovery cohort – ALSPAC**

Details of all Avon Longitudinal Study of Parents and Children (ALSPAC) study data are available through a fully searchable data dictionary and variable search tool at http://www.bristol.ac.uk/alspac/researchers/our-data/. An additional recruitment phase occurred when the oldest children were about 7 years old. This led to a total sample size of 15454 pregnancies and 15589 fetuses. Data from this entire cohort was used in this study.

## Exposure variables: Maternal risk profile (MRP)-context

For self-report of maternal smoking, mothers answered the following questions at ~18 and 32 weeks gestation: “Did you smoke regularly at any of the following times in the last 9 months: 1) Before pregnancy, 2) first 3 months of pregnancy and 3) last 2 weeks”. As in previous research in this cohort (48), maternal smoking was collapsed to best reflect the consistency of self-report. A mother was classified as a Sustained smoker if she smoked for at least two trimesters. Non-smokers were those who reported not smoking in all three trimesters. Non-sustained smokers reported smoking in only one trimester.

For familial and household smoking, we used maternal reports at 18 weeks gestation of smoking by partners, other household members, and the histories of the grandfather and grandmother. We included both the grandmaternal history of ever smoking and smoking during the gestation of the subject’s mother. We transformed birth weight into a z-score and adjusted for sex and gestational age using a multi-ethnic, multi-country reference available through the R-package *hbgd* (94).

We also considered the following variables: maternal age, pre-pregnancy body mass index (BMI), pre-pregnancy weight, parity, neighbourhood quality index, paternal educational attainment, maternal social class, maternal alcohol intake and illicit drug use, and paternal smoking. In the end, these variables were excluded from the MRP model based on tuning metrics (see below in Section Component-based analysis - Describing clinical and molecular context using multidimensional data, Part 1: Generating population-based maternal smoking profiles.)

The highest educational qualification was collapsed into non-degree (i.e., one of the five categories from none to Certificate of Secondary Education, the lower level of two national school exams taken when these women were in school at age 16) or having achieved a university degree. Social class is coded according to the British Registrar General’s Scale. Categories with fewer than five observations were collapsed with the most similar category during analysis. However, the descriptive table (Main manuscript, Table 1) includes the actual raw value to demonstrate issues with information loss due to missing data and/or discretisation of categories with low observation counts and analytical challenges posed by large class imbalances (54, 55, 95).

## Outcome variables: Child physical and mental development

Individual growth trajectories calculated using multilevel models with random effects are available for all subjects with two or more measures from the ALSPAC data repository (22). This modelling provides internally derived z-scores for deviations from the average change in weight/height that are calculated by sex (i.e., girls and boys were analysed separately). Anthropometric values with |z-score| > 4 were removed as outliers.

All anthropometric measures were transformed to internal z-scores using all available data in ALSPAC and were calculated by sex. Neurocognitive outcomes were analysed as raw scores.

## DNA methylation - ARIES

Data were collected at three time points. Samples at birth were from cord blood. The samples collected in later childhood originated from peripheral blood (from buffy coats or whole blood). Blood collection occurred around 7 years (mean age 7.5) and between the ages of 15 and 17 years (mean age 17.1 years) in adolescence. To reduce batch effects, there was semi-random sample distribution across chips. Quality control and subject mismatch checks using genotype probes/sex-match were performed as previously described (24). We performed a discovery analysis of relevant DNA methylation (DNAm) patterns from all 914 subjects with cord blood data from ARIES.

### Laboratory processing, quality control and normalisation

All analyses reported in this work employ the Version 2 data release (17-December-2015). This data underwent quality control and preprocessing at the University of Bristol. Data were normalised in R (Version 3.0.1) with the *wateRmelon* package (96) using the algorithm created by (97) to reduce the non-biological differences between probes. The 450K BeadChip probes were filtered by detection *p-*values and variability. Samples with >20% probes with *p*-value ≥ 0.01 were excluded. This left 417832 probes in the cord blood ARIES data from the ∼485000 cytosines tested on the 450K BeadChip. Probes were further filtered for sites possibly confounded by cross-hybridisation with other genomic sites and proximity to single nucleotide polymorphisms (SNPs) (98). We used the *DMRcate* R-package to filter a further ~48000 unreliable probes (99), and 377460 probes remained.

Genotype-dependent methylation describes when a genetic variant may be associated with disease status, and it alters DNAm. In these cases, DNAm levels are associated with disease but may not be causal. Genotype can affect methylation levels in cis and trans locations. When a SNP directly alters methylation, the actual site of methylation can be reduced by half or totally in the heterozygous or homozygous state, respectively (100-102). Methylation can also be altered indirectly by a SNP via a change in a transcription factor binding site, which then changes the local level of DNAm (103) or histone modifications (104). Some studies filter CpGs that demonstrate genotype-dependent methylation in order to isolate the “pure” methylation relation to the variable of interest. However, it may be impossible to completely partition the observed relation to a phenotype that is related to DNAm versus genotype as they are likely intertwined in the regulation of complex trait manifestation.

We elected not to filter CpGs demonstrating genotype-dependent methylation for multiple reasons. First, complex traits likely involve many CpG site sites, and the effect of a single CpG site is small (105). If we assume that CpGs demonstrating genotype-dependent methylation were present, they would have to be concentrated in a DNAm pattern in order to accumulate a detectable effect size and also share the same direction of effect to bias the results for that specific phenotype to be non-null. The chances of this occurring with multiple phenotypes in our study would likely be far lower than obtaining a null result (106).

Second, our study design avoided a gene-centric focus. Non-genic CpG sites are less associated with genotype-associated methylation (107), and they contribute to the landscape of each gwDNAm pattern. Avoiding gene-centric bias may also broaden the generalisability of this methodology to more genetic ancestry groups since most literature regarding genotype-dependent methylation is in individuals of White European ancestry (108). The impact of removing genotype-dependent methylation when applying gwDNAm patterns on populations of different ancestry is unknown and possibly harmful at this discovery stage.

Lastly, the gwDNAm patterns take a global view of the methylation landscape, reducing the likelihood of targeting genotypic admixture rather than stress-related DNAm differences. This is empirically supported in (107), where they found that the top principal components of 450K BeadChip data failed to represent population structure in the way it did on SNP data from the same cohort. They found no associations between methylation components and smoking. We point out this study used principal component analysis (PCA), which performs unsupervised extraction of maximal variability from data. In contrast, we used partial least squares (PLS) analysis, which seeks to optimise shared variation between DNAm and the risk profile and thus would be even less likely to target genotype-dependent methylation on the basis of its large contribution to variability in the data set alone.

DNAm probes with low variability were removed as these data were unlikely to contribute to methodologically distinguishable processes and for managing computation costs. Numerous studies have demonstrated that most CpGs do not contribute to systemic interindividual variation. A recent paper estimated that <1% of the Illumina HumanMethylationEPIC (EPIC) BeadChip (which has nearly double the probes as 450K BeadChip) probes interrogate high variability CpG sites (109). Testing only higher variability probes can also reduce multiple test correction penalties (110). We filtered probes using a threshold of at least 5% change in beta values when taking the difference between the top and bottom methylated values between the 10^th^ and 90^th^ percentile range as performed previously (110-112). This filtered about 200000 probes, leaving 185466 probes for further analysis. We aim to use as much data as possible to distinguish true relations between variables of interest and unmeasured and/or unknown sources of noise (42). These 185466 probes are used as the genomic “universe” that forms the basis of other comparisons (e.g., for enrichment) or replication analyses we perform.

### Batch control

We employed spatiotemporal independent component analysis (ICA) factorisation (R-package *normFact*) to estimate batch variability (113). In the ARIES dataset, the bisulphite-converted DNA (BCD) plate is known to have the strongest batch effect. This ICA method leaves behind a “cleaned” data matrix where, theoretically, variability is due to removing the batch effect. This risks leaving behind variability related to other technical artefacts; however, we spare removing variability intertwined with biological signals (113). Thus, this ICA method complies with our attempt to carefully “unearth” the underlying and subtle MRP-related vulnerability patterns in cord blood.

To implement the *normFact* R function (provided directly by the first author of (113)), the user must set the α parameter (a value bound by 0 to 1) that represents the extremes of two ICA factorisation assumptions: independence among genes versus independence among samples, respectively. An α parameter of 0.5 would thus represent a perfect trade-off between these two extremes. After discussion, the author recommended setting alpha to 0. Figure S1 below shows the *normFact* results on cord DNAm with the relation between the ICA component and each BCD number. We removed eight BCD-related components with R^2^ > 0.5, as suggested by the authors.


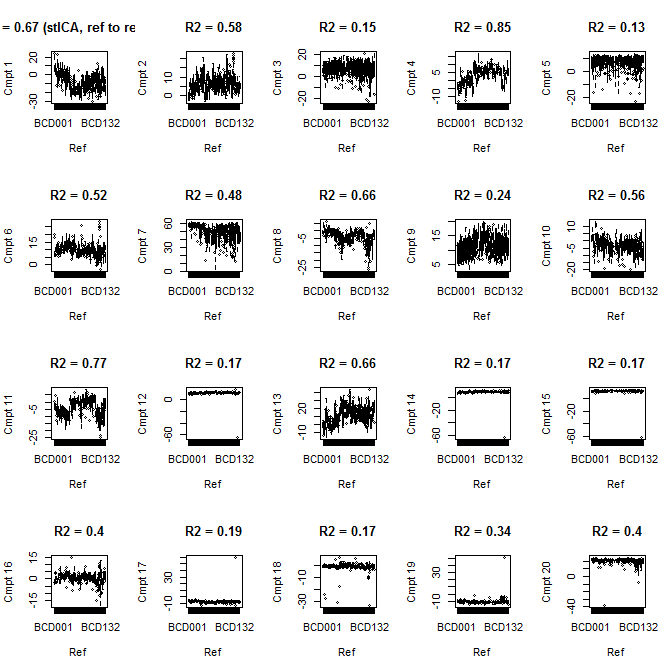


**Figure S1: Bisulphite-converted DNA plate-related components obtained using *normFact* R function (spatiotemporal independent component analysis, alpha = 0) on cord ARIES DNAm data.** Components with R2 > 0.5 are removed.

We used the same probe filtering and batch correction on both cohorts because these could directly impact the computational efficiency, performance, and comparability of downstream component extraction.

### Cell composition

The ARIES database lacks information on cell composition, so we used data-driven methods for cell type correction (104). We estimated cell composition for cord blood DNAm data using the *meffil* R-package (114). The *meffil* R-package has several cord blood cell type references, which were systematically tested on our data set. We selected the gse68456 reference as it has been previously selected for use with ARIES data (115) and provided the least number of negative estimates, perhaps because it excludes erythroid lineage-specific markers. Accordingly, we found very different estimated counts between the two references and between the two nucleated red blood cell (RBC) estimates and their relation with DNAm components (data not shown but available upon request). The cord blood reference uses seven cell types. As in (116), negative proportion estimates are corrected *post hoc* to zero.

For DNAm data from blood collected at later ages, we used the adult cell reference in this R-package and a reference-free method (with the R-package *reFACToR* (117)) for comparison. For the DNAm data for ages 7 and 17, we trialled two adult blood references, blood gse35069 and blood gse35069 complete. The blood gse35069 complete uses eosinophils and neutrophils instead of granulocytes. We found that the blood gse35069 reference generated fewer negative cell composition estimates, so we used it when comparing models between *meffil* and *reFACToR* estimates.

The *reFACToR* R-package is specifically designed to perform reference-free cell type heterogeneity adjustment using PCA. We used this reference-free method as there are no appropriate age-specific blood cell composition references for ages 7 and 15. Using *reFACToR*, we trialled setting k to values 5, 6, and 7 to represent the common cell types sorted by flow cytometry (CD14+ monocytes, CD19+ B cells, cytotoxic T lymphocyte cell (CD4), CD56+ natural killer (NK) cells, cytotoxic T lymphocyte cell (CD8), eosinophils, and neutrophils). We found similar correlations (p < 0.05) between DNAm components and the *reFACToR* components regardless between these three k values (data available upon request.) We set k=5 to represent the major cell types found in healthy individuals outside the newborn period. For both, we conducted cell count estimates after filtering using DMRcate. We also compared this with the reference-based method, *meffil* (using the adult blood gse35069 reference) and found no changes in DNAm component ranking using either method.

# Validation cohort – the Generation R Study

DNA extracted (using the salting-out method) from cord blood from 979 Caucasian children from the Generation R focus cohort was used for this analysis. DNA (500 ng) underwent bisulfite conversion using the EZ-96 DNA Methylation kit (Shallow) (Zymo Research Corporation, Irvine, USA). Samples were plated onto 96-well plates in no specific order. Samples were processed with the Illumina Infinium HumanMethylation450 BeadChip (Illumina Inc., San Diego, USA). Quality control of analysed samples was performed using standardised criteria. Samples were excluded in case of low sample call rate (<99%, six samples excluded), colour balance >3 (no samples excluded), low staining efficiency (no samples excluded), poor extension efficiency (no samples excluded), poor hybridisation performance (no samples excluded), low stripping efficiency after extension (no samples excluded) and poor bisulfite conversion (1 sample removed). In addition, two samples were excluded because of a sex mismatch.

Probes with a SNP in the single base extension site with a frequency of > 1% in the GoNLv4 reference panel (Genome of the Netherlands Consortium, 2014) and probes with non-optimal binding (non-mapping or mapping multiple times to either the normal or the bisulphite-converted genome) were excluded (118). In total, 49564 probes were excluded, and 436013 probes were analysed. We ran DASES normalisation (97) but with an adapted version of the pipeline developed by (96).

Cell type correction was applied using the cord blood-specific Bakulski reference (49) in the R-package *minfi* (119). This method estimates the relative proportions of seven white blood cell subtypes (CD4, CD8, NK cells, B-lymphocytes, monocytes, granulocytes and RBCs). The sample plate was included in the analyses as a batch variable.

We then overlapped these sites with the genomic “universe” from the ARIES cord data, followed by low variance probe filtering as described above (Section C-i). We systematically tested percentage change thresholds from 1-5% and selected 2% to achieve a comparable microarray dimension size between the two cohorts. This left a final set of 173565 probes. We then proceeded with batch effect removal as described above (Section C-ii).

Consistent with previous literature using these two cohorts, we did not harmonise the normalisation DNAm data (44, 120). As well, the successful recovery of similar signals in GenR despite these technical differences would support the robustness of the findings.

# **1.** **Component-based analysis - Describing clinical and molecular context using multidimensional data**

## *Step 1-A: Generating population-based maternal risk profiles*

We conducted factor analysis using the Factor Analysis of Mixed Data (FAMD) function in the R-package *FactoMineR* (121). FAMD provides information on the inter-relations between variables that are entirely data-driven and produces continuous values representing continuous (i.e., birth weight) and categorical (i.e., smoke exposure) variables. As a PCA-based method, it offers the advantage that the extracted profiles are orthogonal to each other, attenuating multicollinearity issues among predictors in downstream analysis.

Values were centred and scaled before analysis. Missing data were imputed based on the similarity between individuals and the relation between known variables using the imputeFAMD function (iterative regularised mode). We performed tuning using the estim_ncpFAMD function to obtain the number of components used to predict missing data (the result being ncp = 2). Some variables had very low cell counts, such as “yes” for self-reported maternal illicit drug use or being defined as having gestational diabetes. Efficiency in reducing bias through multiple imputations (MI) is optimised when suitable auxiliary measures are supplied to the models. To optimise computational efficiency while objectively considering all potential variables, we initially screened model fit with data from ten imputed data sets. Using the package default, variables were kept if the probability of observing a more extreme value was *p* < 0.05. In our application, the best model was one which could most meaningfully describe and summarise the variables in the data set.

We systematically tested model performance with different combinations of MRP variables in profile construction. We compared combinations of per cent variability captured by the top dimensions and the per cent contribution of the variables to each dimension. We arrived at eight variables that formed the best-fit model. We then repeated the MIFAMD function, this time to create 50 imputed data sets (repeat tuning again arrived at ncp = 2.), and then used the FAMD function to conduct the composite analysis.

## **Measures of sampling adequacy for factorisation**

We examined the factorability of the composite variables using standard criteria. First, four out of eight items correlated with at least one other item (see Figure S2 correlation matrix below). Second, the Bartlett’s test of sphericity was significant (χ2 (28) = 98.2, p = 9.98 x 10^-10^). Third, the overall Kaiser-Meyer-Olkin factor adequacy was 0.53. Given these overall metrics, we proceeded with factor analysis with these eight items.

Figure S2: Correlation matrix of maternal risk profile variables. Factor analysis is based on the correlation between variables in order to assemble them into descriptive categories that express a common “unobserved” influence.

## **Variable contribution**

Variables that are considered to importantly contribute to dimension construction are determined by per cent contribution. The per cent contribution of a variable is calculated as the squared cosine*100/total squared cosine (121). Contributions greater than the inverse of the number of variables (12% in this model) are considered important variable features.

## *Step 1-B: Training models of genome-wide patterns of DNA methylation related to maternal risk profiles*

There are a number of PLS packages available in R. Among those with applications in high-dimensional biological data, these include the *pls* package (specifically the Canonical Powered-PLS function) (122), the *mixOmics* package (specifically the PLS and PLSDA functions) (123) and the *sgPLS* package (124). The former employs an asymmetric PLS form, while the latter two use a symmetric form. Given the size of the DNAm data set, a significant consideration was computer resource requirements. The *sgPLS* package was the most favourable and offered tuning and performance functions that had less intense parallel computing resources.

Before starting analysis, DNAm data was centred and scaled, meaning scores are based on differences in DNAm relative to other sites. As such, when we compare scores from different datasets, we see shifts in patterns more so than changes at single sites. This decreases the impact of confounding variation at specific DNAm sites due to technical artefacts and reverses causation (i.e., phenotype rather than exposure influencing DNAm.)

We conducted hyperparameter tuning using the perf and tuning.sPLS.X functions to attenuate overfitting and accuracy over-estimation (as described by the package authors) (124). This included optimisation by M-fold CV and test grids to compare performance using various ranges of the number of DNAm components, the number of MRP dimensions, and the number of DNAm sites to retain. For components, we varied the number from 1 to 50 in increments of 10. We used the following performance metrics: predictive residual sum of squares (PRESS), R^2^, mean squared error (MSE) of prediction (MSEP), and proportion of DNAm variability captured.

For MRP dimensions, we systematically trialled the number from 2 to 10 dimensions. To tune the number of DNAm variables to retain, we used the tuning.sPLS.X function in the *sgPLS* R-package and compared the MSEP values of each model. We used a 10-fold CV and a grid of keepX values (representing the number of CpG sites to retain) of 500, 1000, 2000, 5000 and 10000. For the majority of models, keepX optimised at a value of 1000. Previous work with PLS suggests that 5- to 10-fold CV offers a good balance between performance and computational efficiency (122). We performed training with 75% of the total sample randomly selected using the *dplyr* R-package. This selection was random with the caveat that training was only conducted on subjects with complete maternal smoking data, which consisted of 901 of the 914 cord blood DNAm samples. We found the best fit using 5 MSPs and 20 components (data available upon request). With these parameters, we created the final model using all 914 cord blood samples.

## *Step 1-C: Testing DNAm patterns on different data sets*

We extracted PLS components from other data based on the PLS model from cord DNAm by using the predict function of the *sgPLS* R-package. Similar to any other regression “prediction” technique, this function could be described roughly to calculate the component scores of the new data set based on the regression coefficients of each CpG site (from the loadings matrix of Figure S3 below) projected onto the DNAm data matrix.

As opposed to traditional PLS algorithms, the sparse PLS function selects DNAm sites using *L*_1_ penalisations on the loading vectors of the DNAm matrices, which are conceptually equivalent to the loadings described for PCA. This promotes “sparsity” by finding the optimal model using as few variables as possible. Besides reducing overfitting, this function selects DNAm sites most representative of variability underlying a specific pattern of gwDNAm. This allows empirical selection of clinically relevant DNAm sites consistent with *a priori* objectives and avoids *post hoc* analysis with hundreds of thousands of multiple hypothesis tests.

Full mathematical details are found in (124, 125).


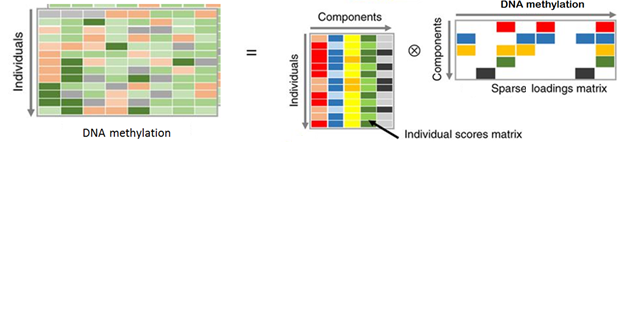


Figure S3: Graphical representation of the decomposition of data into two sparser matrices. Image adapted from (126) with author’s permission. We aim to use partial least squares to represent maternal risk profile-related variability within DNAm data using a small number of components.

## *Computing resources*

All analyses were conducted in R programming language (27). Most analyses were run on a standard central processing unit (CPU). The exceptions were the imputation of 50 sets using MIFAMD and parameter tuning with the tuning.sPLS function. The latter took the longest (approximately 4 days to run on a Linux server with 16 cores and 320GB of RAM with no parallelisation.)

# **2.** **Tree-based analysis - Finding robust relations between variables and outcomes in complex data structures**

In part two of the study, we aim to model the reality of complex interactions between multiple biological inputs that lead to a given phenotype. We used decision tree-based analysis, which is described in the manuscript. Random forests (RF) are a type of decision tree analysis that generates thousands of trees through an algorithm that *randomly* shuffles data values. RF can also be used for ranking exploratory variables in high-dimensional data by their relative importance in predicting an outcome (29). It features recursive partitioning and local model fitting to avoid potentially extreme values within independent decision trees. This makes RF relatively robust to outliers, a significant concern in heavily data-driven experiments (127). The Boruta algorithm (31) is an additional function complementing RF that provides a safeguard against false relations between variables and outcomes due to stochastic processes or chance occurrences that are common but difficult to estimate in complex traits in observational human cohorts. Among RF approaches for high-dimensional data sets, it performs highly in terms of sensitivity, sensitivity and stability (128). Its most unique characteristic is the use of “shadow variables” to adjudicate whether a variable is related to the outcome by more than chance.

A shadow variable is a new variable based on existing variable values but is randomly shuffled such that it should bear no correlation to the outcome. In other words, a shadow variable can only be related to the outcome due to data stochasticity. Therefore, shadow variables introduce additional randomness to the model, making it more robust to random chance or correlations that lead to false positives. It generates shadow variables with each RF decision tree and then collates the importance of this variable. Last, it compares the importance of this variable with the other “real” variables using a binomial test to statistically evaluate the likelihood of whether a given variable is more useful than the shadow variable (typically using *p* < 0.05.) With multiple shuffled copies of the shadow variable, Boruta can construct a distribution for comparing the number of times the shadow variable performed better or worse than the other “real” variables. In this way, one can generate a robust estimate of the variable importance using alternative and data-independent criteria for selecting important variables besides prediction (129).

Instead of using prediction metrics, Boruta selects relevant variables by testing if their relation to the outcome exceeds that which could occur by chance. In this situation, we avoid discarding variables that show statistical redundancy or correlation, as that would lead to an incomplete understanding. We compare this to biomarker discovery, which uses prediction metrics to seek the fewest variables that can generate the most efficient, high-performing model. As referred to in the main manuscript, we applied a two-step RF strategy to discover all relevant variables in omic data as described in (113), which we outline below.

## *Step 2-A: Select variables using Boruta:*

We used the Boruta algorithm (using the R-package of the same name) to obtain all relevant predictors, including the covariates of sex, socioeconomic indicators, and blood cell type estimates. We increased the *ntree* and *maxRuns* values to 1000 since a previous study suggested that high-dimensional datasets may encounter unstable importance scores and false negatives when *ntree* is inadequate (129). Component recurrence may support a more robust association with the outcome, as each model places all variables, including sex, social factors and relevant covariates in “competition” for relevance. Thus, components are considered for further examination if they are consistently selected across outcomes after thousands of iterations and re-samplings and are not related to covariates. We retain only variables that Boruta determines are “Confirmed attributes,” meaning they clearly exceed the importance of the maximum shadow variables after the number of model runs.

## *Step 2-B: Run RF model with Boruta-selected variables*

Using Boruta-selected variables, we ran the RF model in the *caret* R-package (35). We used the train function (*ntree* = 1000) and five-fold cross-validation with three repeats as the resampling method.

RFs are known to perform well with little parameter tuning. We tuned the number of variables randomly selected at each split (called *mtry* in *caret*) using a grid. Consistent with previous biological applications of RF, we also found a minimal effect of *mtry* over a wide range of values surrounding the square root of the number of predictor variables (e.g. SNPs, methylation probes, etc.) (31). We also systemically compared models using different training:test data partitions (i.e., 70:30, 50:50 and 30:70) with various *mtry* and *ntree* combinations. Most literature focuses on prediction using *ntree* between 100–1000 (128). Consistent with previous evaluations of RF on both real-life and simulated data, *ntree* was set to 1000 both for feature selection with Boruta and model estimation with randomForest (31, 130). This number of trees is about 10 times higher than the defaults. We aimed for these settings to be sensitive enough to detect true predictors with small effects and interactions yet remain computationally feasible (131). We compared performance using R^2^ and root MSE (RMSE). The default parameters were good, if not the best, options, as seen in other studies (30, 132, 133). However, the differences among changed settings were marginal (data available upon request), so we used default settings for the sake of reproducibility in all our analyses.

We used RMSE and coefficient of determination (R^2^) metrics to compare models with and without Boruta pre-selection of variables (133) using *caret*.

## *Model stability*

We further checked the stability of the results by rerunning the analyses twice with different random seeds, as described in (134). We also compared the relative ranking of relevant predictors by Boruta and by RF after Boruta selection.

To assess whether our findings were due to specific and unknown properties of our data and the Boruta algorithm, we compared models using RF without Boruta. We used variance explained and error rate on out-of-bag samples as performance metrics.

Because RF algorithms do not accept missing values, we conducted sensitivity analysis by comparing models, with and without imputation, to assess the impact of change in sample size and missing data points. We observed no change in the overall rank of importance of features and only minor shifts in importance values. We also checked the relative ranking of importance of the variables exceeding the maximum importance of the shadow attributes and the performance metrics using another widely used RF algorithm (R-package *rfrsc* (135)). No change in relative ranking was seen. Data is available upon request.

## *Computing resources*

The two-step RF took approximately 20 minutes for each model to run on a CPU with 320GB of RAM.

## *Missing data and outliers*

A small sample size (which may be exacerbated by the aggressive removal of missing data or outliers) can increase model vulnerability to outlier effects. Deletion of outlying data points aggravates missingness. We strove to balance these considerations in our management based on the question being asked and the data characteristics.

With missing data in forming the MRPs, we decided to use imputation when using the entire ALSPAC dataset, considering that the vastness of complete data would make this correction less corruptive of true data relations (136). Once the smaller DNAm subset was used, incomplete cases were removed.

We evaluated each analysis situation individually to weigh the pros and cons of different methods to decrease the impact of outliers. Like all PCA-based methods, the FAMD technique used to create MRPs is sensitive to outliers. As such, it appeared prudent to remove these cases. We used the ind_contrib_cid function in the *FactoMineR*  R-package to observe outliers, specifically subjects with a cos^2^ equal to or greater than 0.8, which is 3 standard deviations (SD) above the mean. There were only 3 outliers in the approximately 8500 ALSPAC subjects with complete data among the eight MRP variables. We used histograms of the distribution of outcomes to compare these outliers to the rest of the ALSPAC subjects. The distributions were typical. For example, only one child had a weight > 3 SD.

These individuals had typical outcome distributions and thus would more likely lead to a null rather than an inflated relation between MRPs and outcomes. Considering that these subjects may represent true population heterogeneity and were unlikely to bias downstream analysis toward extremes, we did not remove these subjects. RF is considered more robust to outliers. In these analyses, we excluded individuals with outcome values with absolute z-scores greater than 4.

## *Evaluating confounder effects*

Among many social factors available (see above in Section A. Exposure variables: MRP-context), we selected maternal education and paternal social status given their previous associations with offspring cardiometabolic and neurocognitive outcomes. Also, the DNAm in this data set (38, 39, 137-140) had comparatively low missing values and demonstrated the least multicollinearity among socioeconomic indicators.

For DNAm data, we used singular value decomposition (SVD) to better represent sex, maternal education and paternal social status as principal components rather than categorical variables using R-package *Champ* (36). The significance of the relation of these SVD principal components (PCs) to each confounder was inferred by comparing them against the null distribution obtained by considering random matrices. Acknowledging the statistical and biological impracticality of removing variability due to these “confounders” when studying fetal risk exposure, we opted not to use the SVD PCs as control variables but instead use them to monitor which associations between DNAm components and outcome were suspicious for confounding.

In all RF models we included control variables known to influence the outcome. For example, previous research has already demonstrated that DNAm differences are related to body composition, both before and concurrent with DNAm sampling (see (123, 141) for recent examples). As well, weight velocity in infancy is known to influence peri-pubertal fat mass, waist circumference, and weight. We wish to avoid focusing on associations between the DNAm patterns and future outcomes that may be due to the tendency of certain phenotypes to demonstrate continuity as we age. Thus, the anthropometric models tend to have several more covariates than other outcome models. This is performed to hopefully distinguish DNAm differences that are due to maternal smoking-related vulnerability.

# **Enrichment-based analysis – Finding molecular relevance**

gwDNAm patterns overlapped with previous candidates, so we conducted analyses to evaluate overlap with features related to chromatin state, accessibility, transcription factor binding, and cell-specific markers.gwDNAm pattern-representative sites were made into genomic range objects using the *minfi, GenomicRanges and IlluminaHumanMethylation450kanno.ilmn12.hg19* R-packages.

To assess if the overlap between the gwDNAm pattern-representative sites with a molecular feature was beyond what could be expected by chance, we performed an over-representation analysis that compared the overlap with a background list (which differed depending on the content of the molecular feature.) We used three publicly available tools depending on the chromatin feature being analysed.

We used the *regioneR* R-package (142) to estimate enrichment for genomic sites without a pre-defined region set database (i.e., chromatin PAI and loop regions). The overlap distribution in 100 to 10000 random circular permutations was used to compute a *p*-value by comparing the observed value with the null distribution (using a background of the human autosomal genome as provided in BSgenome.Hsapiens.UCSC.hg19.masked: Full masked genome sequences for Homo sapiens - UCSC Version hg19. R-package Version 1.3.99). Fewer permutations lead to increased chances of Type II errors (i.e., false negative); however, greater permutations can vastly increase computational time.

We trialled the number of permutations in a grid of 100 to 10000 in intervals of 10X and examined if testing could be completed within 2 hours without parallelisation. For example, DNase I hypersensitivity sites (DHSs) map to numerous locations, resulting in higher chances of overlap and thus less risk of Type II error. We selected 100 permutations since the execution time was about 7000 seconds, and there were no further tests with *p*-values exceeding 0.05 at higher permutation numbers. In contrast, there are relatively few chromatin features such as PAI, and 10000 permutations were conducted within the same time frame. Of note, no testing could be conducted if < 1 overlap occurred.

We used the *LOLA* R-package (46) to assess overlap based on hypergeometric distribution. We used pre-existing database region sets (i.e., tissue-specific transcription factor and DHS sites.) The following databases were accessed on 8 June 2022:

LOLA Region Databases (Core, Tissue clustered DHSs). <http://big.databio.org/regiondb/LOLACoreCaches_180412.tgz>

LOLA Region Databases (Extended, Roadmap epigenomics regions). <http://big.databio.org/regiondb/LOLARoadmap_180423.tgz>

We used the *XSTREME* tool (45) available at meme-suite.org to find the relative enrichment of transcription factor binding motifs of gwDNAm patterns compared to candidates identified through meta- epigenome-wide association study (EWAS) (44). Enrichment analysis used the Simple Enrichment Analysis tool (employing Fisher’s exact test). The primary sequence entered was from a given gwDNAm pattern. The background sequence was the meta-EWAS candidates. Both sequences were extended 250bp in both directions from the probe site. We used default program settings.

We used the “blocks of probes” annotation proposed in (77) to identify the distribution of gwDNAm patterns in the genome relative to other CpGs (e.g., islands) and genes. Data is available upon request.
