# Supplementary material for: A novel approach to risk exposure and epigenetics—the use of multidimensional context to gain insights into the early origins of cardiometabolic and neurocognitive health
Source: BMC Med. 2023 Nov 27;21:466. doi: 10.1186/s12916-023-03168-z (PMC10683259; doi:10.1186/s12916-023-03168-z)
Supplement: Supplementary file 2 — Additional file 2. Supplementary results [143–149]. Figure S1. Scree plot of factor analysis of maternal smoking profiles data. Figure S2. Heat map of confounder variables represented by top 20 singular value decomposition (SVD) components. Figure S3. Correlation matrix of DNAm components and sex-related singular value decomposition principal components. Figure S4. Correlation matrix of DNAm components and singular value decomposition principal components related to social confounders, V13 and V19. Figure S5. Correlation matrix of gwDNAm patterns and estimated cell count proportions. Figure S6. Blood pressure (z-score by sex), systolic and diastolic. Figure S7. Fat mass (z-score by sex) obtained through dual-energy x-ray absorptiometry (DEXA) scanning. Figure S8. Lean mass (z-score by sex) obtained through DEXA scanning. Figure S9. Weight (z-score by sex). Figure S10. Denver Developmental Screening Test – II performance (parental report). Figure S11. School performance as assessed on the UK Department of Education scores from standard assessment tests linked to ALSPAC subjects for ages 5-7, 8-11, and 12-14 years. Figure S12. Weschler Intelligence Scale for Children-III (WISC) performance at age 8 years. Table S1. Random forest metrics – a comparison of three models using cord blood DNAm components and waist circumference as the outcome. Figure S13. Replication of gwDNAm patterns at birth– Model testing in peripheral blood at age 7 and age 17. Table S2. Performance metrics comparing models in DNAm data (Model 3) at birth and mid- and late childhood in ARIES. Table S3. Performance metrics of Model 1 (risk-related variables) in ARIES. Figure S14. Component 9 overlaps with DNase I hypersensitivity sites (DHSs) sites more than randomly expected in the genome. Figure S15. Component 7 versus control group: meta-EWAS (44). Figure S16. Component 19 versus control group: meta-EWAS (44). Figure S17. Component 18 versus control group: meta-EWAS (44). Figure S18. Locus overlap e [file 12916_2023_3168_MOESM2_ESM.docx]

Additional File 2: Supplementary Results

Contents

[Abbreviations 3](#_Toc149048813)

[DNAm profiles in the context of population-based maternal risk profiles 4](#_Toc149048814)

[**Figure S1** 4](#_Toc149048815)

[Relation of DNA patterns to confounders and maternal risk profiles 5](#_Toc149048816)

[**Figure S2** 5](#_Toc149048817)

[**Figure S3** 7](#_Toc149048818)

[**Figure S4** 8](#_Toc149048819)

[**Figure S5** 9](#_Toc149048820)

[gwDNAm patterns at birth relate to future outcomes 9](#_Toc149048821)

[**Figure S6** 11](#_Toc149048822)

[**Figure S7** 12](#_Toc149048823)

[**Figure S8** 13](#_Toc149048824)

[**Figure S9** 14](#_Toc149048825)

[**Figure S11** 16](#_Toc149048826)

[**Figure S12** 17](#_Toc149048827)

[Sensitivity analysis 17](#_Toc149048828)

[Table S1 18](#_Toc149048829)

[gwDNAm patterns generalise over time and across populations 19](#_Toc149048830)

[1) Replication in the ARIES cohort – peripheral blood samples in later childhood 19](#_Toc149048831)

[**Figure S13** 20](#_Toc149048832)

[**Table S2** 23](#_Toc149048833)

[**Table S3** 27](#_Toc149048834)

[2) External validation in GenR cohort – cord blood samples 28](#_Toc149048835)

[gwDNAm patterns have distinct structural and functional molecular features 28](#_Toc149048836)

[1) Chromatin regulation 29](#_Toc149048837)

[**Figure S14** 29](#_Toc149048838)

[2) Motif discovery 29](#_Toc149048839)

[**Figure S15** 32](#_Toc149048840)

[**Figure S16** 33](#_Toc149048841)

[**Figure S17** 34](#_Toc149048842)

[3) Tissue specificity 35](#_Toc149048843)

[**Figure S18** 36](#_Toc149048844)

[**Figure S19** 37](#_Toc149048845)

[Overlap with previous literature 39](#_Toc149048846)

[**Figure S20** 39](#_Toc149048847)

# Abbreviations

| AHRR | Aryl-hydrocarbon receptors repressor gene | |
| --- | --- | --- |
| ALSPAC | Avon Longitudinal Study of Parents and Children | |
| ANOVA | Analysis of variance | |
| ARIES | Accessible Resource for Integrated Epigenomics Studies | |
| BCD | Bisulphite-converted DNA | |
| BMI | Body mass index | |
| cg | Prefix for Illumina Infinium ® CpG loci identification | |
| CpG | Cytosine-phosphate-guanine (dinucleotide) | |
| CYP1A1 | Cytochrome P450 family 1 member A1 | |
| DEXA | Dual-energy x-ray absorptiometry | |
| DHS | DNase I hypersensitivity site | |
| DNAm | DNA methylation | |
| DOHaD | Developmental origins of health and disease hypothesis | |
| EWAS | Epigenome-wide association study | |
| FTO | Fat mass and obesity-associated gene | |
| GFI1 | Growth factor-independent 1 transcriptional repressor gene | |
| kb | Kilobases (referring to DNA base pairs) | |
| LOLA | Locus overlap enrichment analysis | |
| MANOVA | Multivariate analysis of variance | |
| ML | Machine learning | |
| MRP | Maternal risk profile | |
| MSE | Mean squared error | |
| NCD | Non-communicable diseases | |
| p>>n | Number of variables greatly exceeds number of observations/subjects | |
| PAI | Promoter-anchored chromatin interaction | |
| PC | Principal component | |
| PCA | Principal component analysis | |
| PLS | Partial least squares | |
| R^2^ | Coefficient of determination | |
| RF | Random forest | |
| SNP | Single nucleotide polymorphism | |
| SVD | Singular value decomposition |  |
| WISC | Wechsler Intelligence Scale for Children-III ^UK^ | |

# DNAm profiles in the context of population-based maternal risk profiles

The scree plot below shows the scree plots from factor analysis of the eight risk variables. These eight variables are referred to as Maternal Risk Profile (MRP)-related variables and are summarised in Table 2 in the main text.


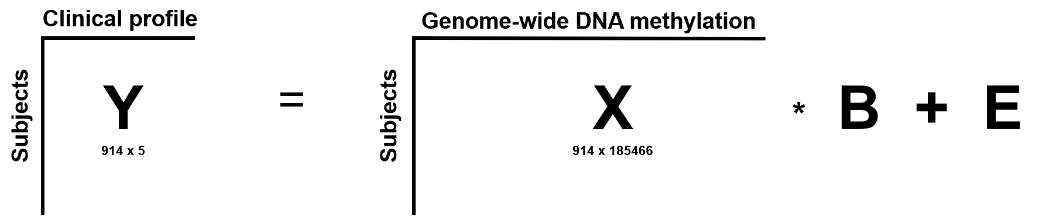

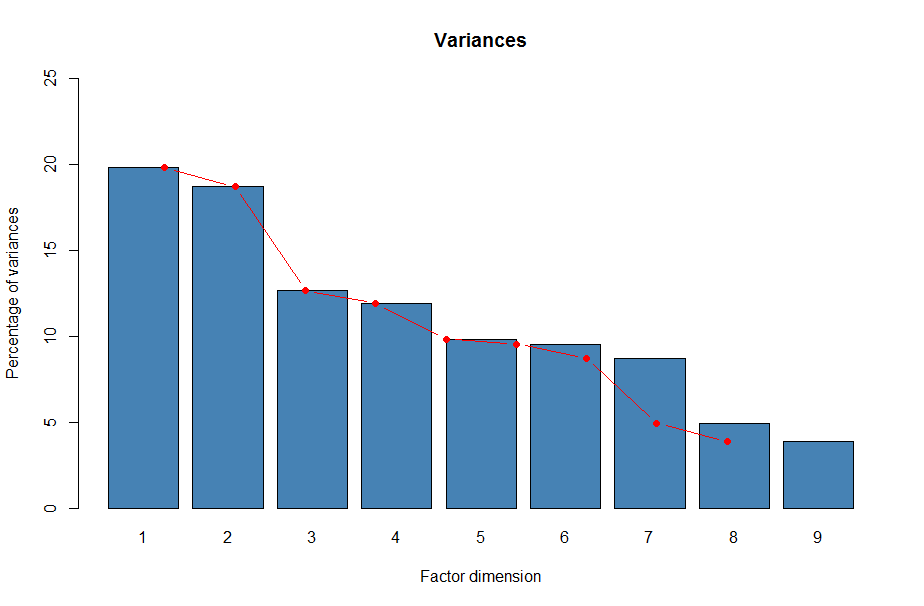


Figure S1: Scree plot of factor analysis of maternal smoking profiles data. Component-based analysis can capture heterogeneous risk profiles related to maternal smoking. Plot of variance captured from factor analysis using eight variables related to MRP (n = 15102.) Inset: Schematic of the partial least squares (PLS) regression model. PLS extracts components from DNA methylation data that predict MRP (n = 914). Y represents the MRPs, X represents DNAm data, B represents the coefficient matrix, and E represents the error.

In the main manuscript, Figure 2 displays what variables each dimension predominantly represents. We can gauge the importance of a variable to a dimension using its relative contribution to the dimension’s construction. Coloured bars exceed the threshold. The threshold is considered contributions greater than the inverse of the number of variables which is 12.5% in this model. A contribution is = squared cosine*100/total squared cosine. (121)

# Relation of DNA patterns to confounders and maternal risk profiles


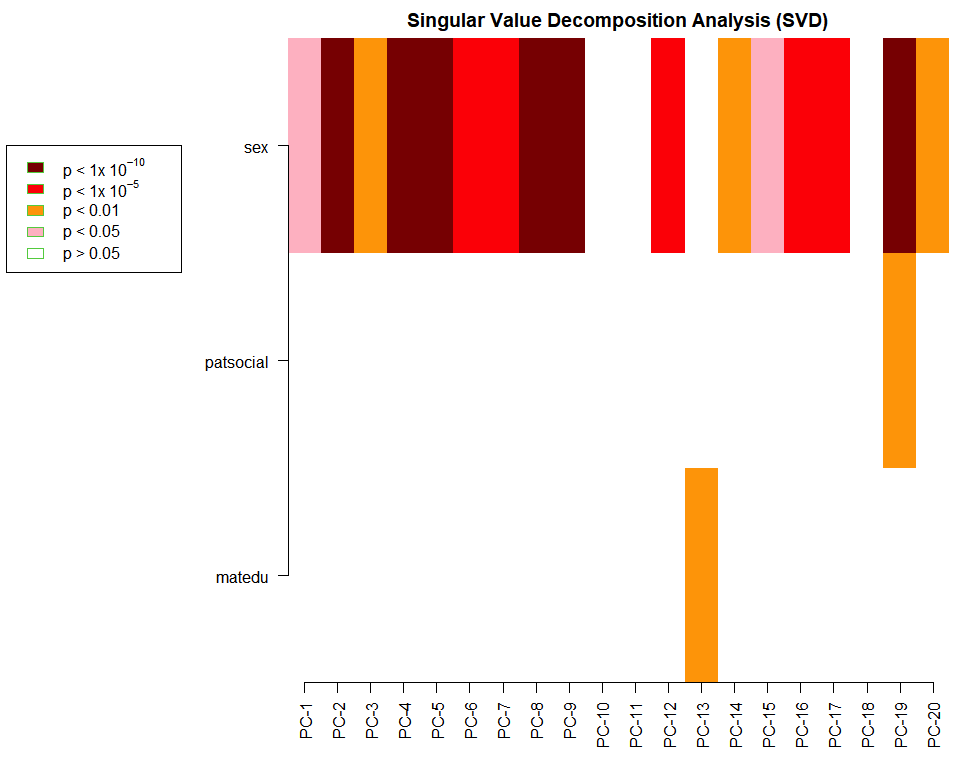
Singular value decomposition (SVD) identified 20 principal components (PCs) related to sex, paternal social status, and maternal education (Figure S2). We observed that nearly all these SVD PCs (not DNAm components), excluding 10, 11, 13, and 18, were related to sex. Paternal social status and maternal education were associated with SVD PC 13 and 19, respectively.

**Figure S2: Heat map of confounder variables represented by top 20 singular value decomposition (SVD) components (x-axis).** We performed SVD on DNAm data to visualise the most significant sources of variation in DNAm data related to confounders. Coloured bars indicate SVD principal components (PCs) that represent these confounders well. All but 4 were related to subject sex. Only SVD PCs 13 and 19 were related to paternal social status and maternal education, respectively. Legend: Colours represent the *p*-value of the relation as evaluated using random matrices. SVD was performed on the 185466 CpGs constituting the filtered data set as described in Additional File 1.

We used SVD as described in (36) on DNAm data to represent sex, maternal education, and paternal social status as continuous DNAm-based variables rather than categorical variables. (Note that SVD PCs represent confounder variables and are different from partial least squares (PLS) components that were generated to represent DNAm data. To avoid confusion between these two components in the subsequent figures, SVD PCs will have the prefix “V”.) This helps us draw more robust conclusions regarding the sources of variability that underlie each DNAm component.

In Figure S3, we observed that the strongest correlations between sex-related SVD PCs with DNAm Components 1-3, 5, 6, and 10. As well, there is overlap of DNAm Components 1, 3 and 6 with cell type proportion-related SVD PCs (Figure S5.) Sex- and cell type-based variability is embedded in human biology and likely impossible to “erase” from associations. Therefore, we will be cautious in interpreting phenotypic relations with these components as they could be due in part to these confounders. We had viewed DNAm components 4, 15 and 19 as well as 13 and 18 with guarded interest given the weaker association with sex or cell type, respectively, as they could still contain potentially informative data. However, we later observed that Component 4 also demonstrated sex-specificity but no cell type specificity on analysis of molecular features, (Figure S19). Moreover, this component was strongly correlated to the maternal smoking-related MRP, Dimension 2. This may indicate multiple sources of variability converge at this component, including bystander effects from direct smoking exposure that may be unrelated to the aetiology of phenotype. Given we are unable to dissect apart such variability to extract mainly that from the biology of interest, we included Component 4 on our list of DNAm components with strong evidence of confounding. Unlike sex-related variability, the correlations between SVD representations of paternal social status or maternal education and DNA components were all weak (i.e., *r* < |0.4|, Figure S4.)


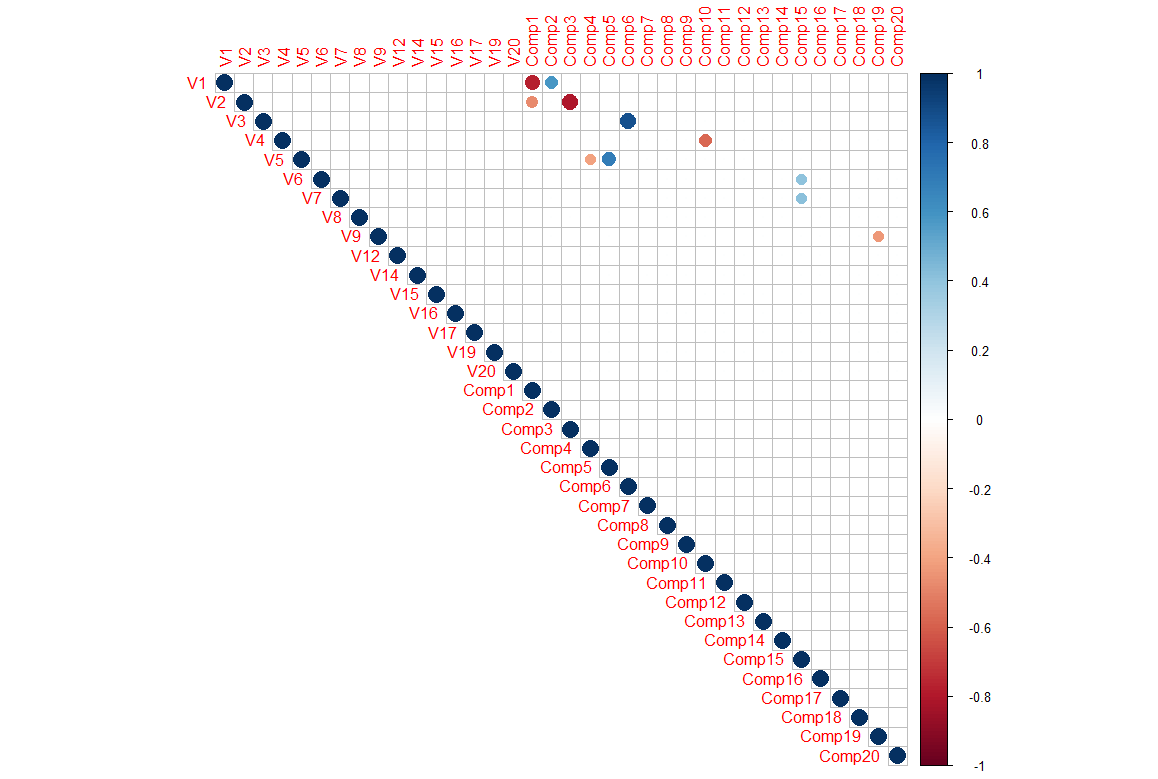


Figure S3: Correlation matrix of DNAm components (prefix “Comp”) and sex-related singular value decomposition (SVD) principal components (PCs) (prefix “V”-as seen in Figure S2.) For the sake of clarity, only non-weak correlations of *r* ≥ |0.4| and *p* < 0.05 are shown. The circle size is inversely proportional to the *p*-value. Components with a circle are correlated with sex. Legend: Colour refers to Pearson correlation value.

Figure S4: Correlation matrix of DNAm components (prefix “Comp”) and SVD PCs related to social confounders V13 and V19. As seen in Figure S2, these two SVD PCs are related to paternal social status and maternal education, respectively. For clarity, only correlations with *p*-values < 0.05 are shown. The size of the squares is inversely proportional to the *p*-value. Components 12 and 17 are weakly correlated to paternal social status (i.e., SVD PC V13, *r* ~ |0.3|). Legend: Colour refers to Pearson correlation value.


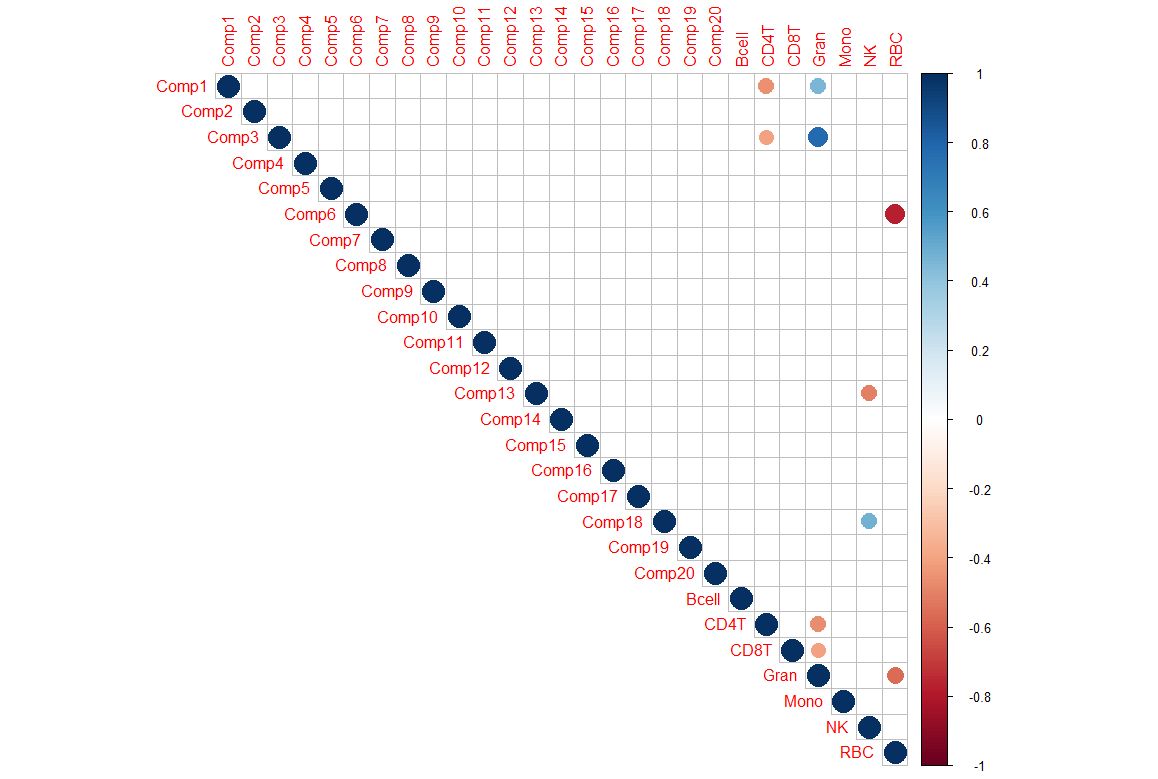


**Figure S5:** **Correlation matrix of gwDNAm patterns (prefix “Comp”) and estimated cell count proportions**. For the sake of clarity, only non-weak correlations of *r* ≥ |0.4| and *p* < 0.05 are shown. The circle size is inversely proportional to the *p*-value. Looking across the rows, Components 1, 3, and 6 have strong or more than one correlation with cell type proportion. Components 13 and 18 have a moderate correlation to natural killer cell proportions. Legend: Colour refers to Pearson correlation value.

# gwDNAm patterns at birth relate to future outcomes

The following figures show relevant predictors of outcomes as described in Table 1 of the main manuscript. The horizontal panels indicate the source of DNAm data (on the right axis from bottom to top: cord blood, peripheral blood at age 7, and at age 17.) Each row represents an outcome (on the left axis, the suffix indicates the age at the time of outcome measurement.) Colours distinguish each DNAm component. This section only uses data from the Avon Longitudinal Study of Parents and Children (ALSPAC) cohort**.**

The z-score of the estimated growth rate has the following abbreviations:

| **Age period** | **Average change in weight** | **Average change in height** |
| --- | --- | --- |
| Birth to 3 months | zwres1 | zhres1 |
| 3 to 12 months | zwres2 | zhres2 |
| 12 to 36 months | zwres3 | zhres3 |

Figure S6: Blood pressure (z-score by sex), systolic and diastolic. Given the dependence of blood pressure on height, we used early growth rate in height rather than weight. Like other cardiometabolic outcomes, this early growth is the most consistent variable to be selected by Boruta.We observe that DNAm Components 9 and 18 were selected at birth. Testing of DNAm data from blood in mid- (age 7 years) and late-(age 17 years) childhood replicated this relation. We continue to see in blood collected at mid-childhood that DNAm components still relate to future blood pressure (i.e., at age 11 years.) This argues against reverse causality being the main driver of this observed relation i.e., blood pressure exerts its influence on DNAm. Components 7 and 11 were only selected in blood in mid- and late-childhood. Recall that there was higher sample size of DNAm data at later ages, which may be responsible for the “appearance” of components only later in life. Recall that Components 7, 9 and 18 were also selected to predict future waist circumference. Z-score of the average estimated change in height over infancy indicated by zhres1 (ages 0-3 months), zhres2 (ages 3-12 months), zhres3 (ages 12-36 months). Components 1-6 and 10 are not discussed due to their strong relations to confounders.

Figure S7: Fat mass (z-score by sex) obtained through dual-energy x-ray absorptiometry (DEXA) scanning. DEXA estimates total fat mass, which includes visceral and subcutaneous fat. Here, DNAm Component 18 is related to future fat mass in cord blood. Component 18 was also selected to predict future waist circumference, which is highly correlated with visceral fat. There was replication in blood drawn in late childhood, but this was after the fat mass measurement, so no inferences arguing against reverse causality can be made here. Z-score of the average estimated change in weight over infancy indicated by zwres1 (ages 0-3 months), zwres2 (ages 3-12 months), zwres3 (ages 12-36 months). The results of Components 1-6 are not discussed due to their strong relations to confounders.

Figure S8: Lean mass (z-score by sex) obtained through DEXA scanning. Even though fat and lean mass were both measured using DEXA, DNAm Component 19 (not 18) was selected in cord blood. Components 7 and 9 were again selected. Component 11 only was selected as relevant in DNAm data in mid- and late childhood as seen in models of blood pressure. Z-score of the average estimated change in weight over infancy indicated by zwres1 (ages 0-3 months), zwres2 (ages 3-12 months), zwres3 (ages 12-36 months). The results of Components 1-6 and 10 are not discussed due to their strong relations to confounders.

Figure S9: Weight (z-score by sex). Components 7 and 18 were both selected in cord blood and replicated in DNAm data drawn in late childhood. Z-score of the average estimated change in weight over infancy indicated by zwres1 (ages 0-3 months), zwres2 (ages 3-12 months), zwres3 (ages 12-36 months). The similar outcomes observed in Components 2, 3, and 6 are not discussed due to their strong relations to confounders.


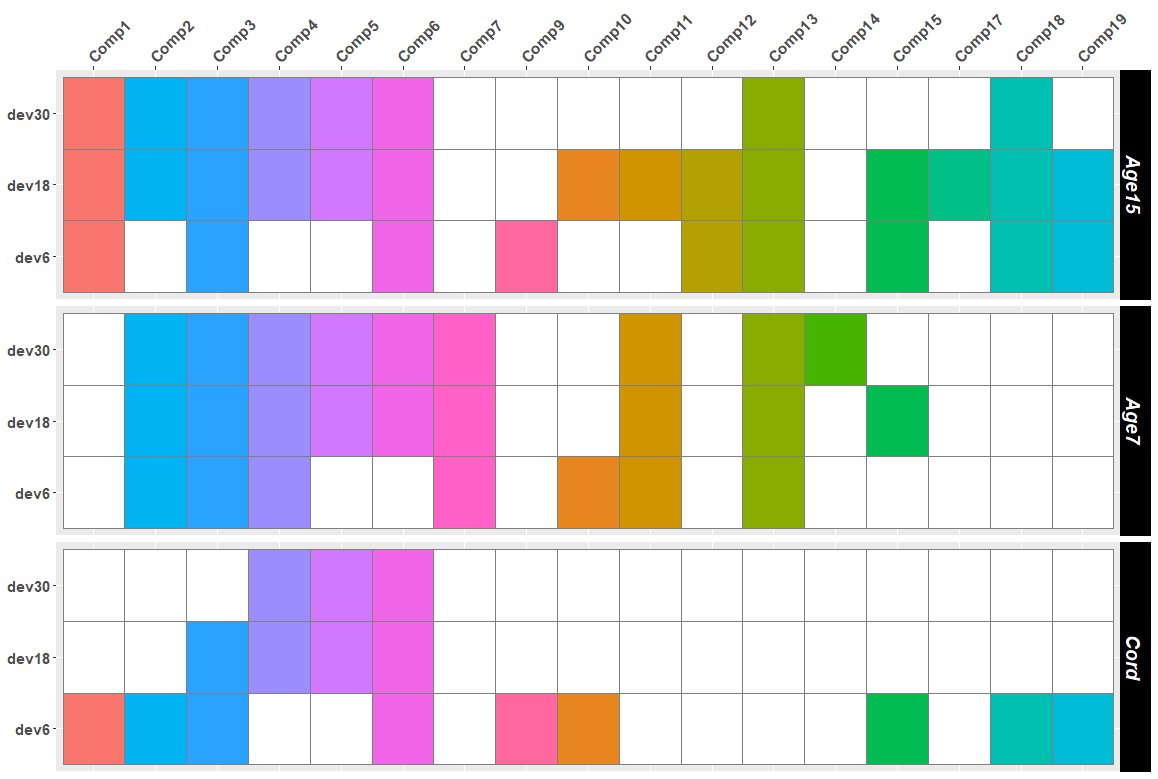
**Figure S10: Denver Developmental Screening Test – II performance (parental report).** Assessments were performed at ages 6, 18, and 30 months of life (left axis - dev6, dev18, dev30, respectively.) Skill domain scores were summed to provide a total score (143). Components 9, 18 and 19 were selected in cord blood. The Denver test was conducted in early childhood, so replication in DNAm drawn in late childhood could be due to the persistence of this DNAm components from birth or due to reverse causality (e.g., the child’s development in early life set the stage for neurocognitive features that tend to generate the same gwDNAm patterns.) We note that Components 9 and 19 are enriched for molecular markers that are specific to neuroectodermal cell lineage (Figure S18 and in main manuscript, Figure 8.)


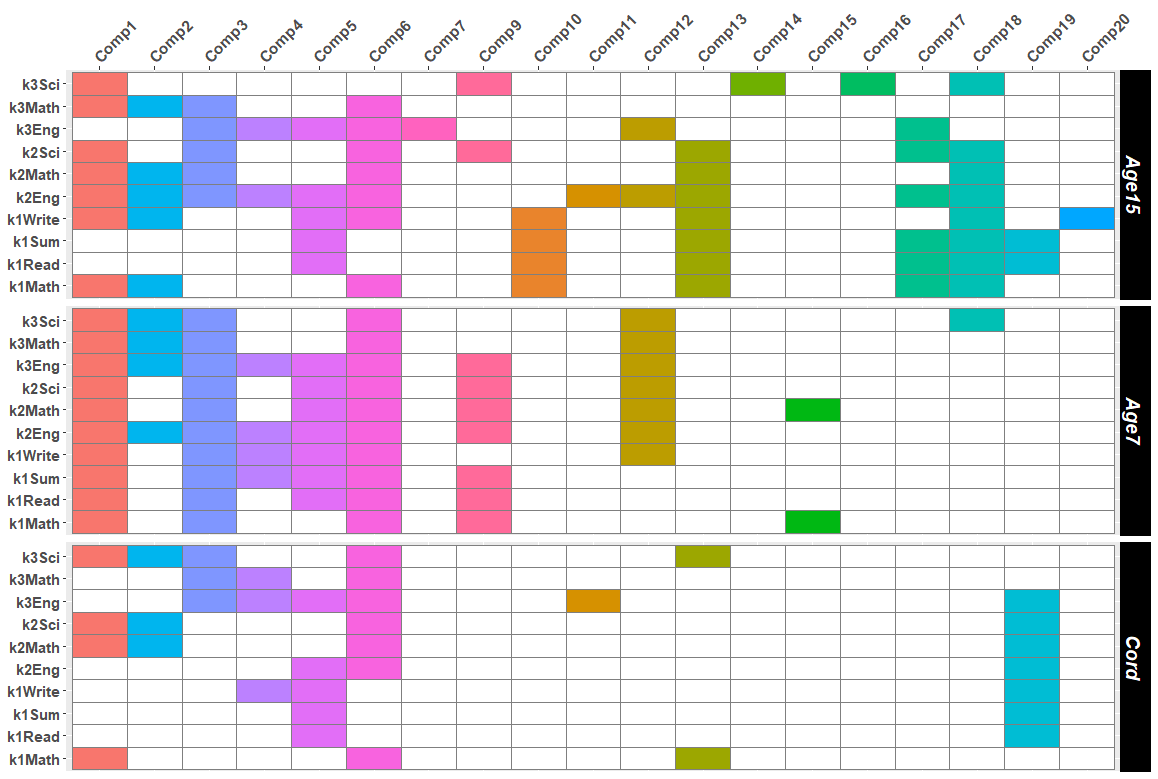


**Figure S11: School performance as assessed on the UK Department of Education scores from standard assessment tests linked to ALSPAC subjects for ages 5-7, 8-11, and 12-14 years** (labelled as K1, K2 and K3, respectively.) Raw scores were used in all models as previously published (144). Components 11 and 19 were selected in cord DNAm data. As seen in other models, some components (in this case, Components 9 and 18) were only selected in DNAm data collected at later ages, which may be due to greater power in these data sets. We note that Components 9 and 19 are enriched for molecular markers that are specific to neuroectodermal cell lineages (Figure S18 and in main manuscript, Figure 8.) Data presented in Components 1-6 and 10 are not discussed due to strong relations to confounders.

Below, we show models for intelligence outcomes measured using the Wechsler Intelligence Scale for Children-III ^UK^ (WISC). All models for WISC performed poorly at age 4 (i.e., large mean squared error (MSE) and/or a small or negative coefficient of determination (R^2^)). However, of the 914 subjects with cord blood DNAm data, 791 (86.5%) had missing WISC data at age 4 compared to only 53 (5.8%) at age 8. Models for WISC at age 8 performed comparably to other outcomes. In Figure S12, WISC data at age 8 models are shown.


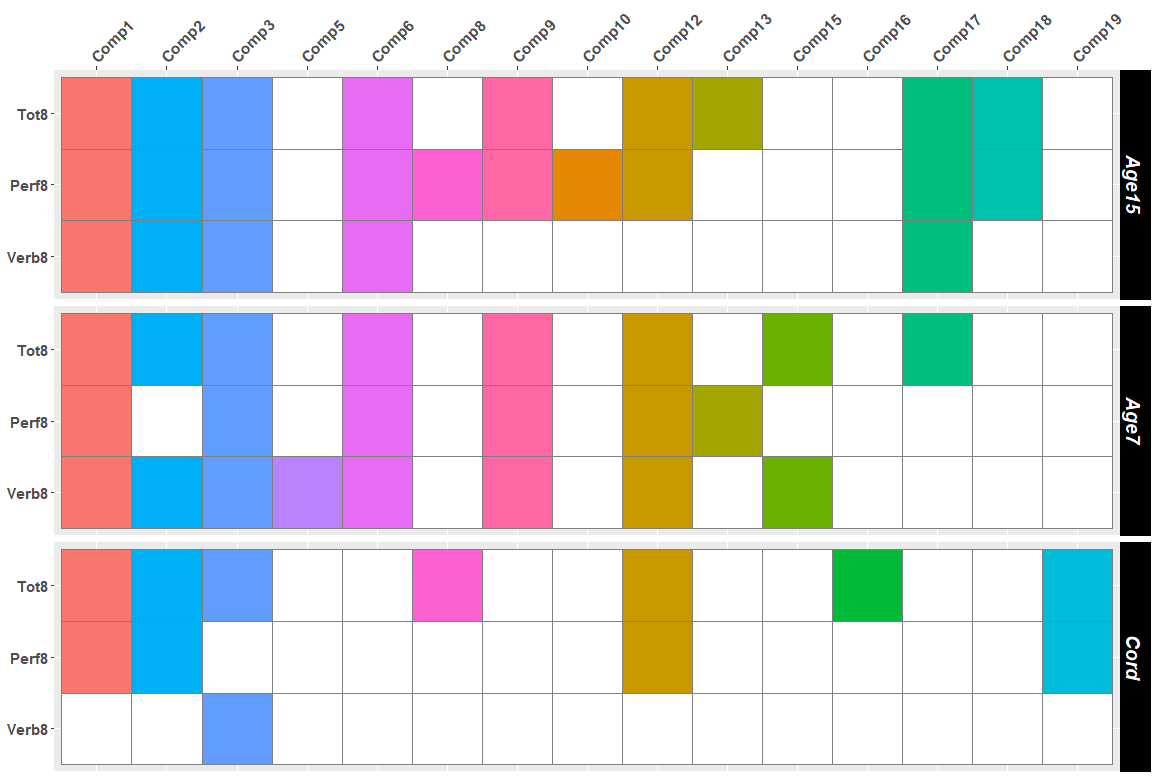


**Figure S12: WISC performance at age 8 years.** Subtest scores for this test were calculated to create a Verbal IQ, Performance IQ, and Total IQ (145) score (indicated on the y-axis as prefix Verb, Perf and Tot, respectively). Component 19 was selected in cord DNAm data, and Components 9 and 18 were selected in DNAm data collected in later childhood. The proximity in age to the WISC measurements and DNAm collection, however, could argue that reverse causality drives this relation. Data found in Components 1-6 and 10 are not discussed due to their strong relations to confounders.

# Sensitivity analysis

We performed a sensitivity analysis of random forest (RF) models using DNAm data to compare three models: 1) Raw - using all variables but only observations with non-missing values, 2) Boruta - using only Boruta selected variables but only observations with non-missing values, and 3) Imputed - using all variables but all subjects with DNAm data with missing data imputed.

To facilitate comparison, we performed this analysis using the *randomForestSRC* R-package, which has a built-in impute function. Table S1 shows a comparison between these three models using waist circumference at age 10 as an example. Comparing the Raw and Impute models provides the greatest impact of changing sample size. Comparing Boruta to the other two models gives insight into the effect of including and excluding subjects with missing data, as well as including potentially irrelevant variables.

|  | Raw | Boruta selected | Data imputation |
| --- | --- | --- | --- |
| Sample size | 805 | 862 | 914 |
| Number of trees | 5000 | 5000 | 5000 |
| Forest terminal node size | 5 | 5 | 5 |
| Average no. of terminal nodes | 106.291 | 116.1642 | 120.7975 |
| No. of variables tried at each split | 11 | 2 | 11 |
| Total no. of variables | 33 | 5 | 33 |
| Resample size used to grow trees | 509 | 545 | 578 |
| Number of random split points | 10 | 10 | 10 |
| R^2^ | 15.95 | 20.15 | 15.32 |
| Performance error | 0.79 | 0.76 | 0.77 |

**Table S1: Random forest metrics – a comparison of three models using cord blood DNAm components and waist circumference as the outcome.** Raw – Without Boruta preselection, Data imputation – With data imputation without Boruta preselection using built-in *impute* function. R-package: *randomForestSRC*.

# gwDNAm patterns generalise over time and across populations

## Replication in the ARIES cohort – peripheral blood samples in later childhood

We tested models of DNAm components trained in cord blood in data from blood collected at ages 7 and 17 years. The figures below show the observed versus predicted values for waist circumference at age 10 from Model 3 i.e., include all the same control variables but use DNAm components and cell count estimates from peripheral blood DNAm data drawn at either age 7 or age 17 years. The performance at these two later ages is similar. This may indicate that despite the shifts in DNAm data due to biological variability (e.g., physiological child development including changes in blood cell count proportions) and/or artefacts (e.g., samples at later ages could be from either buffy coat or whole blood samples), the model trained in cord blood maintained the overall relation to the outcome.


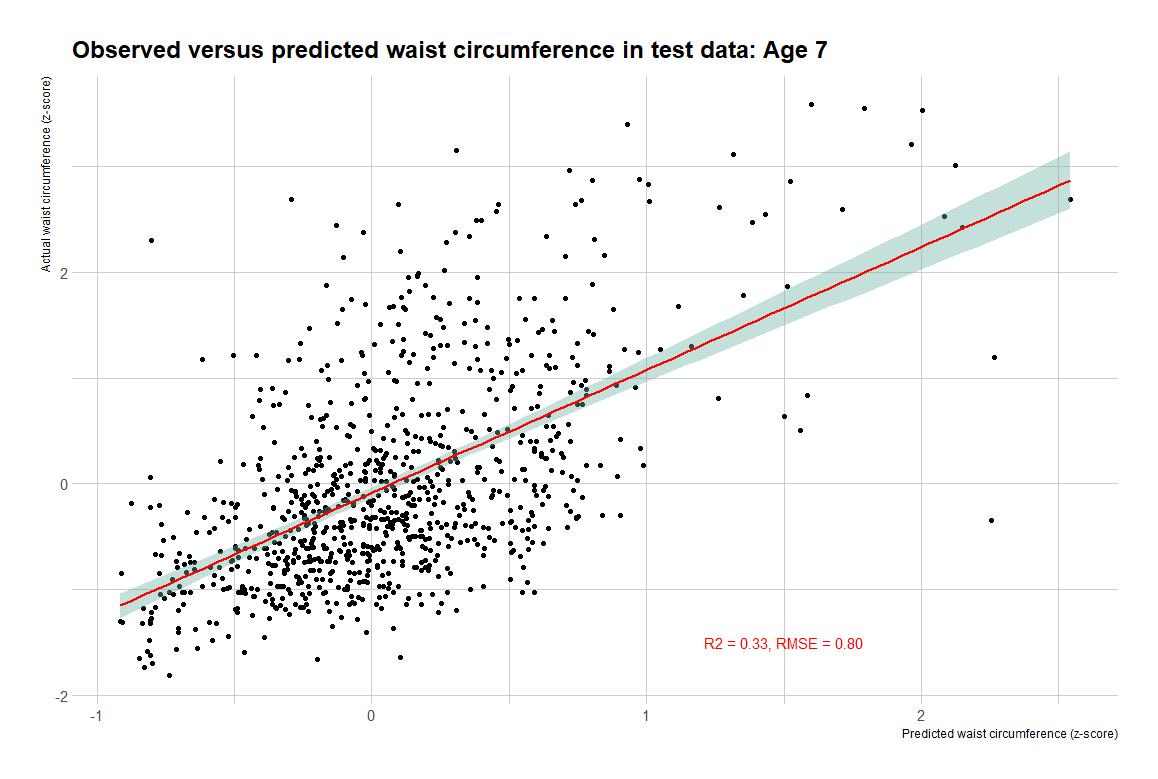

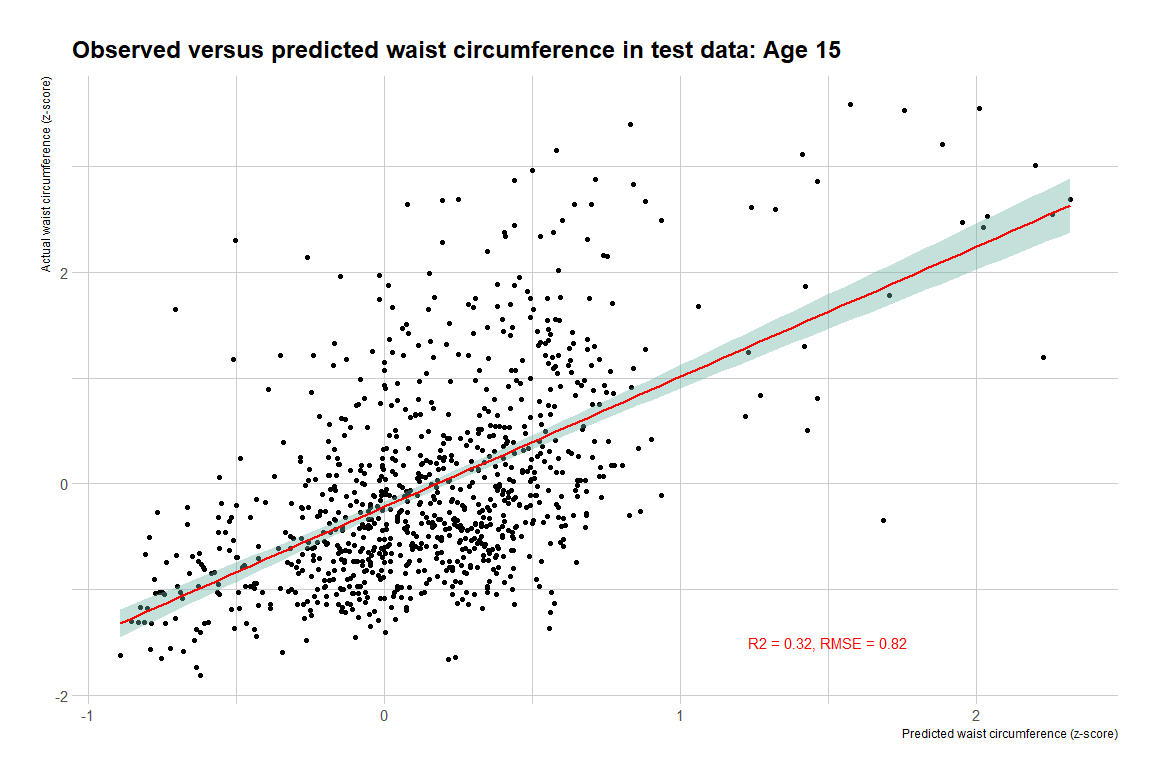


**Age 17**

**Age 7**

**Figure S13: Replication of gwDNAm patterns at birth– Model testing in peripheral blood at age 7 (top) and age 17 (bottom).** Scatterplot showing observed (y-axis) versus predicted (x-axis) values of waist circumference at age 10. The red line represents the smoothed best-fit line, and the green shading represents the 95% confidence interval. Incomplete test data was removed to run the *predict* function in the caret R-package. The performance at these two later ages is similar.

We also used the performance data provided by the built-in function *train* (using five-fold cross-validation with three repeats). The following tables show these metrics to compare model performance for cardiometabolic and neurocognitive outcomes using ARIES data from 1) cord blood, 2) peripheral blood at age 7 years, and 3) peripheral blood at age 17 years.

| Waist circumference | | |  |  |  |  |  |  |  |  |  |  |
| --- | --- | --- | --- | --- | --- | --- | --- | --- | --- | --- | --- | --- |
|  |  |  |  |  |  |  |  |  |  |  |  |  |
|  | Cord blood sample | | | | Age 7 peripheral blood | | | | Age 17 peripheral blood | | | |
|  | MSE | MSE.SD | R2 | R2 SD | MSE | MSE.SD | R2 | R2 SD | MSE | MSE.SD | R2 | R2 SD |
| Age 7 | 0.82 | 0.05 | 0.27 | 0.05 | 0.82 | 0.06 | 0.27 | 0.06 | 0.87 | 0.05 | 0.18 | 0.05 |
| Age 9 | 0.87 | 0.05 | 0.20 | 0.03 | 0.88 | 0.06 | 0.19 | 0.06 | 0.88 | 0.03 | 0.18 | 0.07 |
| Age 10 | 0.88 | 0.04 | 0.19 | 0.07 | 0.89 | 0.05 | 0.17 | 0.06 | 0.81 | 0.07 | 0.26 | 0.06 |
| Age 11 | 0.87 | 0.05 | 0.17 | 0.05 | 0.88 | 0.04 | 0.18 | 0.05 | 0.88 | 0.02 | 0.14 | 0.04 |
|  |  |  |  |  |  |  |  |  |  |  |  |  |
|  |  |  |  |  |  |  |  |  |  |  |  |  |
|  |  |  |  |  |  |  |  |  |  |  |  |  |
| Weight |  |  |  |  |  |  |  |  |  |  |  |  |
|  |  |  |  |  |  |  |  |  |  |  |  |  |
|  | Cord blood sample | | | | Age 7 peripheral blood | | | | Age 17 peripheral blood | | | |
|  | MSE | MSE.SD | R2 | R2 SD | MSE | MSE.SD | R2 | R2 SD | MSE | MSE.SD | R2 | R2 SD |
| Age 7 | 0.71 | 0.04 | 0.48 | 0.05 | 0.73 | 0.04 | 0.44 | 0.06 | 0.73 | 0.04 | 0.44 | 0.04 |
| Age 9 | 0.81 | 0.05 | 0.33 | 0.06 | 0.81 | 0.04 | 0.31 | 0.06 | 0.81 | 0.04 | 0.30 | 0.05 |
| Age 10 | 0.81 | 0.06 | 0.32 | 0.06 | 0.82 | 0.05 | 0.29 | 0.05 | 0.81 | 0.06 | 0.30 | 0.07 |
| Age 11 | 0.82 | 0.05 | 0.31 | 0.05 | 0.84 | 0.04 | 0.26 | 0.06 | 0.83 | 0.04 | 0.25 | 0.06 |
| Age 13 | 0.82 | 0.04 | 0.26 | 0.06 | 0.84 | 0.04 | 0.22 | 0.04 | 0.82 | 0.03 | 0.24 | 0.05 |
|  |  |  |  |  |  |  |  |  |  |  |  |  |
|  |  |  |  |  |  |  |  |  |  |  |  |  |
|  |  |  |  |  |  |  |  |  |  |  |  |  |
| Lean mass | |  |  |  |  |  |  |  |  |  |  |  |
|  |  |  |  |  |  |  |  |  |  |  |  |  |
|  | Cord blood sample | | | | Age 7 peripheral blood | | | | Age 17 peripheral blood | | | |
|  | MSE | MSE.SD | R2 | R2 SD | MSE | MSE.SD | R2 | R2 SD | MSE | MSE.SD | R2 | R2 SD |
| Age 9 | 0.78 | 0.03 | 0.36 | 0.05 | 0.80 | 0.03 | 0.34 | 0.06 | 0.79 | 0.02 | 0.35 | 0.05 |
| Age 11 | 0.81 | 0.04 | 0.32 | 0.08 | 0.84 | 0.02 | 0.29 | 0.05 | 0.83 | 0.03 | 0.29 | 0.05 |
| Age 13 | 0.90 | 0.03 | 0.16 | 0.04 | 0.90 | 0.02 | 0.16 | 0.04 | 0.88 | 0.03 | 0.19 | 0.06 |

| Academic achievement (National standardised test) | | | | | |  |  |  |  |  |  |  |  |
| --- | --- | --- | --- | --- | --- | --- | --- | --- | --- | --- | --- | --- | --- |
|  |  |  |  |  |  |  |  |  |  |  |  |  |  |
|  |  | Cord blood sample | | | | Age 7 peripheral blood | | | | Age 17 peripheral blood | | | |
|  |  | MSE | MSE.SD | R2 | R2 SD | MSE | MSE.SD | R2 | R2 SD | MSE | MSE.SD | R2 | R2 SD |
| K1 | Math | 1.22 | 0.04 | 0.02 | 0.01 | 1.23 | 0.07 | 0.01 | 0.01 | 1.20 | 0.05 | 0.03 | 0.03 |
|  | Reading | 1.32 | 0.05 | 0.03 | 0.03 | 1.29 | 0.05 | 0.04 | 0.02 | 1.28 | 0.04 | 0.03 | 0.02 |
|  | Writing | 1.13 | 0.04 | 0.05 | 0.04 | 1.16 | 0.03 | 0.02 | 0.01 | 1.14 | 0.03 | 0.03 | 0.02 |
|  | Summary | 3.24 | 0.15 | 0.04 | 0.02 | 3.28 | 0.14 | 0.02 | 0.02 | 3.18 | 0.11 | 0.05 | 0.03 |
|  | English | 13.66 | 0.57 | 0.05 | 0.03 | 14.07 | 0.89 | 0.04 | 0.03 | 13.85 | 0.65 | 0.04 | 0.02 |
| K2 | Math | 18.81 | 0.73 | 0.04 | 0.02 | 18.72 | 0.77 | 0.04 | 0.02 | 18.90 | 0.75 | 0.02 | 0.02 |
|  | Science | 10.07 | 0.47 | 0.04 | 0.03 | 10.22 | 0.42 | 0.03 | 0.03 | 9.96 | 0.26 | 0.05 | 0.02 |
|  | English | 15.02 | 0.57 | 0.07 | 0.03 | 15.35 | 0.65 | 0.05 | 0.03 | 15.01 | 0.73 | 0.06 | 0.04 |
| K3 | Math | 21.03 | 0.76 | 0.03 | 0.02 | 21.27 | 0.63 | 0.02 | 0.02 | 21.86 | 0.84 | 0.01 | 0.01 |
|  | Science | 21.92 | 1.18 | 0.05 | 0.05 | 22.49 | 0.90 | 0.01 | 0.01 | 22.25 | 0.72 | 0.01 | 0.01 |
|  |  |  |  |  |  |  |  |  |  |  |  |  |  |
|  |  |  |  |  |  |  |  |  |  |  |  |  |  |
| Infant Development (Denver Developmental Screening test – modified) | | | | | | | |  |  |  |  |  |  |
|  |  |  |  |  |  |  |  |  |  |  |  |  |  |
|  |  | Cord blood sample | | | | Age 7 peripheral blood | | | | Age 17 peripheral blood | | | |
|  | Age (months) | MSE | MSE.SD | R2 | R2 SD | MSE | MSE.SD | R2 | R2 SD | MSE | MSE.SD | R2 | R2 SD |
|  | 6 | 13.1233 | 0.58051 | 0.02097 | 0.01625 | 13.4616 | 0.51688 | 0.0055 | 0.00832 | 13.3234 | 0.50809 | 0.00188 | 0.00234 |
|  | 18 | 9.76961 | 0.54866 | 0.01911 | 0.01676 | 9.61381 | 0.29972 | 0.0282 | 0.02324 | 9.55495 | 0.52671 | 0.03323 | 0.02274 |
|  | 30 | 7.06262 | 0.28492 | 0.0574 | 0.02502 | 6.9823 | 0.17095 | 0.06205 | 0.02525 | 7.07946 | 0.25798 | 0.04268 | 0.02933 |

**Table S2: Performance metrics comparing models in DNAm data (Model 3) at birth and mid- and late childhood in ARIES.** Columns – age of DNAm data collection from blood. Rows – the age at the time of outcome measurement. All cardiometabolic outcomes use z-score by sex values. For brevity, only some results and MSE and R^2^ are shown. Other outcomes and additional metrics are available upon request.

To compare this performance with that found using risk-related variables alone, the table below complements Table S2 (above) and provides metrics of Model 1 (i.e., individual MRP-related variables ) as well as relevant variables selected by random forests.

| Waist circumference | | |  |  |  |
| --- | --- | --- | --- | --- | --- |
|  |  |  |  |  |  |
|  | Cord blood sample | | | | |
|  | MSE | MSE.SD | R2 | R2 SD | Variables selected |
| Age 7 | 0.80 | 0.04 | 0.28 | 0.06 | Birth weight, zwres1, zwres2, zwres3 |
| Age 9 | 0.87 | 0.05 | 0.20 | 0.07 | Birth weight, zwres1, zwres2, zwres3 |
| Age 11 | 0.80 | 0.04 | 0.28 | 0.06 | zwres1, zwres2, zwres3 |

Weight

|  | Cord blood sample | | | | | |
| --- | --- | --- | --- | --- | --- | --- |
|  | MSE | MSE.SD | R2 | R2 SD | | Variables selected |
| Age 7 | 0.69 | 0.03 | 0.48 | 0.04 | Birth weight, zwres1, zwres2, zwres3 | |
| Age 9 | 0.80 | 0.06 | 0.32 | 0.06 | Birth weight, zwres1, zwres2, zwres3 | |
| Age 10 | 0.80 | 0.05 | 0.32 | 0.05 | Birth weight, zwres1, zwres2, zwres3 | |
| Age 11 | 0.81 | 0.05 | 0.30 | 0.08 | Birth weight, zwres1, zwres2, zwres3 | |
| Age 13 | 0.83 | 0.03 | 0.23 | 0.06 | Birth weight, zwres1, zwres2, zwres3 | |

| Lean mass | |  |  | |  |  |  |
| --- | --- | --- | --- | --- | --- | --- | --- |
|  |  |  |  | |  |  |  |
|  | Cord blood sample | | | | | | |
|  | MSE | MSE.SD | | R2 | R2 SD |  | Variables selected |
| Age 9 | 0.78 | 0.04 | 0.36 | | 0.07 | Birth weight, zwres1, zwres2, zwres3 | |
| Age 11 | 0.78 | 0.05 | 0.36 | | 0.08 | Birth weight, zwres1, zwres2, zwres3 | |
| Age 13 | 0.83 | 0.02 | 0.29 | | 0.05 | Birth weight, zwres1, zwres2, zwres3 | |

| **Age period** | **Average change in weight** |
| --- | --- |
| Birth to 3 months | zwres1 |
| 3 to 12 months | zwres2 |
| 12 to 36 months | zwres3 |

Academic achievement (National standardised test)

|  |  | Cord blood sample | | | | |
| --- | --- | --- | --- | --- | --- | --- |
|  |  | MSE | MSE.SD | R2 | R2 SD | Variables selected |
| K1 | Math | 1.19 | 0.05 | 0.03 | 0.02 | Paternal social status |
|  | Reading | 1.28 | 0.06 | 0.04 | 0.04 | Paternal social status |
|  | Writing | 1.10 | 0.05 | 0.06 | 0.04 | Paternal social status, sex, **maternal smoking in pregnancy** |
|  | Summary | 3.17 | 0.15 | 0.05 | 0.03 | Paternal social status |
|  | English | 13.27 | 0.43 | 0.09 | 0.04 | Paternal social status, sex, |
| K2 | Math | 18.17 | 1.01 | 0.07 | 0.05 | Paternal social status, **maternal grandmother and grandfather smoking status** |
|  | Science | 9.82 | 0.50 | 0.06 | 0.03 | Paternal social status |
|  | English | 14.73 | 0.69 | 0.09 | 0.04 | Paternal social status, sex |
| K3 | Math | 20.55 | 0.86 | 0.06 | 0.04 | Paternal social status |
|  | Science | 22.19 | 0.78 | 0.02 | 0.02 | Paternal social status |

Infant Development (Denver Developmental Screening test – modified)

|  | Cord blood sample | | | | |
| --- | --- | --- | --- | --- | --- |
| Age (months) | MSE | MSE.SD | R2 | R2 SD | Variables selected |
| 6 | null | null | null | null | None |
| 18 | 9.44 | 0.48 | 0.05 | 0.03 | Sex |
| 30 | 6.83 | 0.27 | 0.08 | 0.04 | Sex |

**Table S3: Performance metrics of Model 1 (risk-related variables) in ARIES.** We used the performance data provided by the built-in function in *train* (using five-fold cross-validation with three repeats.) Rows – the age at the time of measurement. All cardiometabolic outcomes use z-score by sex values. Other outcomes are available upon request.

## 2) External validation in GenR cohort – cord blood samples

For GenR, body mass index (BMI) at 6 years was available for outcome analysis. Out of 969 subjects with cord DNAm data, 283 had missing BMI data and were excluded from the RF analysis. This model was compared to a similar model in ARIES, where the closest BMI data point was measured at age 7 years.

# gwDNAm patterns have distinct structural and functional molecular features

The complete list of CpGs selected by PLS analysis for each component is found in Additional File 3

Looking at the location of sites using linear genomic distance, the filtered DNAm data set used in our data analysis had higher proportion of CpGs at non-genic and/or CpG-poor regions compared to the 450K BeadChip, as well as the meta-EWAS candidates (77). This was reflected in the proportion within individual gwDNAm patterns, (data available upon request.) Consistent with previous research using high variability CpGs (a.k.a. hypervariable DNAm,) there was large overlap between methylation quantitative trait loci (mQTL) and the filtered DNAm data set (146, 147). For example, Ecker and colleagues found 64% of hypervariable DNAm in neutrophils associated with mQTLs (146). We identified a 32.9% overlap between the filtered DNAm data set and mQTL previously found in ARIES cord blood. (105) This was reflected in gwDNAm components, where about a third of CpGs representative of each component overlapped with these mQTL.

When comparing sites representative of gwDNAm patterns and epigenome-wide association study (EWAS) candidates, all analyses begin with the CpG site, which is then expanded by 250 base pairs (bps) in both directions as previously published (148, 149), i.e., total 500 bps. These neighbouring sites are usually considered to share correlated information on the methylation status of the region.

## 1) Chromatin regulation

Figure S14: Component 9 overlaps with DNase I hypersensitivity sites (DHSs) sites more than randomly expected in the genome. The red vertical line indicates the number of overlaps based on permutation testing. The green line indicates the observed number of overlaps. This was the general pattern for other components, suggesting that sites representative of gwDNAm patterns localise to areas of open chromatin that are poised for transcriptional regulation.

## 2) Motif discovery

Patterns in the DNA nucleotide sequence and published data from chromatin immunoprecipitation followed by sequencing experiments allow researchers to evaluate whether a particular genomic region may be a target for transcription factor binding. These sequences, called motifs, can be mined computationally to infer the function and interaction of that genomic region in gene regulation. The top 5 ranked motifs using the *XTREME* tool that exceeded the *p-*value thresholding conducted by Simple Enrichment Analysis are shown below. This tool references four major databases (GEO, SRA, ENCODE and modENCODE) via the Gene Transcription Regulation Database (GTRD; http://gtrd.biouml.org/).

In the following figures under the column “Motif Source”, the motif’s origin is provided in parentheses. Those with “STREME” or “MEME” are identified *ab initio,* whereas other sources are from repository-based motif databases. According to the authors, a*b initio* motifs thus provide “an unbiased view of the in vivo DNA-binding propensities” (45). These analyses were all compared with the background “control” group of sites from the meta-EWAS (44). A “hit” is only identified if the interrogated sites (in our case, sites representing a given gwDNAm pattern) can generate the same or more motifs of equal width and number of occurrences as the background sites based on log likelihood ratios (45). We show the results for Components 7, 9 and 18. Results for other components are available upon request.


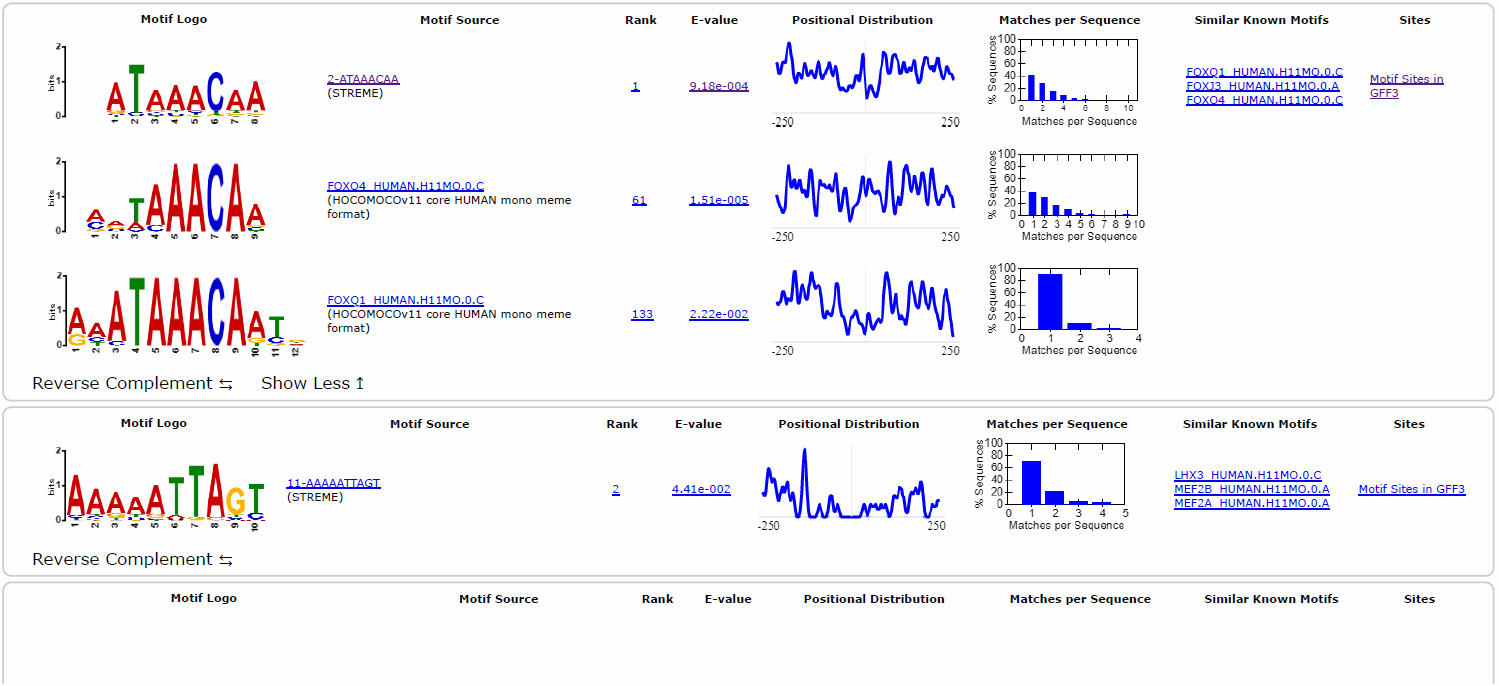


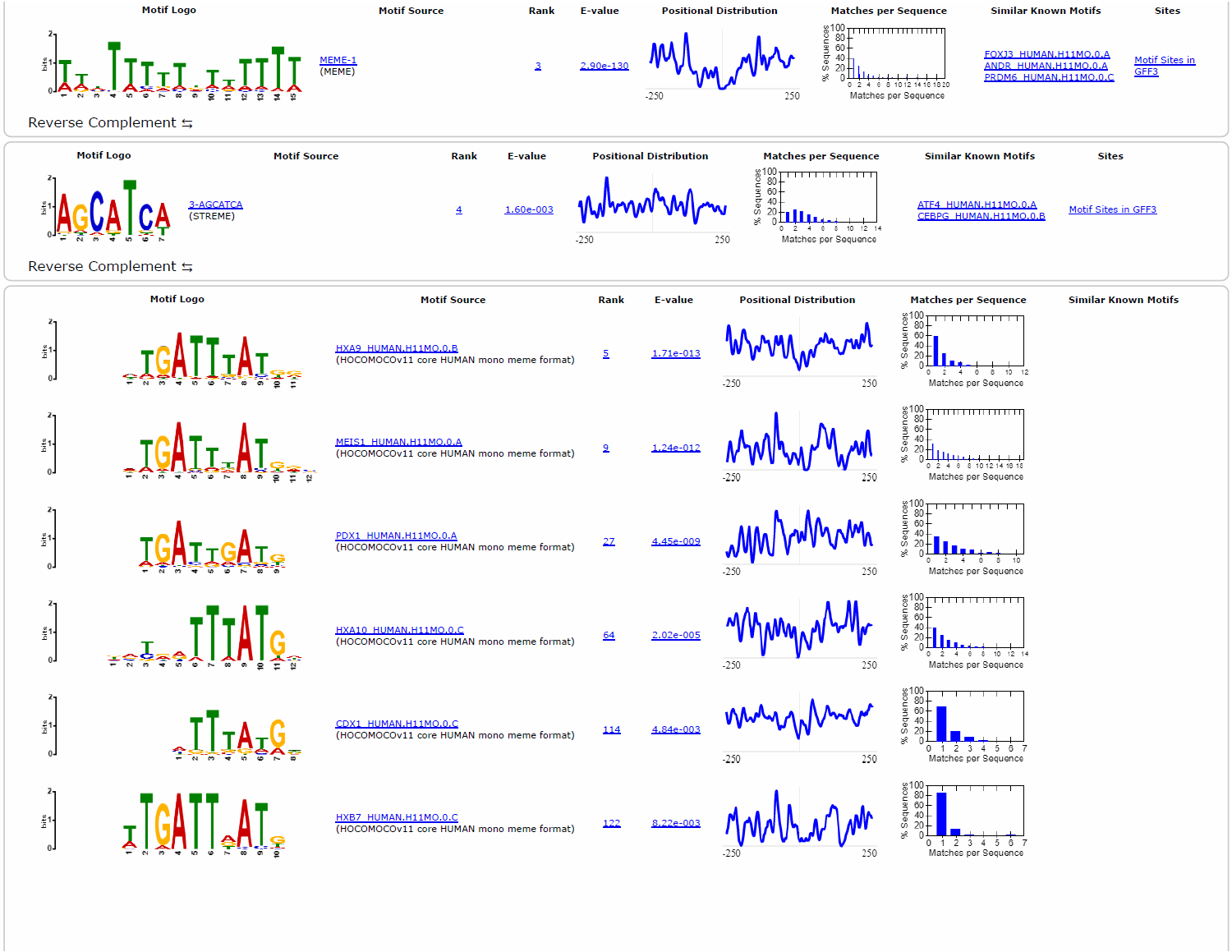


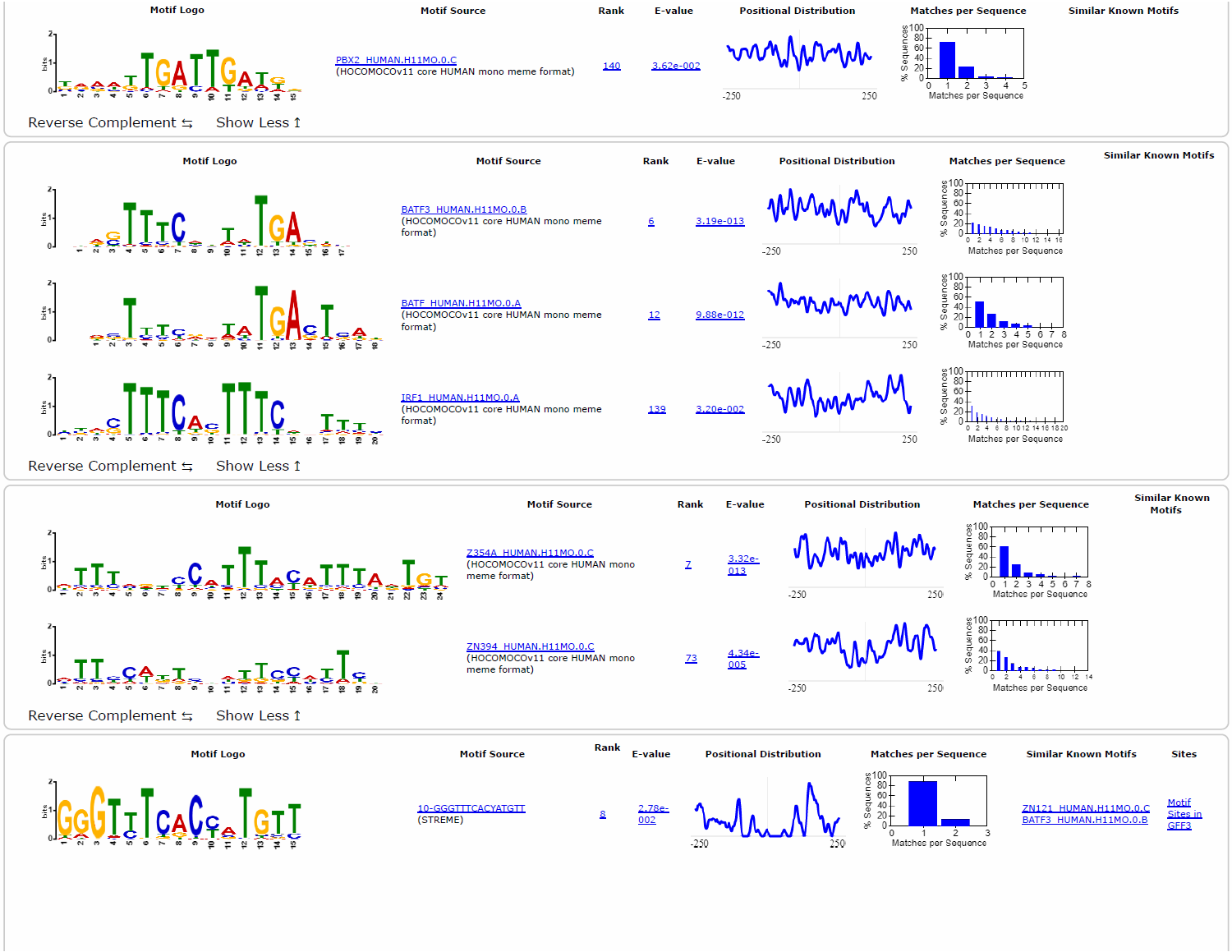


Figure S15: Component 7 versus control group: meta-EWAS (44)


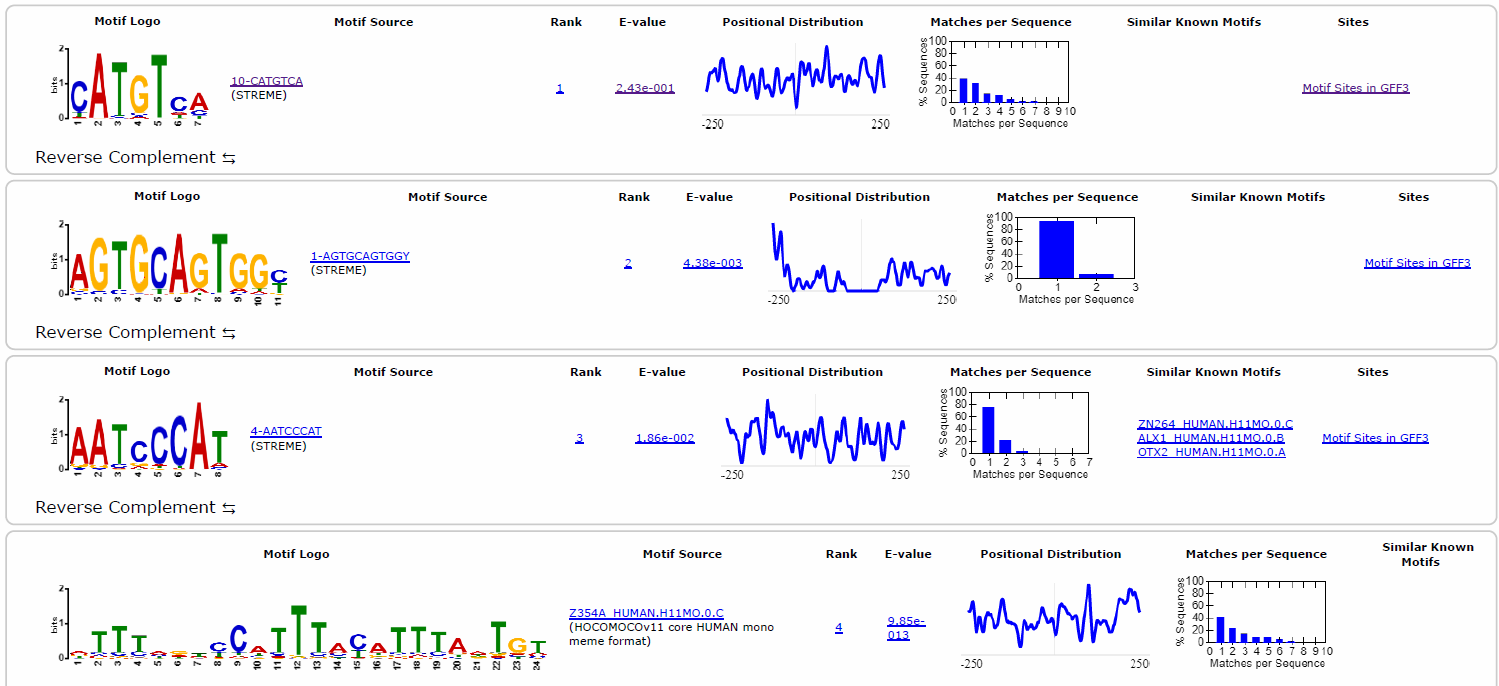


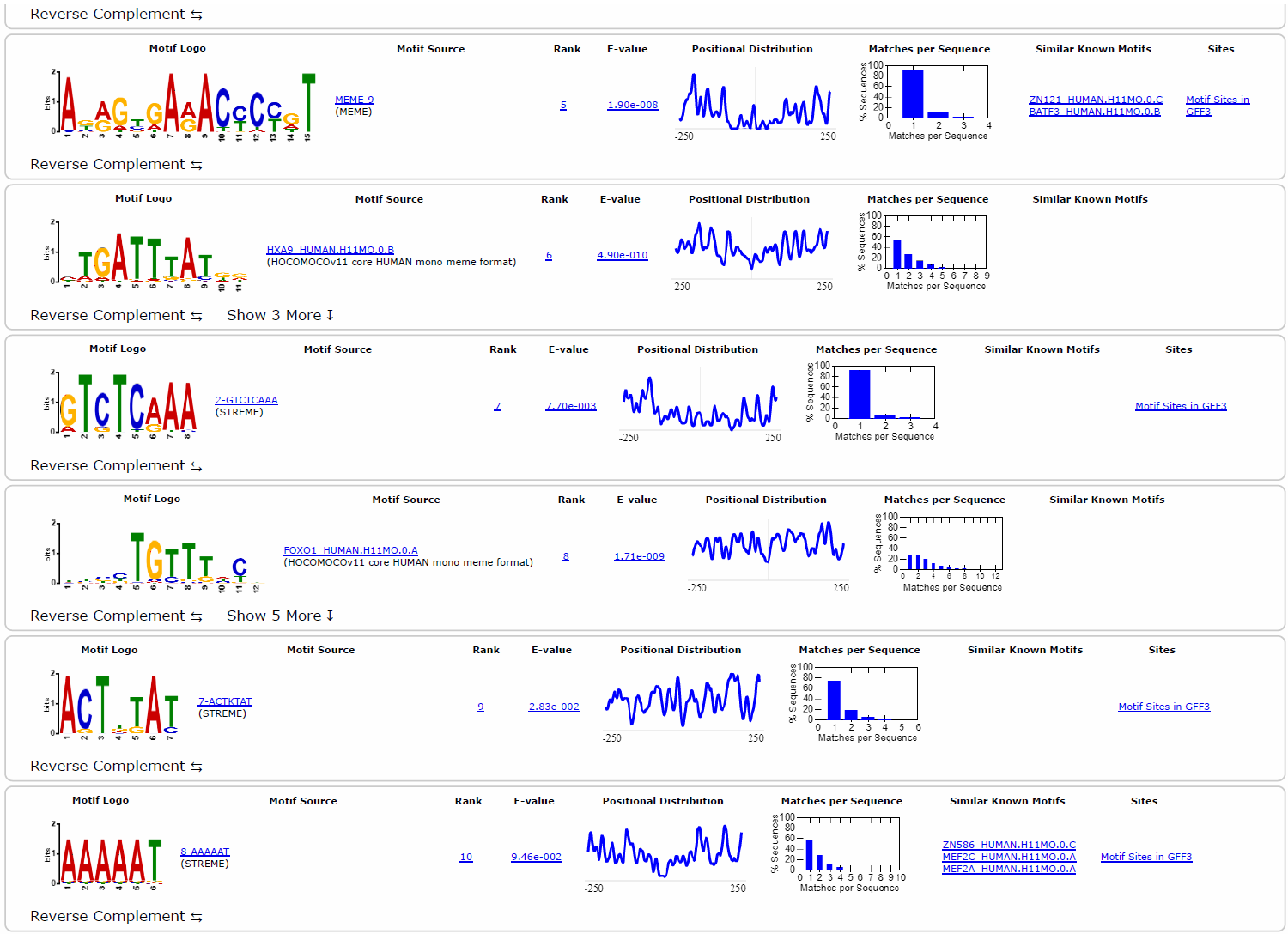


Figure S16: Component 9 versus control group: meta-EWAS (44)


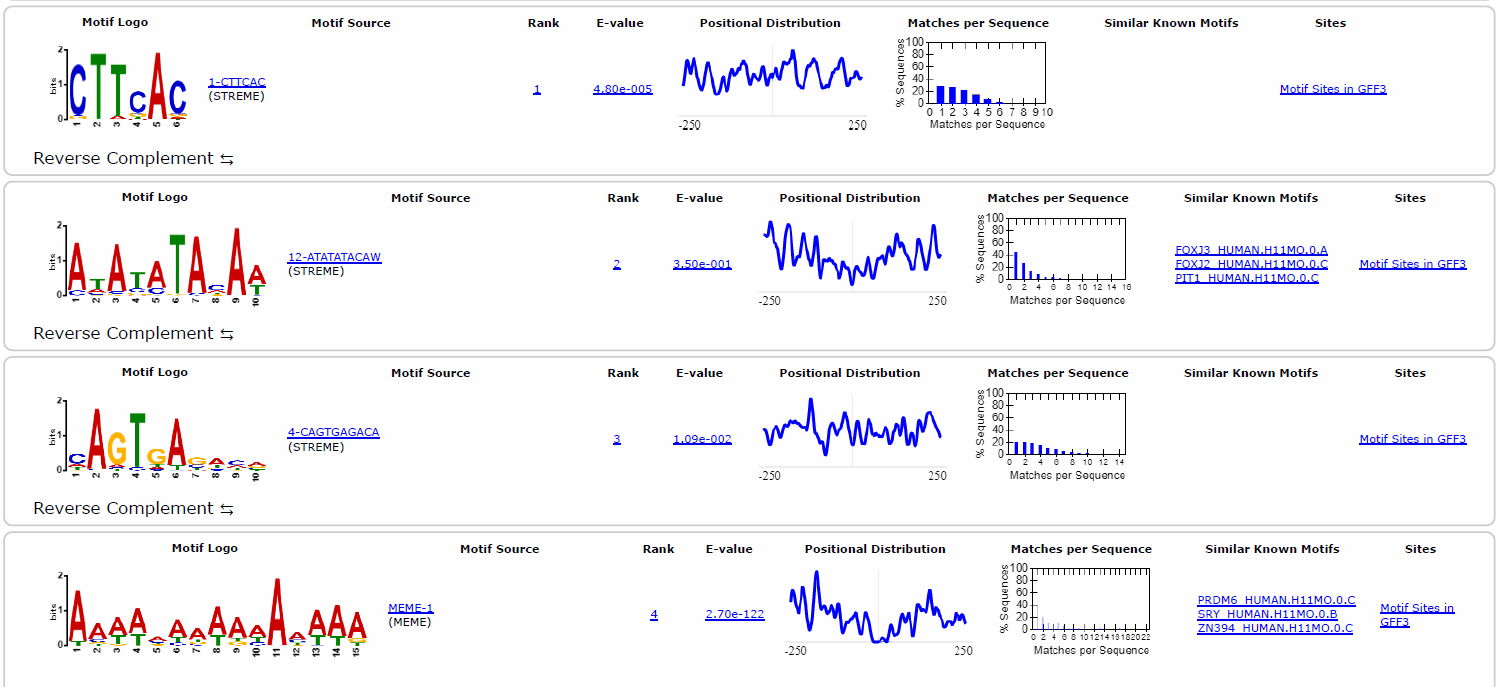

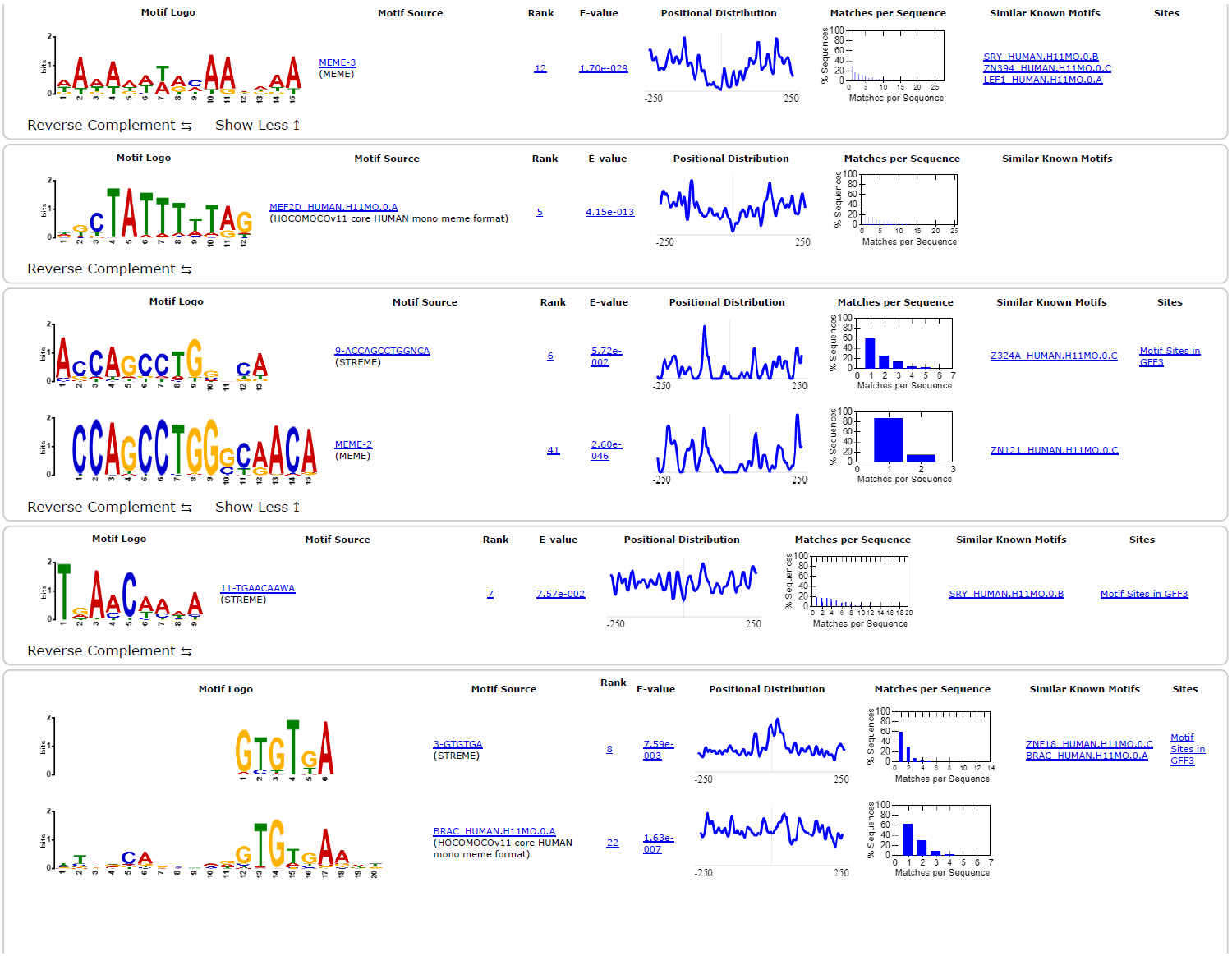

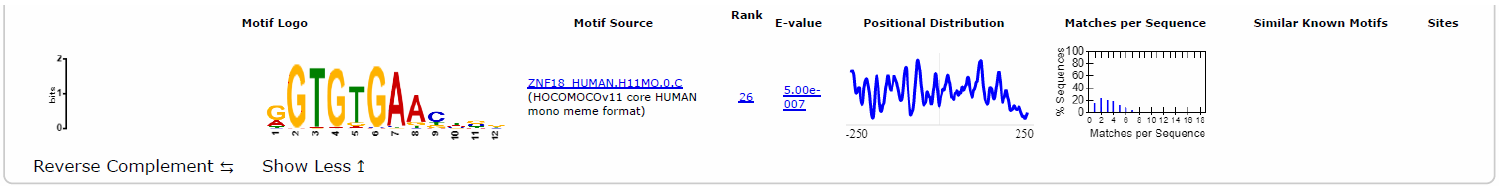


**Figure S17:** **Component 18 versus control group: meta-EWAS (44)**

## 3) Tissue specificity

We used locus overlap enrichment analysis (LOLA) to evaluate whether gwDNAm patterns localised to “hot spots” were involved in chromatin function. These areas are mapped through databases that annotate genomic locations of DNA-to-DNA or DNA-to-protein interactions that regulate chromatin activity. These annotations could be based on the local characteristics (such as histone methylation and acetylation) or footprints left by factors such as DNase enzymes. Some of these features are found in the genome of certain cell lineages, so they can indicate cell specificity. The main manuscript shows LOLA results using chromatin marks catalogued by NIH Roadmap Epigenomics. The figure below shows the same analysis but for DNaseI hypersensitivity sites (Figure S18). The clustering of sites representing a gwDNAm pattern suggests an inherent organisation that may reflect cell specificity.

For the sake of brevity, we have not shown results for all gwDNAm patterns. Component 4 was found to have a weak to moderate correlation with sex (see Figure S3), and was also enriched for sex-specific marks (e.g., using chromatin mark data, there was enrichment for the female fetal brain, see Figure S19). For this reason, we dismissed Component 4 from further analysis. Further LOLA results are available upon request.


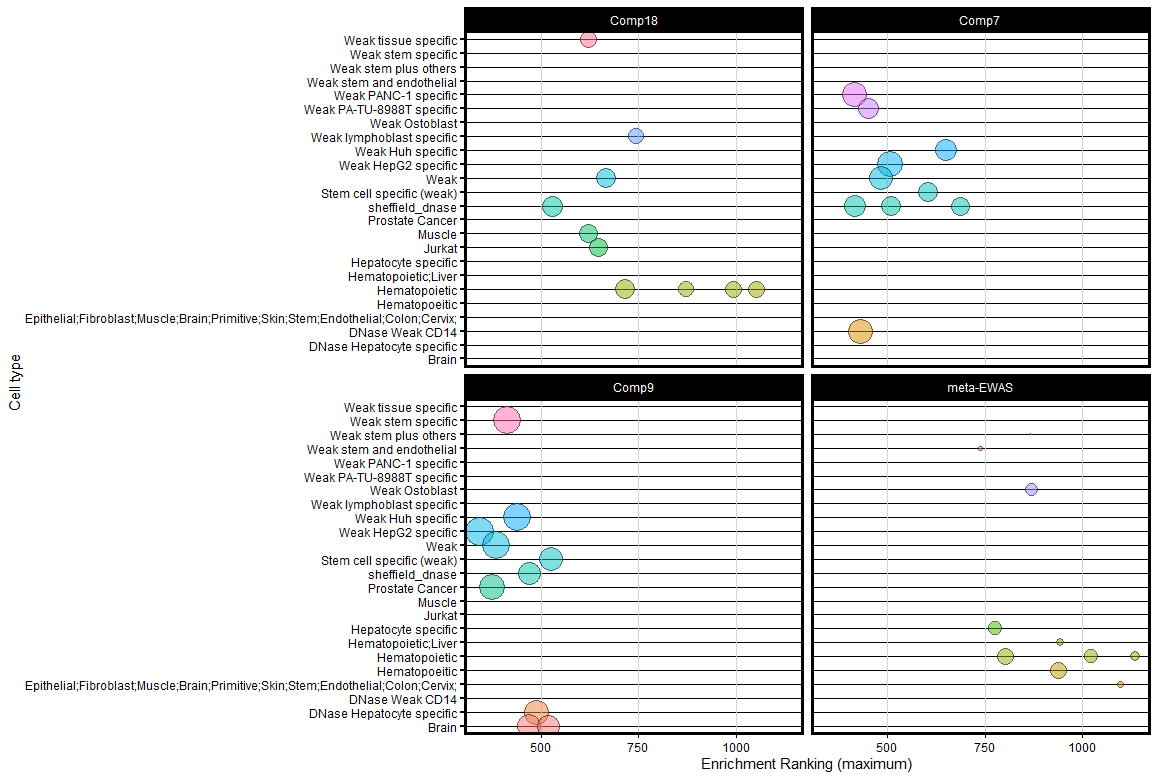


Figure S18: Locus overlap enrichment analysis (LOEA) using tissue-clustered DHSs (47) (available from LOLA core database) for meta-EWAS and Components 7, 9 and 18. X-axis: Rank (maximum) of enrichment in a given cell/tissue mark with lower numbers meaning greater enrichment. Y-axis: Cell type or tissue origin. The circle size indicates the mean enrichment rank. In this way, larger circles indicate greater enrichment calculated using a combination of three summary statistics: p-value, odds ratio, and the number of overlapping regions (46). This graph complements findings from tissue-specific chromatin marks (available in the LOLA Roadmap database) in Figure 8 of the main manuscript.


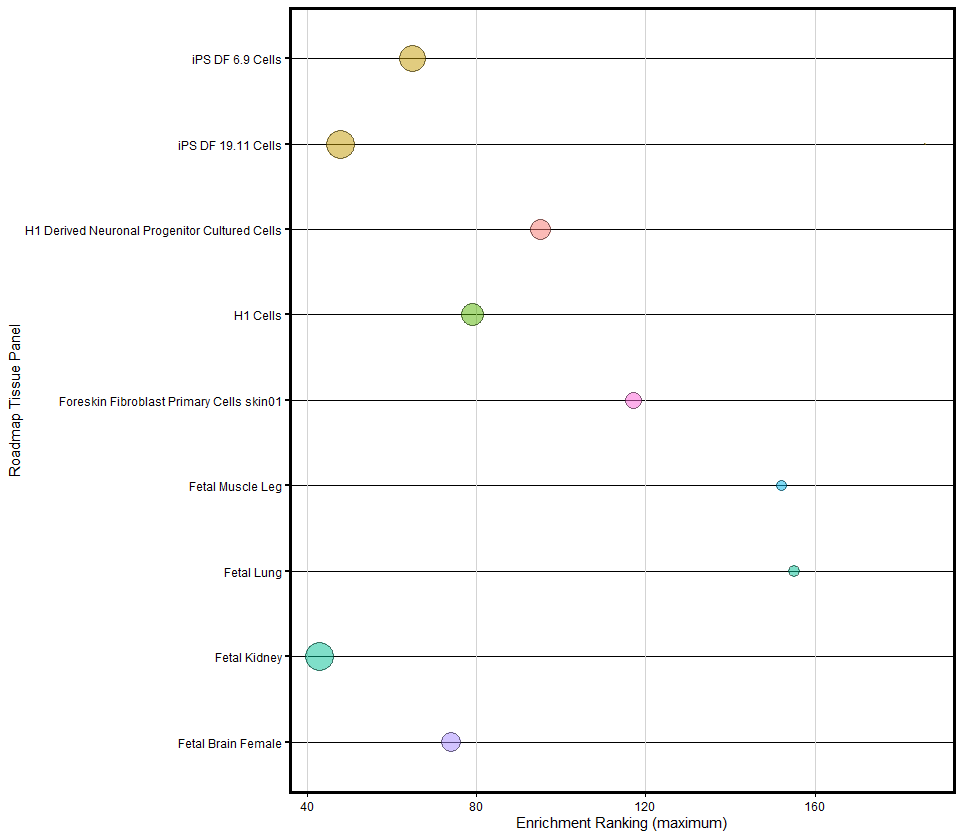


**Figure S19:** **Locus overlap enrichment analysis (LOLA) using chromatin marks (available from LOLA Roadmap database) for Component 4.** X-axis: Rank (maximum) of enrichment in a given cell/tissue mark with lower numbers meaning greater enrichment. Y-axis: Cell type or tissue origin. The circle size indicates mean enrichment rank such that larger circles indicate greater enrichment. Unlike Components 7, 9, 11, 18 and 19, but not cell-specific molecular features, which may indicate it encompasses many sources of variation that may be unrelated or a bystander to the biological phenomenon of interest i.e., risk related to maternal smoking.

# Overlap with previous literature

Like Figure 9 in the main manuscript, Figure S20 compares hits from an EWAS of maternal smoking associated with offspring cord DNAm. Richmond (48) and colleagues identified 15 CpGs, while the meta-EWAS by Joubert and colleagues found 6073 CpGs (44). Like in the meta-EWAS, a large proportion of hits found by Richmond and colleagues that were “consistent” with our study fell on Component 4. This component was related to sex (Figure S3 and Figure S19) and strongly correlated with the maternal smoking-related MRP, Dimension 2. Component 4 was also observed to be related to several future outcomes but these were dismissed as outlined above in Section: Relation of DNA patterns to confounders and maternal risk profiles.


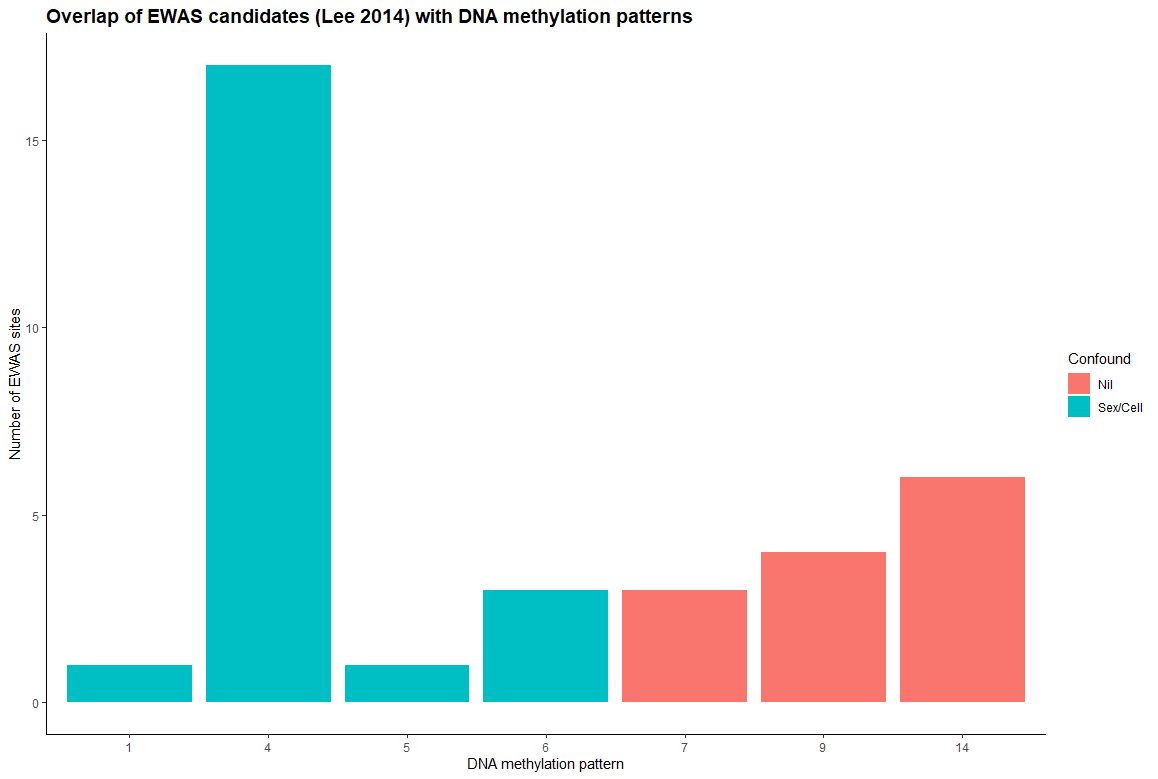


DNAm component

**Figure S20**: **All CpG sites overlapping between EWAS candidates from Richmond (48) and each DNAm component.** Each pattern was independently compared to EWAS candidates, so a site may be represented more than once. Blue bars indicate the sites that overlap with sex or cell count. Red bars indicate no known association with a confounder.
